# Multiplex Detection and Quantification of Virus Co-Infections Using Label-free Surface-Enhanced Raman Spectroscopy and Deep Learning Algorithms

**DOI:** 10.1021/acssensors.4c03209

**Published:** 2025-01-28

**Authors:** Yanjun Yang, Jiaheng Cui, Amit Kumar, Dan Luo, Jackelyn Murray, Les Jones, Xianyan Chen, Sebastian Hülck, Ralph A. Tripp, Yiping Zhao

**Affiliations:** † Department of Physics and Astronomy, Franklin College of Arts and Sciences, The University of Georgia, Athens, Georgia 30602, United States; ‡ School of Electrical and Computer Engineering, College of Engineering, The University of Georgia, Athens, Georgia 30602, United States; § Department of Statistics, Franklin College of Arts and Sciences, The University of Georgia, Athens, Georgia 30602, United States; ∥ Department of Infectious Diseases, College of Veterinary Medicine, The University of Georgia, Athens, Georgia 30602, United States; ⊥ Department of Epidemiology & Biostatistics, College of Public Health, The University of Georgia, Athens, Georgia 30602, United States; # Tec5USA Inc., Plainview, New York 11803, United States

**Keywords:** surface-enhanced Raman spectroscopy (SERS), silver nanorods, label-free diagnostics, virus coinfection, multiplex detection and quantification, deep learning, one model multiple tasks

## Abstract

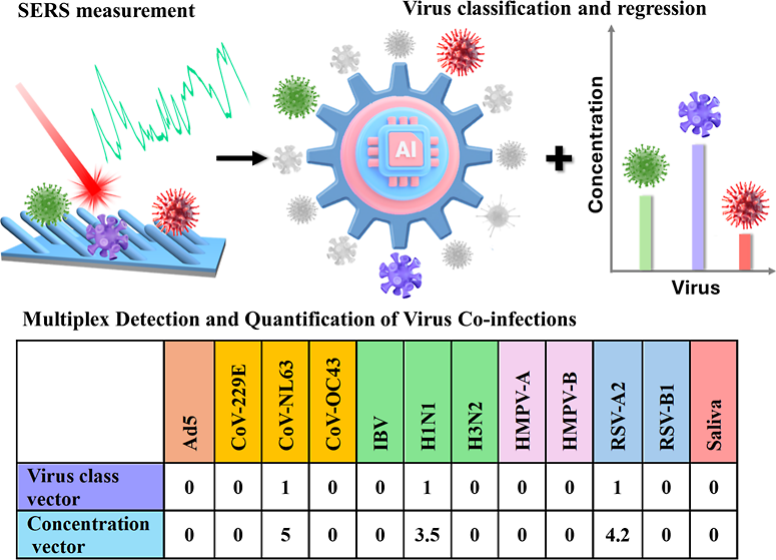

Multiple respiratory viruses can concurrently or sequentially infect the respiratory tract, making their identification crucial for diagnosis, treatment, and disease management. We present a label-free diagnostic platform integrating surface-enhanced Raman scattering (SERS) with deep learning for rapid, quantitative detection of respiratory virus coinfections. Using sensitive silica-coated silver nanorod array substrates, over 1.2 million SERS spectra are collected from 11 viruses, nine two-virus mixtures, and four three-virus mixtures at various concentrations in saliva. A deep learning model, MultiplexCR, is developed to simultaneously predict virus species and concentrations from SERS spectra. It achieves an impressive 98.6% accuracy in classifying virus coinfections and a mean absolute error of 0.028 for concentration regression. In blind tests, the model demonstrates consistent high accuracy and precise concentration predictions. This SERS-MultiplexCR platform completes the entire detection process in just 15 min, offering significant potential for rapid, point-of-care diagnostics in infection detection, as well as applications in food safety and environmental monitoring.

Virus coinfections, where an individual is simultaneously infected by two or more pathogens, can significantly increase the severity of illnesses, complicate treatment, and lead to worse health outcomes. For example, coinfections with multiple respiratory viruses can lead to severe acute respiratory infections (SARI). During the COVID-19 pandemic, it was found that SARS-CoV-2 infection sometime occurred with influenza, RSV, or adenoviruses coinfections.^1^ The probability of patients with respiratory tract illness of more than one virus can be as high as 35%.^2^ Co-infections can result in atypical symptoms, mask the presence of one virus, and lead to more severe outcomes. Studies found that around 19.7% of hospitalized COVID-19 patients had a secondary infection, and those with coinfections had a mortality rate of 16%, compared to 6% in those without coinfections.^3^ Additionally, HIV and hepatitis C virus (HCV) coinfection affects approximately 2.3 million people globally, which can lead to a higher risk of liver-related complications and liver cancer, with an estimated 3–5 times greater risk of progression to liver cirrhosis compared to HCV alone.^4^ Therefore, clearly identifying coinfections allows for targeted treatment plans, reduces the risk of misdiagnosis, and helps predict disease course. It also aids in implementing preventive measures, controlling transmission, and limiting unnecessary antibiotic use. Furthermore, studying coinfections enhances our understanding of viral interactions, immune responses, and the emergence of novel combinations. Therefore, it is important to detect the potential multiviral infection from a patient specimen.

Some of the current methods for detecting virus coinfection include multiplex polymerase chain reaction (PCR) assays,^5−8^ serological tests,^9^ next-generation sequencing (NGS),^10,11^ and other methods. Multiplex PCR panels are sensitive and specific but can be limited by the number of detectable targets. This method needs to design specific probes or primers to target potential viruses. For example, several respiratory viral species can be analyzed tested in diagnostic laboratories, including influenza viruses, respiratory syncytial viruses (RSV), rhinoviruses, coronaviruses (including strains like CoV-229E, CoV-NL63, CoV-OC43, and SARS-CoV-2), adenoviruses human metapneumovirus (HMPV), parainfluenza viruses (types 1–4), enteroviruses, and human bocavirus.^12^ Having multiple detection requirements also make the instrument and detection system more complicated and expensive. Table S1 in Supporting Information summarizes various detection methods for virus coinfection, detailing their pros and cons, typical applications, time to result, and cost considerations. Each method presents trade-offs in sensitivity, specificity, cost, and complexity.

Surface-enhanced Raman spectroscopy (SERS) has emerged as a powerful analytical technique providing detailed molecular vibration information through fingerprint-like Raman spectra. In recent years, there are significant advancements in SERS-based virus detection using label^13,14^ or label-free detection strategies,^15,16^ especially for label-free detection, which eliminates the need for complex and time-consuming sample preparation steps. However, SERS-based label-free virus coinfection detection with classification and quantification simultaneously remains unexplored. Two different strategies exist: one is to spatially separate analytes at different locations on the SERS substrate, such as ultrathin layer chromatography (UTLC);^17^ the other is to use spectroscopic analysis methods, as different analytes have unique spectra that can be determined by advanced data analysis methods.^18^ For component identification of mixtures, many statistics and chemometric methods have been proposed, e.g., interactive self-modeling mixture analysis (SIMPLISMA),^19^ iterative target transformation factor analysis (ITTFA),^20^ independent component analysis (ICA),^21^ nonnegative matrix factorization (NMF),^22^ and so on. In addition, a data compression algorithm named characteristic peak extraction (CaPE), based on machine learning methods, has been developed,^23^ which extracts characteristic peaks from SERS spectra based on counts at locations of detected peaks of the mixture. These methods face limitations when dealing with source analytes that have similar SERS peaks in mixtures. To address this, a deep learning model called the spectral extraction neural network has been developed for extracting pure spectra of each component from mixture spectra, which shows the better performance for the analyte with similar peaks.^24^ However, as to the quantification, multiple regression models should be constructed for each source analyte one by one, which are separate from the spectral extraction neural network model.

Differentiating and quantifying virus coinfections using SERS is challenging compared to other mixtures due to several factors: (1) relatively weak SERS signals: viruses, with sizes around 100 nm, are much larger than the typical gaps at the hot-spots of SERS substrates. This size mismatch limits the exposure of the virus surface to the intense near-field electromagnetic enhancement at the hot-spot, which is crucial for generating strong SERS signals.^25^ As a result, the large size of viruses means they are located beyond the optimal distance for maximum enhancement, resulting in weaker signals compared to smaller analytes. (2) Spectral similarity: the spectral signatures of viruses often share common biomolecular components, such as proteins, lipids, and nucleic acids, which result in broad and overlapping peaks in the SERS spectra.^15^ This overlap complicates the differentiation and quantification of closely related viruses and variants, particularly when multiple viruses are present in a sample. The challenge is exacerbated by the subtle spectral differences between these viruses, making it difficult to distinguish them in complex mixtures. (3) Complex virus specimen matrices and environmental conditions can lead to spectral interference. Therefore, the complexity of SERS spectra requires sophisticated data analysis techniques. Deep learning algorithms (DLAs) offer a powerful approach to overcoming the challenges of differentiating and quantifying virus coinfections in SERS spectra. By leveraging their ability to extract meaningful features from complex, high-dimensional data, DLAs can identify subtle spectral differences caused by overlapping biomolecular signatures,^26,27^ enabling precise differentiation of closely related viruses or strains. These models are particularly well-suited for multilabel classification tasks, such as identifying what virus types are in a mixture, and for regression tasks to quantify their concentrations.^28^ Furthermore, DLAs automate SERS data analysis, enhancing sensitivity and specificity while reducing analysis time and human error. By addressing the inherent complexities of SERS spectra, such as weak signals and overlapping peaks, using automatic feature extraction modules, DLAs provide a robust, efficient, and accurate tool for virus detection in healthcare, virology, and disease surveillance.^29^

Herein, a label-free testing platform has been developed combining SERS and deep learning for the rapid classification and accurate quantification of virus coinfections. As a proof-of-concept, we utilize highly reproducible and sensitive silica-coated silver nanorod array SERS substrates alongside a portable Raman system to capture SERS spectra from 11 respiratory virus species (single viruses, i.e., SVs), 9 two-virus mixtures (2VMs), and 4 three-virus mixtures (3VMs). The spectra allow us to identify the characteristic SERS peaks of different viruses and construct a robust virus SERS spectral database. A multiplex classification and regression deep learning model, MultiplexCR, is developed to predict both the virus species and concentrations in the mixture. Through rigorous optimization, the model achieves an accuracy of 98.6 ± 0.3% for virus coinfection classification and a mean absolute error (MAE) of 0.028 ± 0.004 for virus concentration regression. These results highlight the efficacy of the SERS + DLA strategy in accurately diagnosing complex infectious specimens. Furthermore, these findings demonstrate the potential of this integrated approach for rapid virus coinfection detection, suggesting its suitability for future deployment as a point-of-care diagnostic platform.

## Experimental Section

### Design of Virus Mixture Specimens

The following 11 single viruses (SVs) were used in the study: adenovirus type 5 (Ad5), human coronavirus NL63 (CoV-NL63), human coronavirus 229E (CoV-229E), human coronavirus OC43 (CoV-OC43); influenza A H1N1 Brisbane (H1N1), influenza A H3N2 Hong Kong (H3N2), and influenza B (Flu B); human metapneumovirus (HMPV) from strain A (HMPV-A) and B (HMPV-B); as well as respiratory syncytial virus (RSV) from strain A2 (RSV-A2) and B1 (RSV-B1). For the incubation of 11 respiratory viruses, a detailed procedure is described in Section S2 of Supporting Information. Overall, 9 sets of 2VMs and 4 sets of 3VMs were designed to provide a comprehensive representation of respiratory virus coinfections, as detailed in Table S2. The selected virus mixtures were chosen based on their biological and clinical relevance, spectral diversity, and the need to demonstrate model generalization and robustness effectively. These viruses were selected due to their significant impact on respiratory health and their known potential to cocirculate, leading to coinfections that can exacerbate disease severity. Literature surveys confirm that influenza, RSV, and coronaviruses frequently cocirculate, posing serious public health risks, particularly in the context of severe respiratory illnesses.^30−34^ In terms of spectral diversity, the selected mixtures include viruses with both distinct and overlapping spectral features. For example, CoV-NL63 and Flu B show similar spectral features. This diversity ensures that the MultiplexCR model is challenged with spectrally similar pairs to evaluate its ability to differentiate subtle variations, which ensures the coverage of a wide range of spectral scenarios that might occur in practice. The mixtures were also chosen to assess the model’s generalization and robustness under varying levels of coinfection complexity. By including both 2VMs and 3VMs, the MultiplexCR’s performance in handling different complexities could be systematically evaluated. While there are many potential virus combinations, the selected sets balance biological significance and practical constraints, such as the availability of virus strains, resources, and the time required for spectra collection and validation.

To validate the proposed method for coinfection detection and quantification, the viruses were diluted in saliva with different concentrations for SERS measurement. Saliva specimens provide a noninvasive and easily collectible alternative to nasopharyngeal swabs.^35,36^ They can be self-collected and stored at room temperature for up to 48 h without losing diagnostic sensitivity, making them convenient for virus diagnostics testing.^37^ Saliva specimens used in this study were virus-free by collecting them from healthy, asymptomatic donors with no recent history of respiratory infections and screening for viral contamination using RT-PCR assays, which confirmed the absence of viral RNA. Additionally, SERS spectra of the saliva specimens were collected and validated against known profiles of saliva containing virus specimens to further confirm the absence of interfering viruses. For the SVs, the virus concentrations were prepared to be 50 to 10^5^ PFU/mL with 12 different concentrations. For 11 SVs, the total number of specimens was 132. Regarding 2VMs and 3VMs, to better understand the relationship between spectra from mixed infections and those from individual viruses, complete concentration combinations have been designed. For 2VMs, the relative and absolute concentrations of each virus in the mixture were designed according to Figure S1A. Both virus A and virus B were made into 12 different concentrations, spanning from 50 to 10^5^ PFU/mL, and each concentration of virus A (*C*_A_) will be mixed with the other 12 concentrations of virus B (*C*_B_). So, a total of 144 virus mixtures with (*C*_A_, *C*_B_) were created combinations. For nine 2VMs, the total number of mixture specimens was 1296. For 3VMs, similar to 2VMs (see Figure S1B). virus A, virus B, and virus C were made into 8 different concentrations, spanning from 195 to 10^5^ PFU/mL, and the total number of 3VMs (*C*_A_, *C*_B_, *C*_C_) was 512. For 4 sets of 3VMs, the total specimen number was 2048. Virus-inoculated infection-free human saliva specimens for SVs, 2VMs, and 3VMs, were prepared by adding known (predetermined) concentrations (Table S2 and Figure S1) of virus specimens into healthy saliva to achieve final concentrations.

### Blind Tests

To demonstrate the feasibility of the proposed detection strategy, blind tests were designed, consisting of 734 specimens from 11 SVs, nine 2VMs, and four 3VMs, with all specimens above the corresponding limit of detections (LODs). The preparation followed similar strategies, by adding random and predetermined concentrations of virus specimens into infection-free human saliva to achieve the final concentrations. However, the status of the virus specimens was not given to the operators and the MultiplexCR models. The detailed list of the blind tests is shown in Table S6.

### SERS Measurement

The details of AgNR@SiO_2_ array SERS substrate fabrication are shown in Section S2 of Supporting Information. For the SERS sample preparation, 20 mL of each virus specimen were dispensed onto the AgNR@SiO_2_ wells and incubated for 15 min. Given the dense media and saliva, each well was washed with DI water and air-dried at 20 °C. Then the SERS spectra were acquired using a Tec5USA Raman spectrometer (Tec5USA Inc.), equipped with a 785 nm excitation laser with a beam diameter of ∼100 μm, a power of 35 mW, and an acquisition time of 1 s. For each type of virus specimen, approximately 350 SERS spectra were collected, resulting in a total of 1,213,550 SERS spectra.

### Deep Learning Settings

All the SERS spectra were plotted and visualized using SpectraGuru,^38^ and preprocessed following a procedure that included baseline removal and average normalization as described in Section S4. For spectrum classification and regression, the MultiplexCR deep learning model was developed using TensorFlow version 2.10.0 in Python 3.8.12. All calculations were performed on the Georgia Advanced Computing Resource Center at the University of Georgia, utilizing GPU nodes equipped with AMD EPYC Milan third generation processors (64 cores and 1TB of RAM) and four NVIDIA A100 GPU cards. The specific architecture and training parameters of the MultiplexCR model were carefully selected and are detailed in the corresponding sections for clarity. The MultiplexCR model was run in 5 independent trials to ensure the robustness and consistency of its predictions.

## Results and Discussion

### Detection Strategy

**Figure 1:**
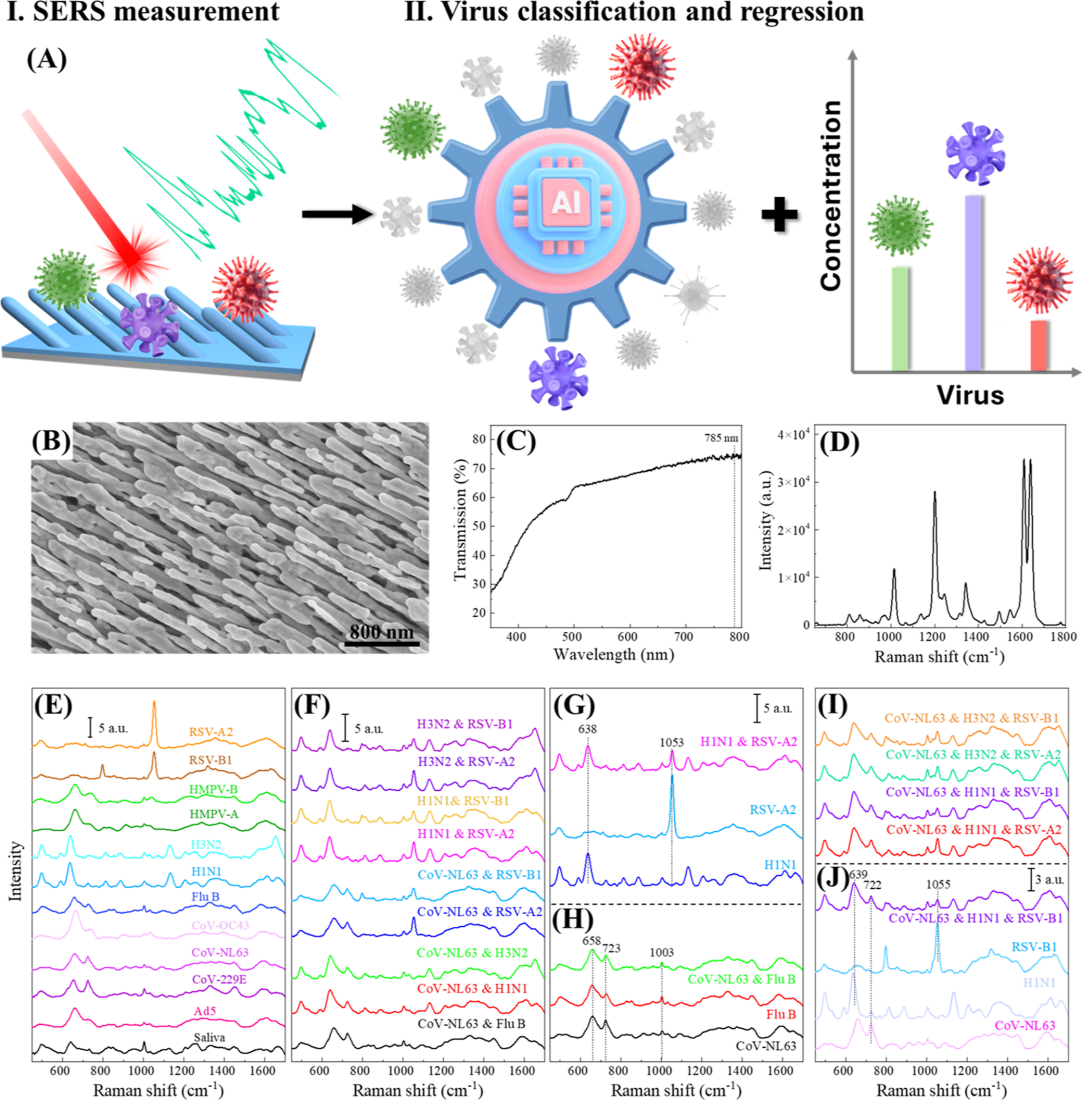
Scheme of SERS-based deep learning for virus co-infection diagnosis, AgNR@SiO_2_ substrate properties, and SERS spectra from virus specimens. (A) Schematic illustration of SERS + deep learning-based virus coinfection diagnosis: step I: SERS spectra collection from virus mixture specimens; step II: build a reliable AI model for virus classification and quantification of and their corresponding concentrations in coinfections. Characterizations of AgNR@SiO_2_ substrates: (B) a representative SEM image, (C) a UV–vis reflection spectrum, and (D) a SERS spectrum measured from 1 × 10^–5^ M BPE. SERS spectra of viruses in saliva: (E) 11 individual viruses with 10^5^ PFU/mL and saliva specimens, (F) nine 2VMs with both concentrations of 10^5^ PFU/mL, spectra comparison of (G) H1N1, RSV-A2, and the corresponding mixtures, and (H) CoV-NL63, Flu B, and the corresponding mixtures, (I) four 3VMs with respective mixture concentrations of 10^5^ PFU/mL, (J) spectra comparison of CoV-NL63, H1N1, RSV-B1, and the corresponding mixtures.

The method for integrating SERS and DLA to accurately differentiate and quantify the virus coinfection is illustrated in Figure 1A. Initially, a SERS spectral database of virus specimens is constructed by collecting spectra from AgNR@SiO_2_ SERS substrates (step I). SERS spectra are collected from 11 respiratory virus species, nine 2VMs, and four 3VMs. The spectra allow us to identify the characteristic SERS peaks of different viruses and construct a robust virus SERS spectral database. A multiplex classification and regression deep learning model “MultiplexCR” is developed to predict both the virus species and concentrations in the mixture (step II). Blind tests are performed to confirm the detection performance of both the virus species in coinfections and their corresponding concentrations.

### Understanding SERS Spectra of Virus Mixtures

The characterization of SERS substrates is shown in Figure 1B–D. Figure 1E shows the average SERS spectra of 11 SVs in saliva at 10^5^ PFU/mL and saliva reference specimens, with major SERS peaks labeled and assigned in Figure S4 and Table S3. The SERS spectra of different respiratory viruses share some common spectral features but are distinguishable based on some specific peaks. For example, the most prominent spectral peaks of CoV-229E are at Δ*v* = 657, 727, and 1325 cm^–1^, respectively, which correspond to different vibrational modes of guanine and adenine. The SERS peaks at Δ*v* = 1003 and 1031 cm^–1^ are due to phenylalanine, while peaks between Δ*v* = 1576 and 1647 cm^–1^ can be attributed to carbonyl groups on the amino acid side chains and the Amide I vibration. Influenza A viruses (H1N1 and H3N2) have two major glycoproteins on their surface, i.e., hemagglutinin and neuraminidase which are used to differentiate influenza subtypes. The main SERS peaks in their spectra appear at Δ*v* = 592 (glycine, Gly), 638 (Tyr), 887 (Gly), 1003 (phenylalanine, Phe), 1135 (ν(C–C)), 1205 (Tyr), and 1570–1662 cm^–1^, which can be assigned to vibrations of amide III, carbonyl groups from the amino acid side chains and the amide I vibration. The peak at Δ*v* = 813 cm^–1^ has been assigned to the phosphate backbone stretch of the RNA in previous bulk Raman study of viruses.^39^ The spectral features of viruses differ significantly from those of the virus growth medium and the background spectrum of the SERS substrate. Figure 1E illustrates the spectrum of saliva, while Figure S4B presents the spectra of AgNR@SiO_2_, DMEM (Dulbecco’s modified Eagle medium) buffer, and allantoic fluid. These spectra show minimal overlap with virus-specific spectral features. Additionally, the stability of virus-saliva specimens for SERS measurements is confirmed, as demonstrated in Figure S5.

The representative average SERS spectra of 11 SVs due to different concentrations are plotted in Figure S6. The characteristic virus peaks are more distinct in high-concentration specimens. For example, SERS peaks at Δ*v* = 658, 724, and 1576 cm^–1^ are observed from 10^5^ PFU/mL CoV-229E specimens (Figure S6B). As the viral concentration decreases, the relative intensities of these peaks decrease, and the SERS spectrum gradually evolves to spectrum similar to that of the saliva reference with a distinctive peak at Δ*v* = 1004 cm^–1^. Similar observations are obtained for other SVs as shown in Figure S6.

The representative average SERS spectra of 9 sets of 2VMs with (10^5^, 10^5^) PFU/mL are shown in Figure 1F. They are quite different from those of SVs if the spectra of two mixed SVs are different. For example, Figure 1G shows the average spectra of RSV-A2 & H1N1 (10^5^, 10^5^ PFU/mL), RSV-A2 (10^5^ PFU/mL), and H1N1 (10^5^ PFU/mL). Two distinguished peaks, Δ*v* = 638 and 1136 cm^–1^, unique to H1N1 (blue curve), appear in the mixture spectrum; while the Δ*v* = 1053 cm^–1^ peak specific to RSV-A2 (sky blue curve), also appears in the spectrum of the mixture. However, if the spectra of SVs are similar, the resulting spectrum from the mixture is also similar to the spectrum of an SV, Figure 1H plots the average spectra of CoV-NL63 (10^5^ PFU/mL) and Flu B (10^5^ PFU/mL), who have very similar spectral shapes. The average spectrum from their mixture (10^5^, 10^5^ PFU/mL) exhibits similar spectral features, with characteristic peaks at Δ*v* = 661, 724, 1003, and 1450 cm^–1^, respectively.

The average SERS spectra of four 3VMs (10^5^, 10^5^, 10^5^ PFU/mL) are shown in Figure 1I. Similar to those of 2VMs, the spectrum of the mixture demonstrates a linear combination of spectral features of 3 SVs. For example, Figure 1J shows the average SERS spectra of CoV-NL63 (10^5^ PFU/mL), H1N1 (10^5^ PFU/mL), RSV-B1 (10^5^ PFU/mL), and CoV-NL63 & H1N1 & RSV-B1 (10^5^, 10^5^, 10^5^ PFU/mL). The CoV-NL63 spectrum has characteristic peaks at Δ*v* = 661, 724, 1003, and 1450 cm^–1^, the H1N1 spectrum shows two distinguished peaks at Δ*v* = 638 and 1136 cm^–1^, while RSV-B1 processes a distinct peak at Δ*v* = 1055 cm^–1^. All these peaks also appear in the spectrum of CoV-NL63 & H1N1 & RSV-B1.

Concentration-dependent analysis of SERS spectra from virus mixtures is shown in Section S6. The relationships between the SERS spectra of SVs, 2VMs, and 3VMs are illustrated in Figure S9 and corresponding discussion in Section S6. Quantifying viruses in mixtures is feasible when their characteristic peaks are distinct, as shown with H1N1 and RSV-A2, where peak intensities correlate with virus concentrations. However, overlapping peaks, as seen with CoV-NL63 and Flu B, or similar spectral profiles among variants like H1N1 and H3N2, complicate detection and quantification. For complex virus mixtures, especially at low concentrations, traditional calibration curves (Section S7) are ineffective. To address these challenges, traditional machine learning models such as random forest (RF) were applied, as discussed in detail in Section S8. Although the RF model demonstrates acceptable performance with an overall classification accuracy of 81.5% and an *R*^2^ of 0.874 for regression, its performance is suboptimal for certain single viruses and virus mixtures. For example, misclassification rates are high in cases such as saliva misclassified as CoV-NL63 (21.2%), and 7 out of 8 of the components in 2VMs exhibit *R*^2^ values below 0.9, indicating challenges in accurately quantifying complex mixtures. Furthermore, the RF model requires 11.7 GB of storage, making it impractical for deployment on standard PCs or portable devices. These limitations suggest that the application of deep learning algorithms becomes essential to effectively differentiate and quantify these virus mixtures.

### Deep Learning for the Classification and Quantification of Virus Mixtures in Saliva

**Figure 2:**
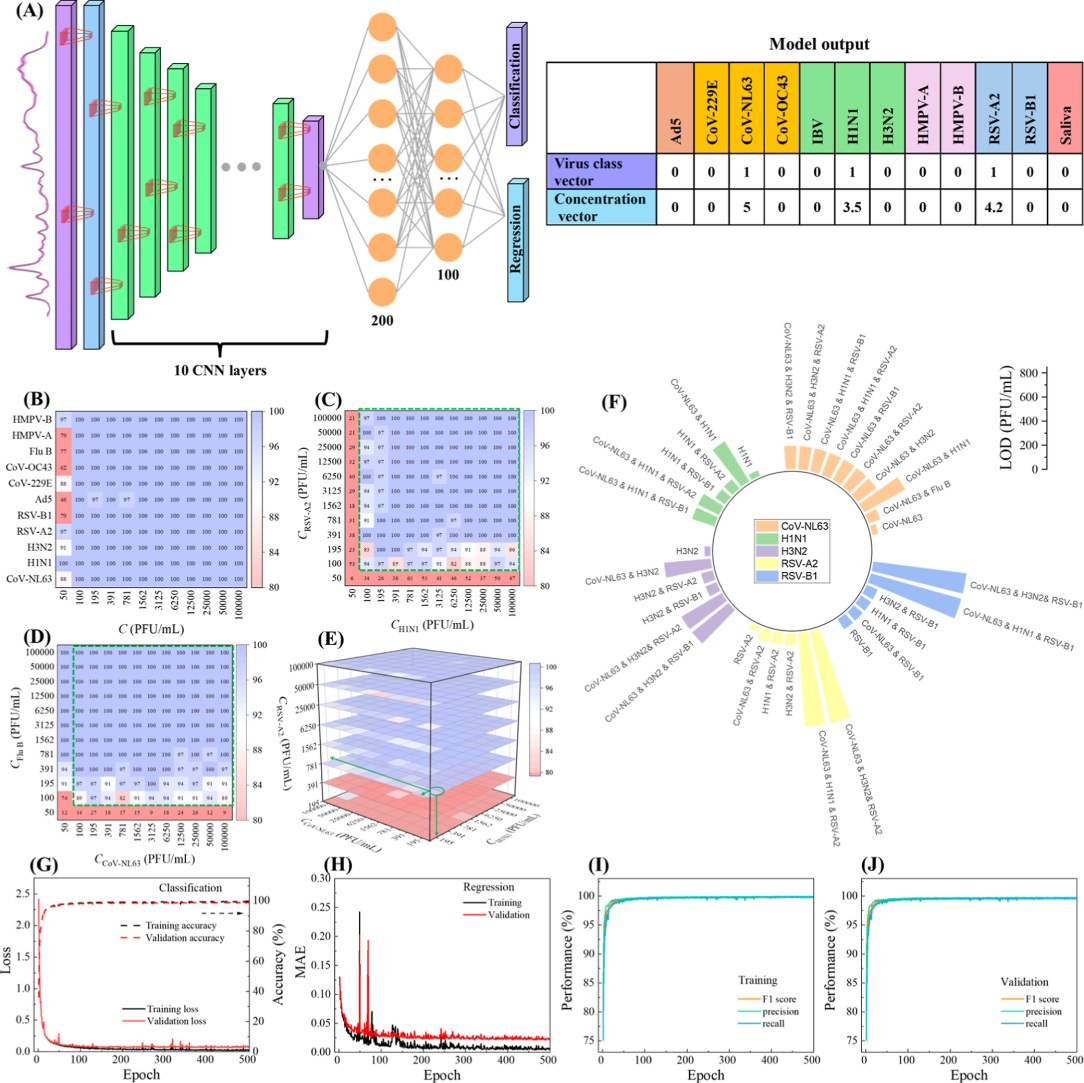
Deep learning model and related results. (A) The architecture of deep learning model “MultiplexCR” and the model output of two 12-element vector labels for multiclass classification and regression of virus mixtures. Detailed classification accuracy heatmaps of different concentration combinations of virus mixtures: (B) 11 SVs with different concentrations, (C) H1N1 & RSV-A2, (D) CoV-NL63 & Flu B, (E) CoV-NL63 & H1N1 & RSV-A2. (F) LODs for different viruses from different virus mixtures. The performance of the MultiplexCR model during the training and validation when increasing the epochs: (G) loss and accuracy for the virus classification, (H) the loss curves for virus concentration regression. Other model performance during the training (I) and the validation (J): F1 score, precision, and recall.

A deep learning model integrated with two tasks to simultaneously solve multiclass classification and regression (concentration prediction) for virus mixtures, called MultiplexCR, is developed as shown in Figure 2A. Figure S12 shows the detailed architecture of MultiplexCR. This model begins with an input layer designed to process SERS spectra from virus mixtures. It incorporates ten sequential convolutional blocks (SCBs), each featuring a 1D convolutional layer with 3 × 1 kernels, ReLU activation, and “same” padding to preserve the input dimensions. These layers are accompanied by batch normalization to stabilize and accelerate the training process. Max pooling layers follow each of the first nine convolutional layers to reduce dimensionality while retaining significant features. The number of convolutional filters starts at 64 and double up to a maximum of 512 through the first seven layers, ensuring progressively deeper feature extraction. The flattened output from these convolutional layers is fed into two fully connected dense layers with 200 and 100 units, respectively, both using ReLU activation to introduce nonlinearity and model complex patterns. The model simultaneously outputs predictions of virus labels and virus concentrations through two distinct dense layers: a sigmoid-activated layer for the classification task and a linear-activated layer for the regression task, each containing 12 elements corresponding to the specified output size. The Adam optimizer is applied with a learning rate of 0.001. The corresponding ground truth labels for the model output are two 12-element vectors, [*V*_1_, *V*_2_, ..., *V*_12_]; [*C*_1_, *C*_2_, ..., *C*_12_]. The element *V*_*i*_ (*V*_*i*_ = 0 or 1) represents the absence or presence of an *i*th virus; and the element *C*_*i*_ indicates the log 10 concentration of *i*th virus. Here, *V*_1_ to *V*_11_, represents Ad5, CoV-229E, CoV-NL63, CoV-OC43, Flu B, H1N1, H3N2, HMPV-A, HMPV-B, RSV-A2, and RSV-B1, respectively, with *V*_12_ being saliva reference. For instance, the vectors [0, 0, 0, 0, 0, 1, 0, 0, 0, 0, 0, 0] & [ 0, 0, 0, 0, 0, 5, 0, 0, 0, 0, 0, 0] indicate a prediction of an SV of H1N1 at a concentration of 10^5^ PFU/mL, and the vectors [0, 0, 1, 0, 0, 1, 0, 0, 0, 1, 0, 0] & [0, 0, 5, 0, 0, 3.5, 0, 0, 0, 4.2, 0, 0] (Figure 2A) represent a virus mixture of CoV-NL63 & H1N1 & RSA-A2 with concentrations of (10^5^, 3162, 15,849) PFU/mL, respectively. A custom loss function is defined for both classification and regression as discussed in Section S9 and Figure S13 in Supporting Information. According to the properties of the MultiplexCR algorithm’s architecture and the output vector, this algorithm can be generalized to predict both viral variant species and concentrations simultaneously for a broader range of viruses.

### Classification and LOD Determination for Virus Coinfection

In the initial training phase, the data set contained the entire SERS spectral data set, covering all concentrations from SVs, 2VMs, and 3VMs, including 3476 kinds of specimens with a total of 1,213,550 SERS spectra. The SERS spectra were randomly divided into training, validation, and testing spectral sets in an 8:1:1 ratio using stratified sampling. The MultiplexCR model was trained with optimized hyperparameters by increasing the number of SCBs from 3 to 10, and the detailed architectures are shown in Figure S14. Adding more CNN layers to the architecture can increase its capacity to learn complex features from the data set, potentially leading to higher accuracy.^40^ As plotted in Figure S15, after 500 epochs of training with different numbers of SCBs, the classification accuracy fluctuated slightly but generally remained between 76% and 82% as the number of SCBs increased from 3 to 7. This may be due to the model’s limited ability to balance feature extraction and overfitting in intermediate architectures. Accuracies continued to rise, reaching approximately 88%, as the number of SCBs increased to 10. This improvement can be attributed to the enhanced capacity of deeper architectures to extract hierarchical and discriminative features from SERS spectra, which are essential for distinguishing subtle spectral differences between virus types. The MAE initially increased, peaking at 5 layers. After 5 layers, the MAE steadily decreased, with the lowest MAE achieved using 10 SCBs. This trend in regression performance aligns with the observed classification performance, where the MAE steadily decreased as the number of SCBs increased, suggesting that deeper architectures not only improved classification but also enhanced quantitative predictions. Considering computational cost and training time, the MultiplexCR architecture with 10 SCBs was selected for virus mixture classification and quantification.

The overall performance for both classification and regression during the training of the MultiplexCR model with 10 SCBs are summarized in Figure S16. The classification loss decreases sharply from 2.53 to 0.51 when the epochs increase from 0 to 50, then stabilizes and reaches 0.35 after 400 epochs for the training spectral set, as shown in Figure S16A. However, the classification loss for the validation spectral set is still around 0.50. Regression loss (MAE) decreases from 0.124 to 0.012 as the epochs increase from 0 to 100, with some fluctuations between 40 and 150 epochs, then stabilizes and reaches 0.007 after 400 epochs for the training spectral set, as shown in Figure S16B. However, the regression loss for the validation spectral set remains around 0.03. The convergence curves for both the training and validation spectral sets indicate that the MultiplexCR model is numerically stable and converges efficiently after 500 epochs. The performance metrics, such as F1 score, precision, recall, are ∼96% for the validation spectral set, as shown in Figure S16D. However, for the testing spectral set, the overall accuracy *A* is 88.4 ± 0.6% for virus mixture classification, and an MAE of 0.034 ± 0.003 for virus concentration regression. The confusion matrix of the MultiplexCR model to classify the virus mixtures is presented in Figure S17. Along the diagonal of the confusion matrix, the *A* values range from 84.6% to 99.8%. The misclassifications range from 0.01% to 9.6%. Among them, the average accuracy for SV classification is 97.38%, whereas for 2VM and 3VM classifications, the *A* = 86.96% and 89.71%, respectively. It is expected that at different virus concentrations, the classification accuracy shall vary, in particular, the accuracy decreases at low concentrations.

Figure 2B shows a heatmap of the classification accuracy of 11 SVs at different virus concentrations. The color scale indicates that purple represents high accuracy (close to 1.0), while red represents lower accuracy. This heatmap reveals that most viruses exhibit very high accuracy (≥97%) across nearly all concentrations. However, at the lowest concentration (*C* = 50 PFU/mL), several viruses exhibit significantly lower accuracy (see red cells). Specifically, Ad5 has the lowest value of *A* = 46%, for CoV-OC43, Flu B, HMPV-A, and RSV-B1, *A* = 62%, 77%, 79%, and 79%, respectively. Other viruses, such as HMPV-B, CoV-229E, RSV-A2, H3N2, and CoV-NL63, have accuracies between 80% and 100%. Notably, H1N1 always has an *A* = 100% regardless of the concentration. Overall, this reduced accuracy at low concentrations of different SVs in Figure 2B could be attributed to two reasons: SNR is small, or the background signal is too high. In classic ways to characterize the performance of a sensor, one usually defines the concentration at SNR = 3 as the LOD (Figure S10). Here, since *A* indirectly reflects how small the virus signal is compared to the noise and background signal, a threshold for the *A* value can be set to determine the LOD. Based on above analysis, we set *A*_th_ = 80%. If this criticism is accepted, according to Figure 2B, the LOD varies among viruses. For instance, the LODs for Ad5 and CoV-OC43 are ∼100 PFU/mL, while for H1N1, the LOD is ∼50 PFU/mL, which is the lowest concentration in our experiment design.

Similar analyses can be carried out for 2VMs and 3VMs. However, since the mixtures involve 2 or more viruses, the analysis becomes more complicated. Figure 2C shows an example accuracy heatmap of H1N1 & RSV-A2, where the *x*-axis and *y*-axis of the map represent *C*_H1N1_ and *C*_RSV-A2_, respectively. Most cells show high accuracy (*A* ≥ 97%), especially at higher concentrations of both viruses. However, the lowest row (*C*_RSV-A2_ = 50 PFU/mL) and the lowest column (*C*_H1N1_ = 50 PFU/mL) exhibit accuracies fluctuating between 6% and 63%. There is a trend that when both *C*_H1N1_ and *C*_RSV-A2_ decrease, *A* value also declines. Applying the LOD criterion discussed earlier, the LOD for H1N1 and RSV-A2 should be 100 PFU/mL. Compared to the LODs in SV detection, the LOD in 2VM for RSV-A2 is the same as the that in SV detection, while for H1N1, the LOD increases from 50 PFU/mL in SV detection to 100 PFU/mL in 2VM detection. This result shows that the addition of a second virus in the specimen can affect the performance of the same sensor platform. As discussed in Figure 1G and S9A–D, the SERS spectra of individual H1N1 and RSV-A2 have unique distinct peaks, so the heatmap Figure 2C shows symmetric patterns along the diagonal line *C*_H1N1_ = *C*_RSV-A2_. Therefore, the LOD for H1N1&RSV-A2 is (100, 100) PFU/mL, and all the specimens in the green dashed box in Figure 2C should be above the LOD and can be detected by the proposed SERS + DLA method. However, the spectra for individual CoV-NL63 and Flu B, exhibit similar spectral shapes. Figure 2D plots the accuracy heat map for CoV-NL63 & Flu B, showing a nonsystemic pattern. The lowest *A* values occur at *C*_Flu B_ = 50 PFU/mL. For *C*_CoV-NL63_ = 50 PFU/mL, though most *A* values at different *C*_Flu B_ are larger than 90%, at *C*_Flu B_ = 100 PFU/mL, *A* = 74%, which is below the threshold 80%. According to the LOD discussion, it is easy to determine that the LOD for Flu B in the 2VM is 100 PFU/mL. Since at (50, 50) and (50, 100) PFU/mL, the *A* values are smaller than 80%, in order to minimize the false-positive prediction, we assume that the *A* values of the entire column at *C*_CoV-NL63_ = 50 PFU/mL are smaller than 80% since there are two values smaller than this threshold, then the LOD for CoV-NL63 is 100 PFU/mL, thus the overall LOD for CoV-NL63 & Flu B is (100, 100) PFU/mL, and the specimens in the dashed green box in Figure 2D are detectable.

For the 3VM case, Figure 2E plots a three-dimensional (3D) accuracy heatmap for CoV-NL63 & H1N1 & RSV-A2. Most of the specimens are purple, indicating high accuracy (*A* ≥ 95%). The highest accuracies are observed when all three viruses are at higher concentrations. However, in the bottom two layers (*C*_RSV-A2_ = 50 and 100 PFU/mL), more red areas are observed, and the *A* values fluctuate between 40% and 70%, regardless of *C*_CoV-NL63_ and *C*_H1N1_. Therefore, the LOD for CoV-NL63 & H1N1 & RSV-A2 is (195, 391, 785) PFU/mL. Figure S18 shows the rest of accuracy heatmaps for other virus mixtures, and Table S5 lists the LODs for different virus mixtures. Figure 2F compares the LODs of viruses that appeared in SV, 2VM, and 3VM detections. Generally, the LODs for SV detection are the lowest compared to those for mixture detection. The LODs of the same virus in 3VM are roughly larger than those in 2VM detection, but there are some exceptions. For example, the LODs for CoV-NL63 in 3VM detection are the same as in 2VM detection. Mixture detection often results in higher LODs due to several factors. First, SNR in mixtures is often significantly lower than in SV detections because the presence of multiple viruses can dilute the signal of each individual virus. SV detections typically exhibit a high SNR due to the strong and distinct signal from a single virus, with minimal spectral overlap and external noise. As complexity increases with 2VMs and 3VMs, the SNR may decrease due to potential overlapping SERS peaks and increased noise from molecular interactions or aggregations. And the different concentration ratios of the viruses in a mixture can influence detection, with dominant viruses potentially overshadowing those present in lower concentrations. Additionally, competing adsorption, where different viruses compete for binding sites on the SERS substrate surface, can further reduce the detection performance. This competition can lead to reduced sensitivity and higher LODs, as the available sites may become saturated or occupied by nontarget molecules in low concentration situations, thereby weakening the overall SERS signal. This variation of LODs indicates the complexity and challenges of accurately detecting multiple viruses simultaneously.

### Final Model Construction and Validation

**Figure 3:**
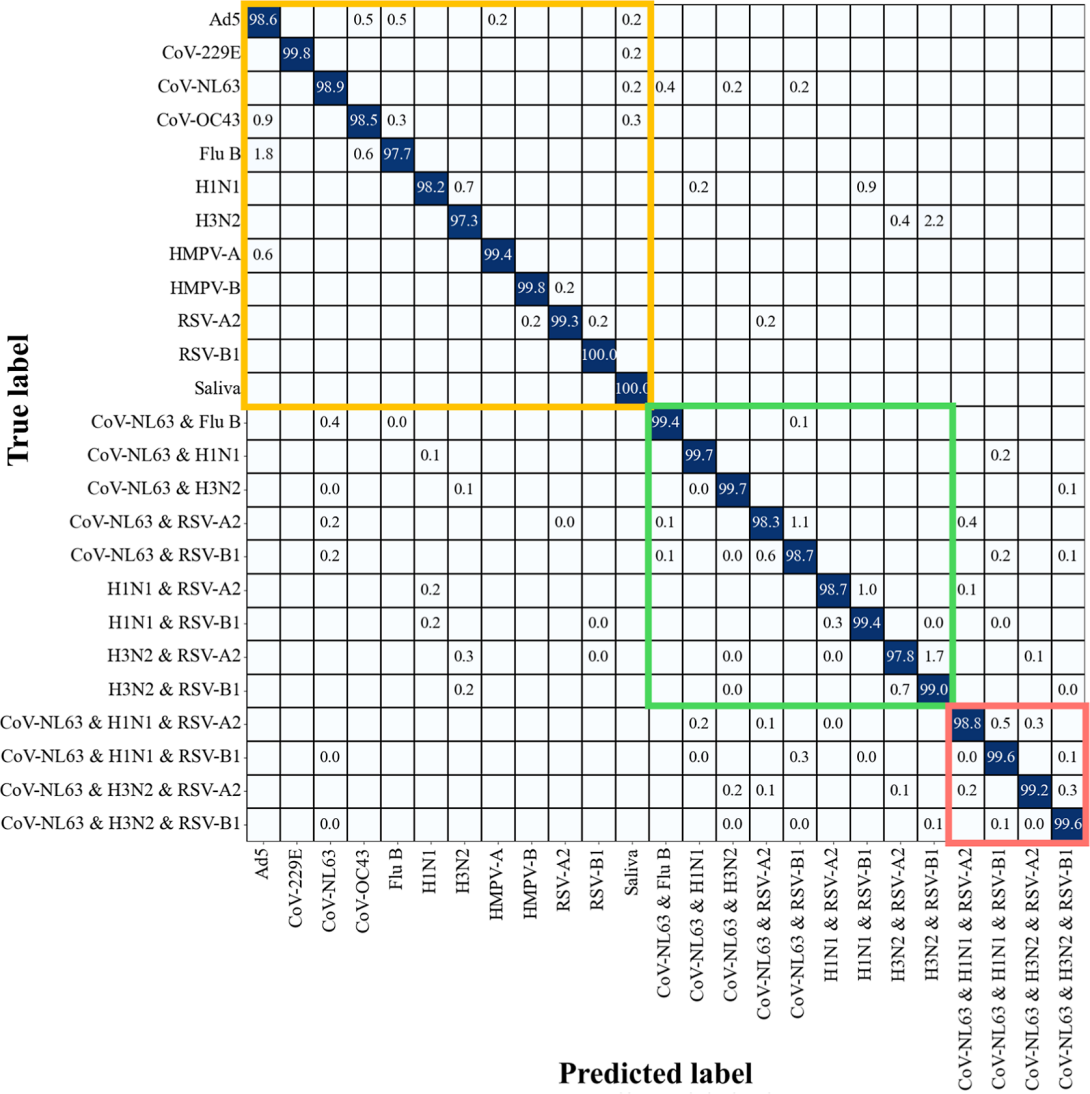
Classification results of the multiplexCR model for virus coinfection detection in saliva. Confusion matrix for 11 SVs, nine 2VMs, and four 3VMs detection in saliva with *C*_virus_ ≥ LODs, as well as reference saliva. The matrix entries represent the percentage of test spectra predicted as a specific class (first row) given a ground truth of class (first column). Diagonal entries show the accuracy for each class. Note that “0.0” indicates values below 0.04%, rather than an actual zero.

Based on the determined LODs, SERS spectra with concentrations above the LOD were retained, while those below were removed. The refined SERS spectral data set contains 2548 types of specimens from SVs, 2VMs, and 3VMs, with a total of 890,530 SERS spectra. The deep learning model was then retrained using this refined data set. Spectra from low concentrations typically introduce more noise and inaccuracies, which can confuse the model and result in poorer generalization and overall performance. The updated performance metrics for both classification and regression are shown in Figure 2G–J. For the classification task, the cross-entropy loss significantly decreased from 2.41 to 0.06 over the first 100 epochs, stabilizing at around 0.03 after 400 epochs with a classification accuracy of 99.7 ± 0.2% for the training spectral set (Figure 2G). The validation spectral set achieved a classification accuracy of 98.7 ± 0.2%, with a final loss of 0.07. In the regression task, the MAE decreased from 0.129 to 0.025 over the initial 30 epochs, showing large fluctuations between epoch 30 and 150. The MAE then stabilized, reaching 0.005 after 400 epochs for the training spectral set (Figure 2H), while the MAE of the validation spectral set is around 0.022. These results indicate that the MultiplexCR model was numerically stable and converged efficiently after 500 epochs. Other performance metrics, such as F1 score, precision, recall, were ∼99.9% for the training spectral set (Figure 2I) and ∼99.5% for the validation spectral set (Figure 2J). For the testing spectral set, the overall accuracy for virus mixture classification was 98.6 ± 0.3%, and the MAE for virus concentration regression was 0.028 ± 0.004. These results show a significant improvement over the original model, which had 88.4 ± 0.6% classification accuracy and an MAE of 0.034 ± 0.003 for virus concentration regression. The updated confusion matrix is shown in Figure 3. The model exhibits outstanding performance in classifying SVs, achieving average accuracies of 98.53%. For virus mixtures, they maintain high accuracy, with average accuracies of 98.97% for 2VMs and 99.29% for 3VMs. Misclassifications ranged from 0.01% to 2.6%. In 2VM cases with distinct peaks, the accuracies range from 97.8% to 99.7%, with a 99.4% accuracy for 2VM cases exhibiting similar spectra. Considering the data set is not balanced across SVs, 2VMs, and 3VMs, the F1 score, precision, and recall were calculated as 98.8 ± 0.6%, 99.1 ± 0.5%, %, 98.7 ± 0.6%, respectively. The corresponding spectral number in the confusion matrix is shown in Figure S19. Additionally, a comparison with Figure S17 illustrates an obvious reduction in misclassifications for both 2VMs and 3VMs in Figure 3, indicating the robustness and effectiveness of the final model in accurately classifying the virus mixtures.

**Figure 4:**
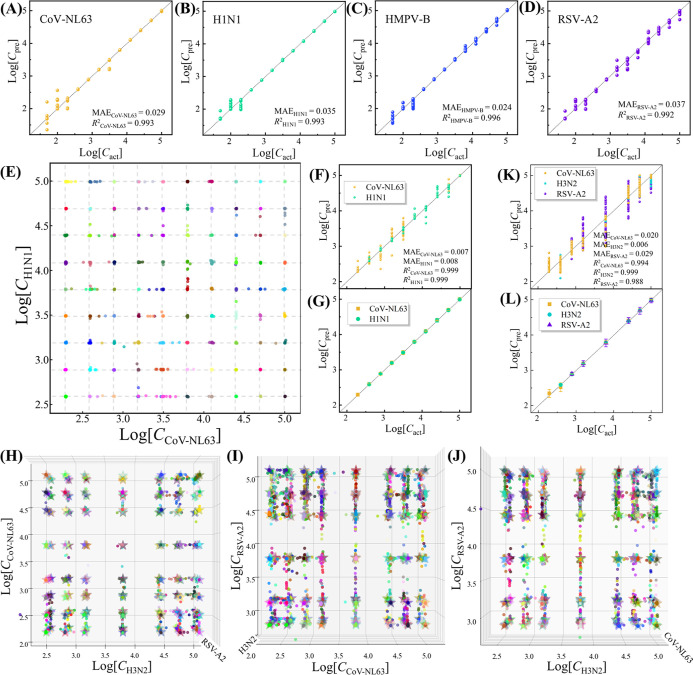
Representative regression results of the MultiplexCR model for virus coinfection detection in saliva. SV detection: (A) CoV-NL63, (B) H1N1, (C) HMPV-B, (D) RSV-A2. The *x*-axis is log_10_*C*_act_ of testing spectra, and *y*-axis is log_10_*C*_pre_. The dash line shows log_10_*C*_act_ = log_10_*C*_pre_. 2VM detection: CoV-NL63 & H1N1: (E) A 2D scattered plot for concentration distributions of CoV-NL63 & H1N1, with *x*-axis is log_10_*C*_CoV-NL63_, and *y*-axis is log_10_*C*_H1N1_. (F) A replot of the regression results from (E) for CoV-NL63 (orange dots) and H1N1 (green dots), *x*-axis is actual log_10_*C*_act_, *y*-axis is the predicted log_10_*C*_pre_, same log_10_*C*_act_ from different specimens are combined. (G) Variations in predicted concentrations based on (F), with the dash line representing log_10_*C*_pre_ = log_10_*C*_act_. 3VM detection: CoV-NL63 & H3N2 & RSV-A2: a 3D scatter plot of predicted concentrations: (H) the top view, (I) front view, (J) side view, with *x*-axis for log_10_[*C*_CoV-NL63_], *y*-axis for log_10_[*C*_H3N2_], and *z*-axis for log_10_[*C*_RSV-A2_]. (K) A replot of the regression results for CoV-NL63, H3N2, and RSV-A2. (L) Variations in predicted concentrations based on (K).

Regression results in logarithms of viral concentration (log_10_ C) from the MultiplexCR model are plotted in Figures 4 and S20–S28. For the detections of 11 SVs in saliva, regression results of the predicted concentration log_10_ (*C*_pre_) and actual concentration log_10_ (*C*_act_) are plotted in Figures 4A–D and S20. The predicted log_10_ C_pre_ and the actual log_10_*C*_act_ all follow the relationship log_10_*C*_pre_ = log_10_*C*_act_ well, with small MAEs ranging from 0.019 to 0.042, and the coefficients of determination *R*^2^ > 0.99, which demonstrates the precision of the regression. For 2VMs, taking CoV-NL63 & H1N1 as an example, Figure 4E shows the 2D scatter plot of predicted logarithmic concentrations of both viruses. Most data points cluster closely around the grids as the experimentally designed virus concentrations, indicating good predictions of both *C*_CoV-NL63_ and *C*_H1N1_ across a wide range of concentrations, which includes cases where one virus is at a high concentration and the other at a low concentration, or when both are at low concentrations. And only a few points deviate from the corresponding grids. To visualize the regression results, Figure 4F plots log_10_*C*_pre_ versus log_10_*C*_act_ for both CoV-NL63 and H1N1, showing that the results closely follow the relationship log_10_*C*_pre_ = log_10_*C*_act_, with small MAEs of 0.002 for CoV-NL63 and 0.008 for H1N1, and *R*^2^ ∼ 0.999, further demonstrating the accuracy of the concentration predictions. And the standard deviations of the log_10_*C*_pre_ are plotted in Figure 4G, with small error bars indicating minimal variations. Figures S21K–L plot the 2D heat maps of average relative errors of predicted *C*_CoV-NL63_ and *C*_H1N1_ across different concentration combinations. Most predictions show the relative errors <5%, only some low-concentration predictions have errors >20%. Overall, the concentration predictions of CoV-NL63 & H1N1 show good performance. The regression results of the other eight 2VMs are plotted in Figures S21–S24, with MAEs ranging from 0.005 to 0.098 and *R*^2^ between 0.967 and 0.999. Most predictions are accurate, except for CoV-NL63 & Flu B with some large variations (Figures S21F–J) due to overlapping spectral signatures that make classifying and quantifying each virus challenging. However, the overall regression results still show a good fit, with *R*^2^ = 0.967 for Flu B and *R*^2^ = 0.997 for CoV-NL63.

Similarly, for 3VMs, taking CoV-NL63 & H3N2 & RSV-A2 as an example, a 3D scatter plot of predicted concentrations can be created to better visualize the regression results, with *x*-axis for log_10_ [*C*_CoV-NL63_], *y*-axis for log_10_ [*C*_H3N2_], and *z*-axis for log_10_ [*C*_RSV-A2_]. Figure 4H–J show the top, front, and side views of this 3D scatter plot. In Figure 4H, predicted concentrations form clusters around the true concentrations, which indicates the accurate concentration predictions of both CoV-NL63 and H3N2. However, in the plots for RSV-A2 versus CoV-NL63 (Figure 4I) and RSV-A2 versus H3N2 (Figure 4J), most clusters are well separated along the *x*-axis, while many clusters show some overlap along the *y*-axis, suggesting higher accuracy for CoV-NL63 and H3N2 predictions compared to RSV-A2. Nonetheless, the averages of each cluster remain close to the true concentrations, indicating accurate prediction regardless of the virus concentrations. In Figure S27, 3D heat maps show that most predictions have an average relative error <5%, with only a few >20%. In Figure 4K–L, scatter and error bar plots on a log–log scale confirm the relationship log_10_*C*_pre_ = log_10_*C*_act_, with small MAEs of 0.020 for CoV-NL63, 0.006 for H3N2 and 0.029 for RSV-A2, and *R*^2^ > 0.985. Figures S25–S28 show the results for other 3VM detections, with MAEs ranging from 0.006 to 0.040 and *R*^2^ between 0.977 and 0.999. Compared to the regression results from the RF model (Section S8), these different visualizations for the results from the MultiplexCR model show the small variations of virus concentration predictions and significantly lower LODs. Overall, the MultiplexCR model shows the good performance of classifications and regression for virus coinfections, with high accuracy and consistency across a wide range of concentration combinations for different viruses.

### Blind Test of Virus Mixture in Saliva

**Figure 5:**
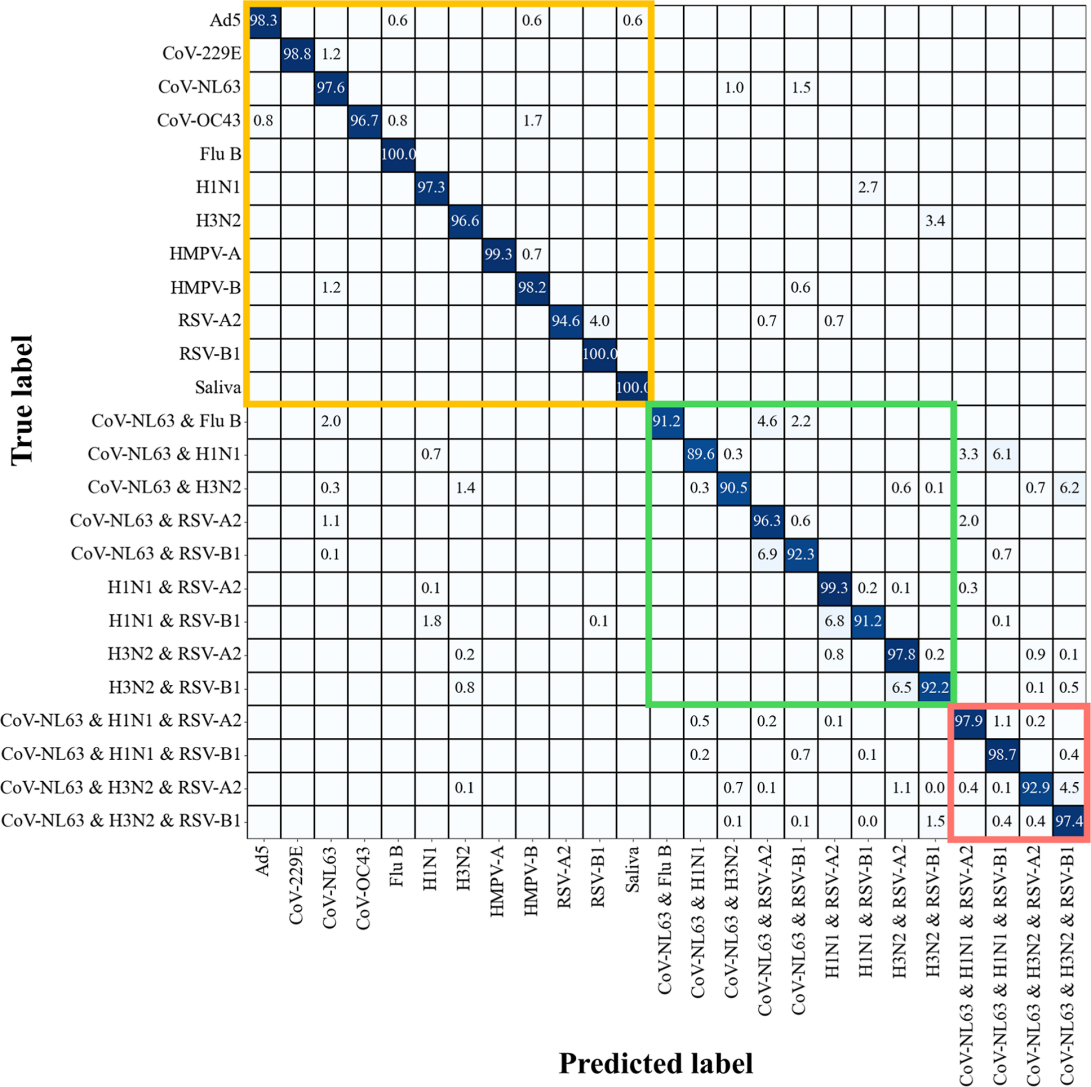
Classification results of the MultiplexCR model for virus coinfection detection in blind tests. Confusion matrix for blind tests including 11 SVs, nine 2VMs, and four 3VMs detection in saliva with *C*_virus_ ≥ LODs, as well as reference saliva. The matrix entries represent the percentage of test spectra predicted as a specific class (first row) given a ground truth of class (first column). Diagonal entries show the accuracy for each class. Note that “0.0” indicates values below 0.04%, rather than an actual zero.

A blind test is crucial for assessing the proposed virus coinfection detection strategy and the performance of the MultiplexCR model on SERS spectra from unknown virus specimens, ensuring its reliability for real-world applications. The blind test involved 734 specimens from 11 SVs, nine 2VMs, and four 3VMs, with all specimens above the corresponding LODs, as listed in Table S6. Random concentrations were designed without reference to the training concentration grids (Figure S1). Approximately 27 spectra were collected for each specimen, resulting in a total of 19,873 spectra, all of which were input into the trained MultiplexCR models for testing. The classification confusion matrix from blind tests (Figure 5) shows an overall accuracy of 95.8 ± 0.6%, with an F1 score of 99.0 ± 0.2%, precision of 99.3 ± 0.2%, and recall of 98.6 ± 0.2%. And the corresponding confusion matrix displaying the number of spectra for the MultiplexCR model’s detection is shown in Figure S29. The model exhibits outstanding performance in classifying SVs, achieving an average accuracy of 98.12%. For virus mixtures, their accuracy remains high, with average accuracies of 93.37% for 2VMs and 96.75% for 3VMs. SVs like Ad5, Flu B, and HMPV-A are predicted with high accuracy, 98.3%, 100%, and 99.3%, respectively. Co-infections like CoV-NL63 & Flu B, CoV-NL63 & RSV-A2, and CoV-NL63 & H1N1 & RSV-A2 are predicted with accuracies of 91.2%, 96.3%, and 97.9%, respectively. The misclassification rate varies modestly, ranging from 0.01% to 6.1%. Overall, the classification results show high accuracy.

**Figure 6:**
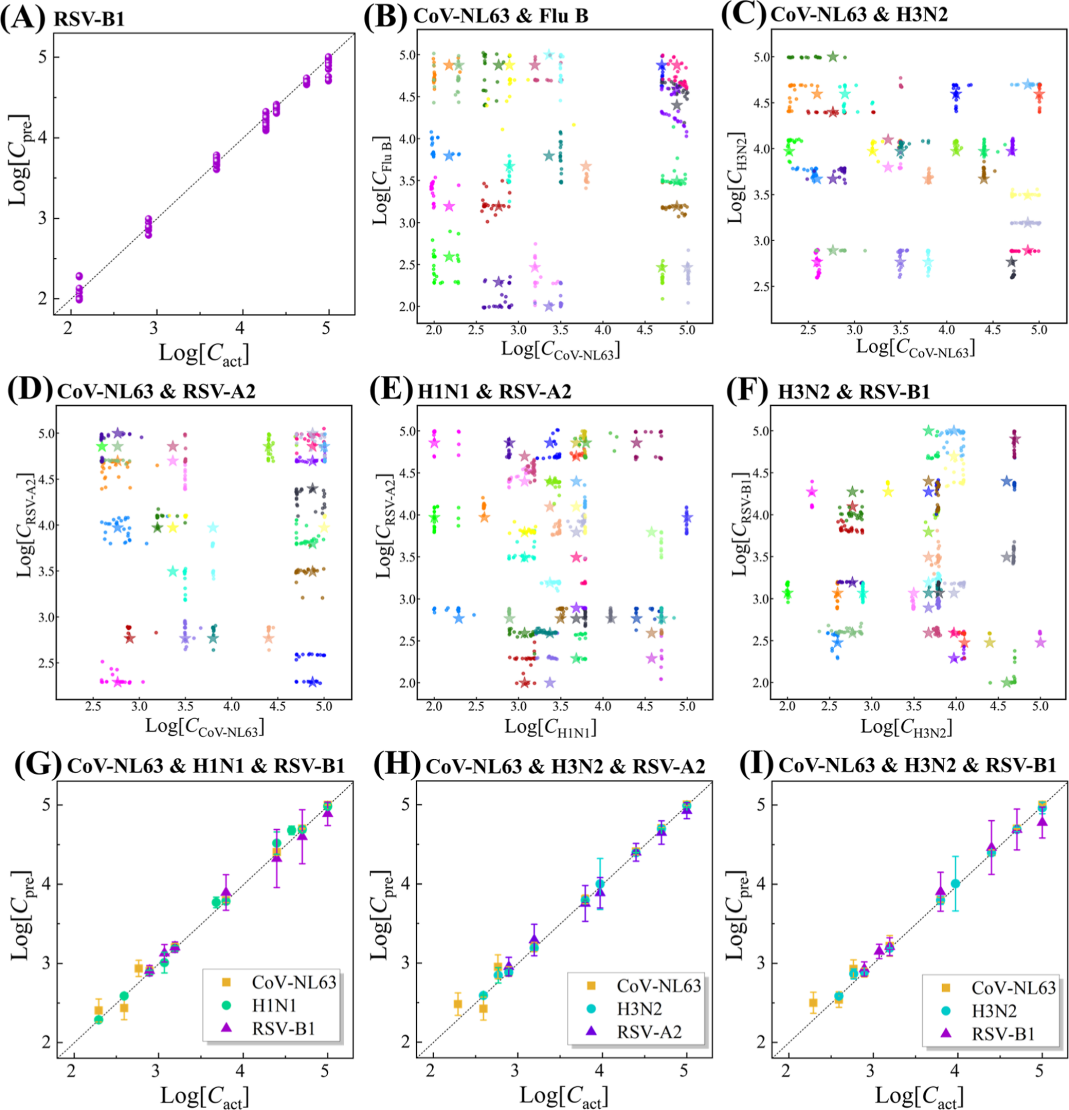
Regression results of the MultiplexCR model in blind tests. (A) RSV-B1, (B) CoV-NL63 & Flu B, (C) CoV-NL63 & H3N2, (D) CoV-NL63 & RSV-A2, (E) H1N1 & RSV-A2, (F) H3N2 & RSV-B1, (G) CoV-NL63 & H1N1 & RSV-B1, (H) CoV-NL63 & H3N2 & RSV-A2, (I) CoV-NL63 & H3N2 and RSV-B1. The *x*-axis is log_10_*C*_act_ of testing spectra, and *y*-axis is log_10_*C*_pre_. The dash line shows log_10_*C*_act_ = log_10_*C*_pre_.

Regarding the regression results for the SV detection in saliva, Figure 6A plots log_10_*C*_pre_–log_10_*C*_act_ for RSV-B1, with other viruses summarized in Figure S30, showing small MAEs ranging from 0.046 to 0.166, and *R*^2^ ranging from 0.932 to 0.994, which demonstrates the accuracy of the regression. For 2VMs, results are shown in Figures 6B–F and S31–S33. In the 2D scatter plot of CoV-NL63 & H3N2 (Figure 6C), most data points overlap or are close to the corresponding stars, indicating good concentration predictions for both *C*_CoV-NL63_ and *C*_H3N2_. The log_10_*C*_pre_ and log_10_*C*_act_ all follow the expected relationship (Figure S31H–I), with MAEs of 0.082 for CoV-NL63 and 0.071 for H3N2, and *R*^2^ of 0.979 and 0.975, respectively. For 3VMs, the regression results (Figures 6G–I and S34) follow the same relationship with MAEs from 0.074 to 0.143 and *R*^2^ from 0.917 to 0.982. Table S7 summarizes the performance of the MultiplexCR model from 5 independent trials across training, validation, test, and blind test spectral data sets. The model achieved high accuracy, with 99.7 ± 0.2% in training, 98.7 ± 0.2% in validation, and 98.6 ± 0.3% in the test set. The blind test showed a slight drop to 95.8 ± 0.6%. Overall, the SERS + MultiplexCR model consistently delivers high classification and quantification performance for virus mixture specimens. For comparison, the final model trained by refined SERS spectral data set shows the better performance as discussed in Section S13. This approach enhances prediction accuracy and overall performance, making the model more reliable and effective for virus coinfection detection in real-world applications. The high classification accuracy (95.8%) and low MAE achieved in the blind test indicate that the current selection provides strong evidence of the MultiplexCR model’s adaptability and potential to generalize to a broader range of coinfection scenarios.

## Conclusions

In summary, this study developed a label-free diagnostic platform combining SERS and deep learning for the rapid and quantitative detection of respiratory virus coinfections. A comprehensive data set of SERS spectra was collected from coinfections of SV, 2VMs, and 3VMs with various concentration combinations. The deep learning model, MultiplexCR, was developed for multitask predictions of virus species and concentrations based on the SERS spectra. Following spectral preprocessing and systematic optimization, the model achieved a classification accuracy of 98.6 ± 0.3% and an MAE of 0.028 ± 0.004 for virus concentration regression. A novel method for determining the LODs using accuracy thresholds was proposed, and detailed LODs for different virus coinfections were provided. In blind tests, the model demonstrated an accuracy of 95.8 ± 0.6% for virus coinfection classification and an MAE of 0.043 ± 0.006 for virus concentration regression, with predicted concentrations closely aligning with actual concentrations. Based on the MultiplexCR model architecture and the characteristics of the output vector, this algorithm can be extended to virus coinfection classification and quantification simultaneously for a broad range of viruses and their variants. Additionally, its scalability facilitates the incorporation of new SERS spectra, ensuring continuous improvement in predictive accuracy. Compared to current commercialized methods, the SERS-MultiplexCR platform demonstrates outstanding performance across key metrics. It achieves impressive classification accuracy and quantitative precision for virus coinfection detection, significantly outperforming PCR (80–95% accuracy) and ELISA (60–85% accuracy). The platform also offers a major advantage in speed, delivering results in minutes, compared to 4–8 h for PCR. Its ability to handle complex coinfections and scale to a broader range of viruses makes it more versatile than multiplex PCR, which is often limited to 5–12 targets. The cost-effectiveness of the SERS-based approach, estimated at $10–$30 per test, offers significant savings over multiplex PCR and NGS, which can range from $25–$100 and up to $5000 per test, respectively. These results demonstrate that SERS-MultiplexCR platform offers clear advantages in speed, sensitivity, accuracy, cost, and scalability, as well as label-free detection, which can serve as a valuable tool for diagnosing complex infectious specimens, enabling comprehensive and real-time monitoring of viral dynamics. This approach has the potential to enhance medical diagnosis, facilitate therapeutic interventions, and is particularly promising for rapid virus coinfection detection and suitability for point-of-care diagnostic platforms. However, testing the platform with real clinical samples remains a key direction for future work. Comparisons to gold-standard methods such as PCR or ELISA, potentially using metrics like receiver operating characteristic (ROC) analysis, would provide further validation of its accuracy and real-world applicability. Furthermore, this approach, based on the adaptable MultiplexCR architecture and its output vector, extends beyond virology, offering robust label-free classification and quantification capabilities for complex mixtures in biosensing, food safety, and environmental monitoring. This versatility reinforces the platform’s potential as a rapid, point-of-care diagnostic tool with broad applications, promising transformative impact in fields requiring high sensitivity and specificity for diverse analytes and coexisting target mixtures.

## Data Availability

The data and code needed to evaluate the conclusions in the paper are present in the paper and/or the Supporting Information and can be found at https://github.com/jimcui3/Virus_Co-infection. Additional data related to this paper may be requested from the authors.

## Supporting Information Available

Detection methods for virus coinfection, additional descriptions for the experiments, SERS substrate characterization, data preprocessing, summary of SERS peak assignment, additional results of SERS spectral understanding, traditional calibration curves, performance of traditional machine learning models, optimization of deep learning models, determination of limit of detection (LOD) for virus coinfection, additional results for quantification of virus coinfections, additional information and results for blind tests, the reason for retraining the model (PDF)
