# Supplementary material for: Multiplex Detection and Quantification of Virus Co-Infections Using Label-free Surface-Enhanced Raman Spectroscopy and Deep Learning Algorithms
Source: ACS Sens. 2025 Jan 28;10(2):1298–311. doi: 10.1021/acssensors.4c03209 (PMC11877629; doi:10.1021/acssensors.4c03209)
Supplement: Supplementary file 1 — se4c03209_si_001.pdf [file se4c03209_si_001.pdf]

Supporting Information

Multiplex Detection and Quantification of Virus Co-infections using Label-free Surface-Enhanced Raman Spectroscopy and Deep Learning Algorithms

Yanjun Yang [1] *, Jiaheng Cui [2], Amit Kumar [1], Dan Luo [3], Jackelyn Murray [4], Les Jones [4], Xianyan Chen [5], Sebastian Hülck [6], Ralph A. Tripp [4], Yiping Zhao [1] *

[1] Department of Physics and Astronomy, Franklin College of Arts and Sciences, The University of Georgia, Athens, Georgia 30602, United States.

[2] School of Electrical and Computer Engineering, College of Engineering, The University of Georgia, Athens, Georgia 30602, United States.

[3] Department of Statistics, Franklin College of Arts and Sciences, The University of Georgia, Athens, Georgia 30602, United States.

[4] Department of Infectious Diseases, College of Veterinary Medicine, The University of Georgia, Athens, Georgia 30602, United States.

[5] Department of Epidemiology & Biostatistics, College of Public Health, The University of Georgia, Athens, GA 30602, United States.

[6] Tec5USA Inc., Plainview, NY 11803, United States.

* Corresponding Author: E-mail: YanjunYang@uga.edu; zhaoy@uga.edu

Contents

Section S1. Detection methods for virus co-infection. 3

Section S2. Additional descriptions for the experiments. 6

Section S3. SERS substrate characterization. 10

Section S4. Data pre-processing. 12

Section S5. Summary of SERS peak assignment. 12

Section S6. Additional results of SERS spectral understanding. 17

Section S7. Traditional calibration curves. 24

Section S8. Performance of traditional machine learning models. 26

Section S9. Optimization of deep learning models. 30

Section S10. Determination of limit of detection (LOD) for virus co-infection. 36

Section S11. Additional results for quantification of virus co-infections. 40

Section S12. Additional information and results for blind tests. 49

Section S13. The reason for re-training the model. 61

References 64

Section S1. Detection methods for virus co-infection.

Table S1. Summary of various detection methods for virus co-infection.

Detection methods	Principle	Pros	Cons	Typical applications	Time to results	Cost	Ref
**Polymerase chain reaction (PCR)**	Amplification of viral DNA/RNA	• High sensitivity and specificity<br>• Can detect multiple viruses simultaneously<br>• Quantitative (qPCR) options available	• Requires specialized equipment and expertise<br>• Expensive<br>• Time-consuming<br>• Labeled detection	• Diagnostic labs<br>• Research<br>• Clinical diagnostics	3-6 hours	$10,000 - $50,000 for equipment; $5 - $20 per test	[1]
**Multiplex PCR**	Simultaneous amplification of multiple targets	• Detects multiple viruses in a single reaction<br>• Convenient when testing for multiple pathogens	• Potential for primer-primer interactions<br>• Complexity in primer design<br>• Labeled detection	• Diagnostic labs<br>• Clinical diagnostics	4-8 hours	$20,000 - $100,000 for equipment; $25 - $100 per test	[2-4]
**Next-generation sequencing (NGS)**	High-throughput sequencing of viral genomes	• Comprehensive detection<br>• Can identify novel and multiple viruses<br>• High throughput	• Expensive<br>• Requires advanced bioinformatics<br>• Longer turnaround time	• Research<br>• Epidemiology<br>• Diagnostic labs	Several days to weeks	$200,000 - $1,000,000 for equipment; $500 - $2,000 per sample	[5, 6]
**Metagenomic sequencing**	Sequencing of all genetic material in a sample	• Detects all viral sequences in a sample<br>• Can discover new or unexpected viruses	• Expensive<br>• Requires advanced bioinformatics<br>• Complex interpretation of results	• Research<br>• Discovery of new pathogens<br>• Comprehensive diagnostics	Several days to weeks	$200,000 - $1,000,000 for equipment; $1,000 - $5,000 per sample	[7]
**Enzyme-linked immunosorbent assay (ELISA)**	Antibody-antigen interaction detection	• Relatively simple and quick<br>• Cost-effective for large-scale screening	• Lower sensitivity and specificity compared to molecular methods<br>• Limited to known viruses<br>• Labeled detection	• Diagnostic labs<br>• Epidemiology<br>• Clinical diagnostics	2-5 hours	$10,000 - $50,000 for equipment; $10 - $15 per test	[8, 9]

**Rapid antigen tests, Lateral flow assays (LFAs)**	Antibody-antigen interaction on test strips	• Quick results<br>• Easy to use<br>• Inexpensive	• Lower sensitivity, especially for co-infections<br>• Limited to known viruses<br>• High false-negative rates	• Point-of-care<br>• Field diagnostics<br>• Initial screening	15-30 minutes	$1 - $10 per test (no equipment needed)	[10, 11]
**Serological assays**	Detection of antibodies against viruses	• Can detect past infections<br>• Useful for epidemiological studies	• Cannot distinguish between active and past infections<br>• Lower sensitivity for early detection	• Epidemiology<br>• Clinical diagnostics<br>• Research	1-2 days	$10,000 - $50,000 for equipment; $5 - $20 per test	[12]
**Immunofluorescence assay (IFA)**	Fluorescently-labeled antibodies for virus detection	• Can visualize virus location<br>• Useful for tissue samples	• Lower throughput<br>• Requires fluorescent microscopy<br>• Less quantitative	• Research<br>• Pathology<br>• Diagnostic labs	1-2 days	$20,000 - $100,000 for equipment; $5 - $20 per test	[13]
**Flow cytometry**	Detection of viral particles or infected cells by labeling	• High throughput<br>• Can analyze a large number of cells rapidly	• Expensive<br>• Requires skilled personnel<br>• Complex data analysis	• Research<br>• Diagnostic labs<br>• Vaccine development	2-8 hours	$50,000 - $500,000 for equipment; $5 - $30 per test	[14]
**LAMP (Loop-Mediated Isothermal Amplification)**	Isothermal DNA amplification	• Fast<br>• Not require thermocyclers<br>• Easy to use in the field	• Less specificity compared to PCR<br>• Optimization required for each virus	• Diagnostic labs<br>• Research<br>• Clinical diagnostics	30-60 minutes	$5,000 - $30,000 for equipment; $2 - $10 per test	[15]
**CRISPR*-based Diagnostics**	CRISPR-Cas system to detect specific viral RNA/DNA	• High specificity<br>• Can be combined with other techniques for rapid detection	• Still in early stages for co-infection detection<br>• Requires optimization	• Diagnostic labs<br>• Research<br>• Clinical diagnostics	1-2 hours	$10,000 - $100,000 for equipment; $20 - $50 per test	[16]
**Microarray**	Hybridization of nucleic acids to a chip	• Can screen for a large number of viruses<br>• High throughput	• Lower sensitivity compared to PCR<br>• Expensive setup<br>• Requires specialized equipment	• Research<br>• Epidemiology<br>• Large-scale screening	1-2 days	$50,000 - $200,000 for equipment; $50 - $150 per test	[17, 18]

**Viral culture**	Growth of viruses in cell culture	• Can isolate live virus<br>• Allows for further phenotypic characterization<br>• Can be used for a wide range of viruses	• Time-consuming<br>• Requires biosafety level containment<br>• Not suitable for all viruses<br>• Skilled personnel needed	• Research<br>• Diagnostic labs<br>• Vaccine development	Several days to weeks	$20,000 - $200,000 for equipment; $50 - $500 per sample	[19, 20]

***CRISPR**: short for "clustered regularly interspaced short palindromic repeats", is a technology that research scientists use to selectively modify the DNA of living organisms.

Section S2. Additional descriptions for the experiments.

Materials.

Silver (99.999%, Kurt J. Lesker, Jefferson Hills, PA, USA) and titanium pellets (99.995%, Kurt J. Lesker, Jefferson Hills, PA, USA) were purchased as evaporation materials. Tetraethylorthosilicate (TEOS; 99.9%, Alfa Aesar, Ward Hill, MA, USA), ammonium hydroxide (28.0 - 30.0 wt.%, J. T. Baker, Phillipsburg, NJ, USA) and ethanol (EtOH; 95%, Sigma-Aldrich, St. Louis, MO, USA) were used for silica coating on AgNRs. Polydimethylsiloxane (PDMS; Sylgard 184 silicone elastomer kit) was purchased from Dow Corning (Midland, MI, USA). Deionized water with a resistivity of 18.2 MΩ·cm was used throughout all the experiments. All the reagents were used without further purification.

AgNR@SiO_2 array SERS substrate fabrication.

AgNR@SiO_2 array SERS substrates were prepared by the oblique angle deposition (OAD) and salinization via hydrolysis of TEOS as described previously [21, 22]. OAD is chosen for SERS substrate fabrication due to its ability to create uniform, reproducible nanorod structures, which enhance SERS sensitivity. Additionally, OAD is cost-effective, scalable, and suitable for high-throughput production, making it ideal for reliable and affordable diagnostic applications. The AgNR substrates were first prepared using OAD[23, 24]. Piranha solution (a 3:1 mixture of sulfuric acid to hydrogen peroxide) cleaned glass slides (0.5 inch × 0.5 inch) were mounted in a custom-designed electron beam deposition system. A layer of 20 nm Ti film and a layer of 100 nm Ag film were subsequently deposited at a rate of 0.2 nm/s and 0.3 nm/s, respectively. Then, the vapor incident angle was adjusted to be 86°, and a thickness of 2000 nm Ag film was deposited at a rate of 0.3 nm/s to form the AgNRs on the substrates. The entire evaporation process was conducted under a high vacuum condition (chamber pressure $< 3 \times 10^{-6}$ Torr). After the deposition, the AgNR substrates were immersed into a homogeneous mixture of 30 mL of EtOH, 4 mL of H_2O, and 500 μL of TEOS for 20 min under stirring. The coating of SiO_2 was initiated after adding 560 μL of ammonium hydroxide. The substrates were removed from the reaction solution after 5 min, followed by water rinsing and N_2 drying. A 2-nm conformal SiO_2 coating on AgNR was expected under such conditions. This SiO_2 layer is thin enough to preserve the strong plasmonic enhancement while ensuring substrate stability. The SERS enhancement factor remained sufficiently high for sensitive detection of virus specimens. Subsequently, arrayed small wells (4

wells, with a well diameter of 4 mm and a well depth of 1 mm) on a PDMS layer were molded on the AgNR@SiO_2 array to restrict the effective sensing areas [25], and we refer them as AgNR@SiO_2 wells.

Virus incubation.

All viruses were propagated in Vero E6 cells, which were maintained in Dulbecco's Modified Eagle Medium (DMEM; GIBCO BRL laboratories, Grand Island, NY) supplemented with 1% fetal bovine serum (FBS; Hyclone Laboratories, Salt Lake City, UT). Briefly, cells were infected using a multiplicity of infection (MOI) = 0.1. After 48 h, the viruses were harvested in serum-free DMEM, followed by freeze-thawing after which the contents were collected and centrifuged at 4,000 g for 15 min at 4°C. The virus titers were similar, i.e., 10^6 PFU/mL, as determined by plaque assay as previously described [26-28]. The reference specimen for these studies was diluted in DMEM supplemented with 1% FBS. Influenza strains, H1N1 and H3N2, were propagated in embryonated chicken eggs and virus titers determined by hemagglutination assay using chicken red blood cells. The influenza virus titers ranged between 10^7 - 10^8 50% egg infectious dose (EID_{50}). The reference specimen for these studies was naïve allantoic fluid. All the experiments were performed in a BSL-2 lab, and all experimental operations followed the biosafety guidelines provided by the CDC: https://www.cdc.gov/coronavirus/2019-nCoV/lab/lab-biosafety-guidelines.html.

The following precautions were taken to ensure the safe handling of all virus specimens used in this study. All experimental work involving live viruses was conducted in a Biosafety Level 2 (BSL-2) laboratory, which is appropriate for the viruses used in this study. The viruses included in the study, such as coronaviruses, influenza strains, respiratory syncytial viruses, and human metapneumoviruses, are classified as BSL-2 pathogens based on standard biosafety guidelines. None of the viruses used in this study required BSL-3 containment based on their risk group classification. The study adhered to institutional and national biosafety regulations for working with BSL-2 pathogens. The following safety measures were strictly followed: (1) Personal protective equipment (PPE): Laboratory personnel wore appropriate PPE, including lab coats, gloves, N95 respirators, and eye protection, to minimize exposure risks. (2) Biosafety cabinets (BSC): All procedures involving virus handling, including preparation of virus-spiked samples, were performed in the biosafety cabinets to prevent aerosol exposure and contamination. (3)

Decontamination: Work surfaces and equipment were regularly decontaminated using 70% ethanol and appropriate virucidal agents. All waste materials, including virus-containing samples and consumables, were autoclaved before disposal. (4) Training: Laboratory personnel conducting these experiments were properly trained in BSL-2 biosafety procedures and protocols.

Design of virus mixture specimens.

The SERS spectra were collected from 11 respiratory virus species, 9 two-virus mixtures (2VMs), and 4 three-virus mixtures (3VMs). **Table S2** provides a summary of the virus mixtures, including the types of viruses. For 2VMs and 2VMs, the relative and absolute concentrations of each virus in the mixture were designed according to **Figure S1.**

Table S2. Information about virus mixtures.

Category	Virus species
**Single viruses**	Ad5
	CoV-229E
	CoV-NL63
	CoV-OC43
	Flu B
	H1N1
	H3N2
	HMPV-A
	HMPV-B
	RSV-A2
	RSV-B1
**Two-virus mixtures**	CoV-NL63 & Flu B
	CoV-NL63 & H1N1
	CoV-NL63 & H3N2
	CoV-NL63 & RSVA2
	CoV-NL63 & RSVB1
	H1N1 & RSV-A2
	H1N1 & RSV-B1
	H3N2 & RSV-A2
	H3N2 & RSV-B1
**Three-virus mixtures**	CoV-NL63 & H1N1 & RSV-A2
	CoV-NL63 & H1N1 & RSV-B1
	CoV-NL63 & H3N2 & RSV-A2
	CoV-NL63 & H3N2 & RSV-B1

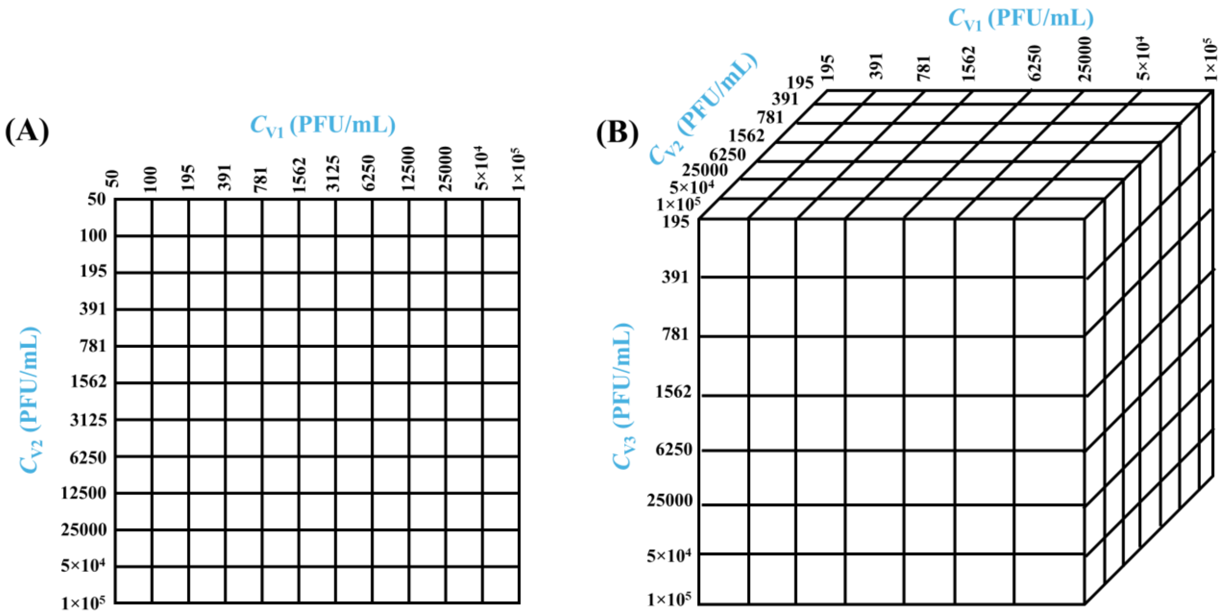

Figure S1. Virus mixture preparation with different concentration combinations. (**A**) two-virus mixture (2VM), and (**B**) three-virus mixture (3VM).

Section S3. SERS substrate characterization.

Using a highly sensitive, reproducible, and uniform SERS substrate for measurement is essential for obtaining high signal-to-noise ratio (SNR) spectra with good reproducibility, which is necessary to achieve high identification accuracies. The AgNR array has been shown to possess good SERS reproducibility (~10% relative variation), high SERS enhancement factor (up to 10^9), and considerable uniformity[23, 24, 29-31]. To overcome issues of surface contamination and improve long-term stability, reliability, and biocompatibility, the AgNR arrays were coated with a uniform and thin silica layer by the hydrolysis of tetraethyl orthosilicate to form AgNR-SiO_2 core-shell arrays which can serve as an ideal SERS substrate for virus mixture detection. **Figure 1B** shows a representative SEM image of AgNR@SiO_2 array. The surface is composed of tilted nanorods with a wide range of morphologies, such as corrugations, bifurcations, and protrusions. The nanorod density is 12 ± 1 rods/μm^2, the average diameter is 100 nm, and the length is approximated as ~1000 nm with a tilted angle 77 ± 1°. To analyze the components in the surface coating of SiO_2 on the AgNR substrate, XPS, EDS and FTIR characterizations were carried out and results can be found our recent publication[32]. The plasmonic property of the substrates can be characterized by measuring the UV-Vis reflection spectra since the AgNR array was grown on a 100 nm-Ag film. **Figure 1C** shows a typical reflection spectrum of the substrate, the reflection increases monotonically with wavelength λ from 320 nm to 800 nm, showing a broad spectra response. At $\lambda = 785$ nm, the reflection is 73.9%.

SERS characterization was performed by drop-casting a 1 μL droplet of the Raman probe molecule, trans-1,2-bis(4-pyridyl)ethylene (BPE, Aldrich, 99.9+%), dissolved in methanol at a concentration of 10^{-5} M, onto the SERS substrates. The SERS spectra were collected using an excitation wavelength of $\lambda_{ex} = 785$ nm. A typical SERS spectrum is shown in **Figure 1D**. Based on our previous studies, the SERS enhancement mechanism is mainly due to the hot spot between the nanorods as well as on the tip of the nanorods [33]. The wetting induced bundling effect can also play a role to further enhance the SERS signal [34-38]. In addition, the random morphology and roughness of the nanorods could produce additional SERS "hot spot" locations. The uniformity of the AgNR@SiO_2 substrate was characterized by mapping the BPE peak intensity ($\Delta v = 1200$ cm^{-1}) as shown in **Figure S2B**. The mapping shows a uniform distribution of BPE intensity, with an intensity variation of 7%. And the bath-to-batch variation of AgNR@SiO_2 is ~10% as shown in **Figure S2C**, showing a good uniformity of SERS substrates.

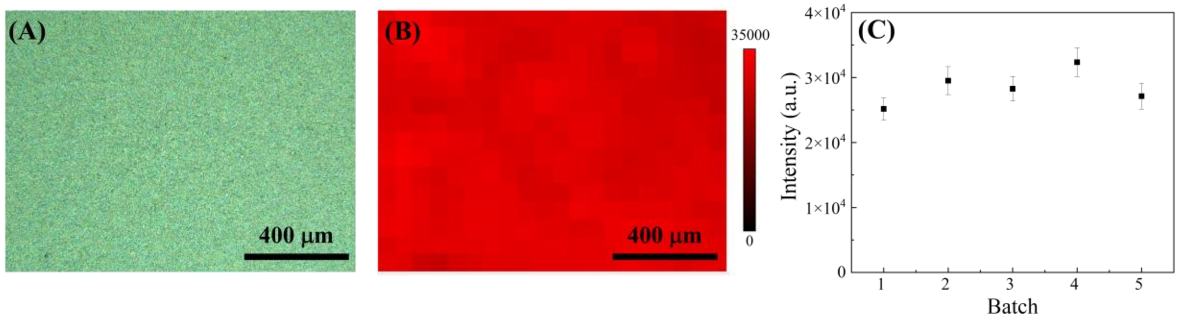

Figure S2. Characterization of SERS substrates. (**A**) A representative optical micrograph of an AgNR@SiO_2 substrate and (**B**) the corresponding SERS intensity mapping of $\Delta\nu = 1200$ cm^{-1} peak of 1×10^{-5} M BPE on the substrate. (**C**) The average SERS intensities and variations at $\Delta\nu =$ 1200 cm^{-1} of 1×10^{-5} M BPE on five batches of AgNR@SiO_2 substrates.

Section S4. Data pre-processing.

Considering the variation in SERS spectra, a typical spectra preprocessing procedure is implemented, which includes baseline correction using the Gaussian-Lorentzian function method[39] and airPLS.[40] In addition, normalization by the average value is applied to each spectrum for further analysis. Such a process guarantees a minimum disturbance for the raw data and avoids non-necessary information loss due to spectral pre-processing.

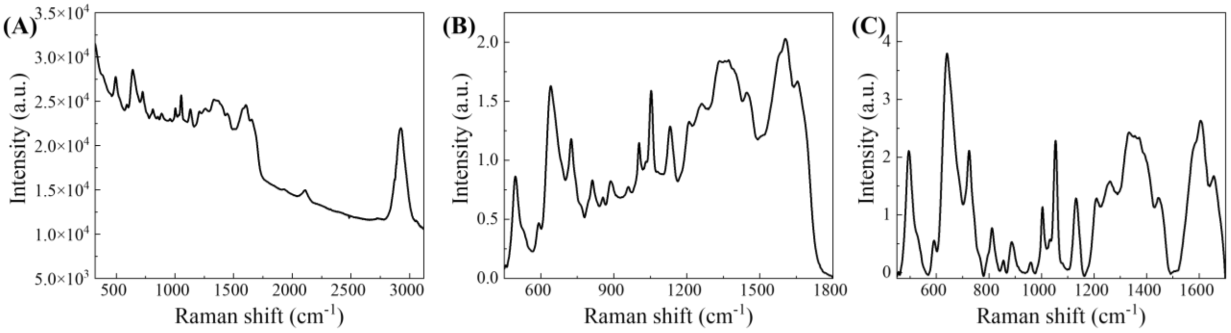

Figure S3. Spectrum pre-process strategy. (**A**) A representative raw spectrum of CoV-NL63 & RSV-A2. (**B**) Baseline corrected and normalized (by the average spectral value) spectrum using the Gaussian-Lorentzian function method. (**C**) Baseline-corrected and normalized spectrum using the airPLS method.

Section S5. Summary of SERS peak assignment.

The most prominent spectral features of the Ad5 SERS spectrum are at $\Delta\nu$ = 518 cm^{-1} corresponding to disulfide stretching mode. The SERS peaks at $\Delta\nu$ = 960 cm^{-1} arise from tryptophan (Trp) and valine (Val). The SERS peaks at $\Delta\nu$ = 1003 cm^{-1} and 1031 cm^{-1} arise from phenylalanine. The SERS peak at $\Delta\nu$ = 1609 cm^{-1} corresponds to tyrosine (Tyr), Trp, and phenylalanine (Phe).

For Influenza B SERS spectra, the most prominent spectral features observed in the spectra are at $\Delta\nu$ = 659 cm^{-1}, 727 cm^{-1}, and 1330 cm^{-1} corresponding to vibrational modes of guanine, the adenine ring vibration, and adenine respectively. The SERS peaks at $\Delta\nu$ = 1003 cm^{-1} and 1034 cm^{-1} arise from phenylalanine, and the SERS peak at $\Delta\nu$ = 1578 cm^{-1} can be attributed to carbonyl groups on the amino acid side chains.

For HMPV SERS spectra, the most prominent spectral features are observed at $\Delta\nu = 658\ \mathrm{cm}^{-1}$ corresponding to a vibrational mode of guanine. The SERS peaks at $\Delta\nu$ = 1004 cm^{-1} and 1034 cm^{-1} arise from phenylalanine. The SERS peaks between $\Delta\nu$ =1294 cm^{-1}, 1580 cm^{-1}, and 1700 cm^{-1} can be attributed to vibrations of amide III, carbonyl groups on the amino acid side chains, and the amide I vibration. There are only a few subtle differences between the SERS spectra of HMPV-A and HMPV-B: HMPV-B shows a higher peak at $\Delta\nu$ = 738 cm^{-1} corresponding to Trp, while HMPV-A shows a more obvious peak at $\Delta\nu$ = 833 cm^{-1} corresponding to Trp and $\Delta\nu$ =908 cm^{-1} corresponding to nucleic acid skeleton vibration[41].

For RSV SERS spectra, the main peak at $\Delta\nu$ = 1053 cm^{-1} can be assigned to the C-N stretching vibration. RSV has spike-like glycoprotein projections on its membrane envelope comprising two major glycoproteins, i.e., F and G glycoproteins. The peak at $\Delta\nu$ = 1456 cm^{-1} can be assigned to the CH_2 deformation vibration arising from the proteins or the lipids in the membrane. Compared to RSV-A2, RSV-B1 has additional peaks at $\Delta\nu$ = 1456 cm^{-1} corresponding to histidine and $\Delta\nu$ = 858 cm^{-1} corresponding to Tyr (β-sheet), likely attributed to the envelope.

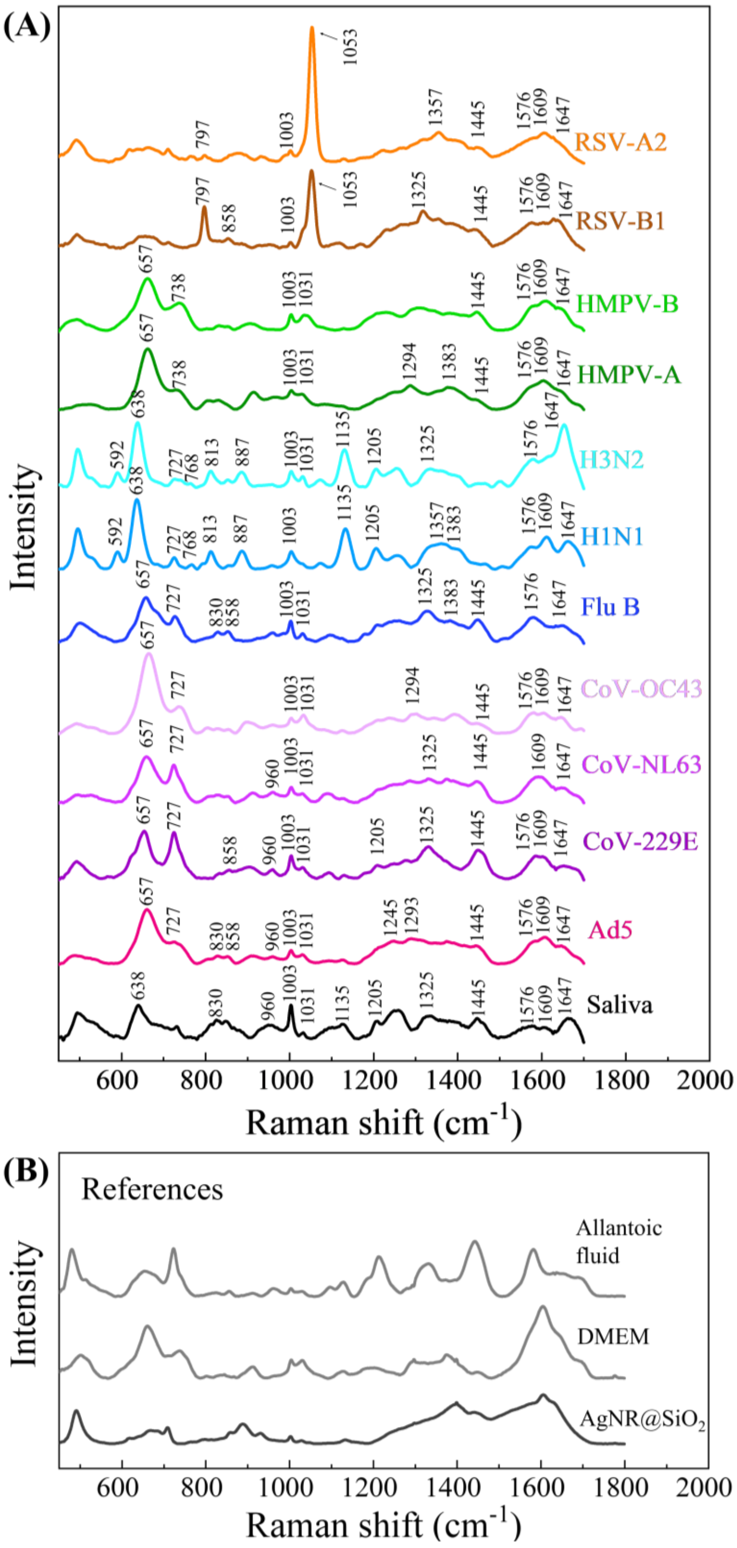

Figure S4. (**A**) Average SERS spectra with labeled peaks from 11 viruses at 10^5 PFU/mL and the saliva specimen. (**B**) Average SERS spectra of three references: AgNR@SiO_2 substrate, DMEM buffer, and allantoic fluid.

The stability of virus-saliva specimens (H1N1 in saliva with concentration of 50000 PFU/mL) during SERS measurements was assessed by conducting time-dependent spectral measurements at defined intervals (0, 10, 30, 60, and 120 min after sample preparation). The spectral features showed negligible variation over time, with high reproducibility confirmed through correlation coefficients results ranging from 0.996 to 0.999. These results confirm the stability of the virus-saliva specimens and the reliability of the SERS measurements.

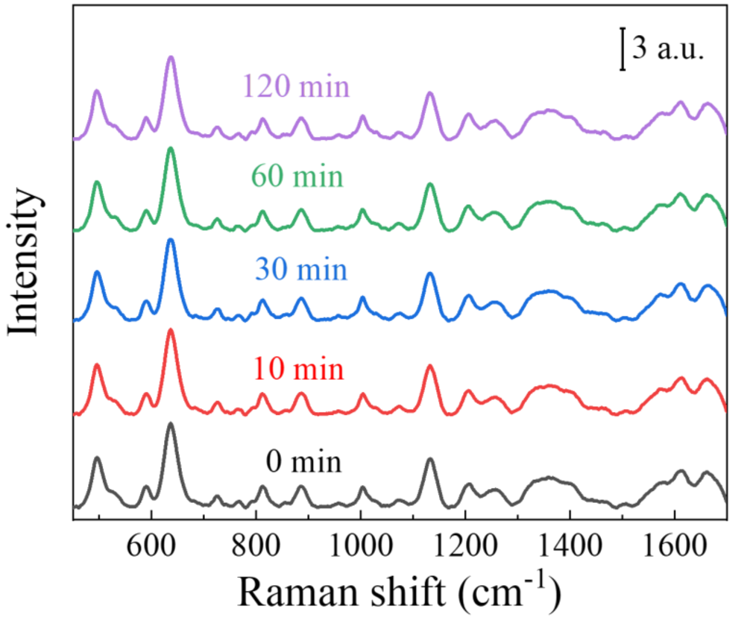

Figure S5. Stability assessment of H1N1 virus-saliva specimens (50000 PFU/mL) during SERS measurements. Time-dependent spectral measurements were conducted at intervals of 0, 10, 30, 60, and 120 min after sample preparation.

Table S3. SERS peak assignment summary of 11 viruses and saliva.[32, 42, 43]

**Peak (cm^{-1})**	**Assignment**	**Ad5**	**CoV-229E**	**CoV-NL63**	**CoV-OC43**	**Influenza B**	**Influenza A (H1N1)**	**Influenza A (H3N2)**	**HMPV-A**	**HMPV-B**	**RSV-B1**	**RSV-A2**	**Saliva**
592	Gly						√	√					
638	Tyr (α-sheet)						√	√					√
657	Guanine/Tyr	√	√	√	√	√			√	√			
727	adenine	√	√	√	√	√	√	√					
738	Trp								√	√			
768	Trp						√	√					
797	Histidine										√	√	
813	phosphate backbone stretch of the RNA						√	√					
830	Tyr	√				√							√
858	Tyr (β-sheet)	√	√			√					√		
887	Gly						√	√					
960	Trp, Val	√	√	√		√							
1003	the symmetric ring breathing mode of Phe	√	√	√	√	√	√	√	√	√	√	√	√
1031	the in-plane C-H bending mode of Phe	√	√	√	√	√		√	√	√			√
1053	the C-N stretching vibration										√	√	
1135	*v*(C-C), C-N	√					√	√					√
1205	Tyr		√				√	√					√
1237	thymine												
1245	guanine	√											
1294	amide III	√			√				√				
1325	adenine		√	√		√		√			√		√
1357	Trp, C_a-H						√					√	
1383	$\delta(CH_3)$, v (C-N)					√	√		√				
1445	CH_2 deformation vibration	√	√	√	√	√			√	√	√	√	√
1576	the carboxylate stretching vibration ($\nu_a COO^-$) of Trp	√	√		√	√	√	√	√	√	√	√	√
1609	Tyr, Trp, Phe	√	√	√	√		√		√	√	√	√	√
1647	the amide I vibration of the peptide groups	√	√	√	√	√	√	√	√	√	√	√	√

Section S6. Additional results of SERS spectral understanding.

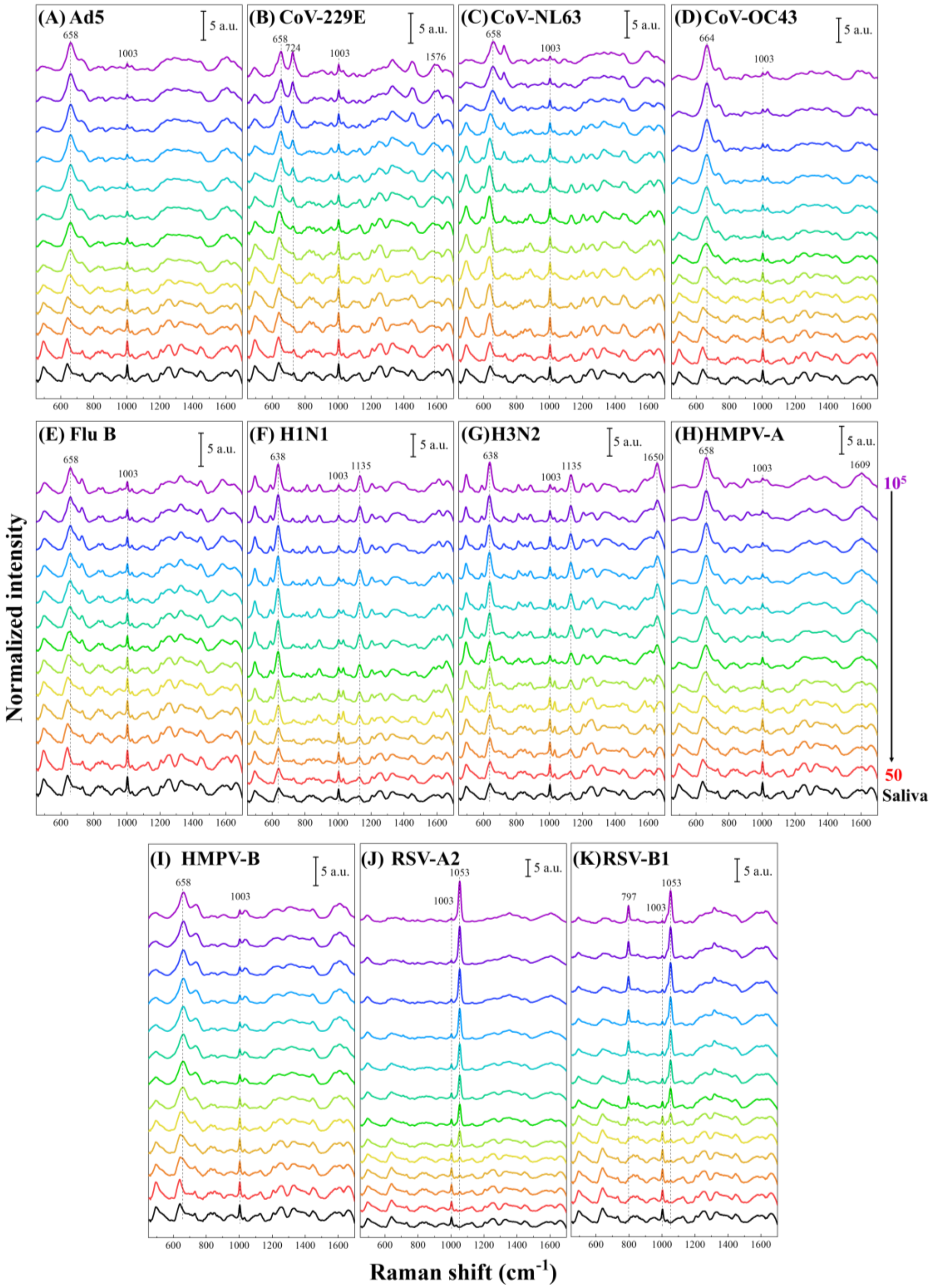

Figure S6. Concentration-dependent average SERS spectra of SVs in saliva. (**A**) Ad5, (**B**) CoV-229E, (**C**) CoV-NL63, (**D**) CoV-OC43, (**E**) Flu B, (**F**) H1N1, (**G**) H3N2, (**H**) HMPV-A, (**I**) HMPV-B, (**J**) RSV-A2, (**K**) RSV-B1. The concentrations vary from 50 to 10^5 PFU/mL. The black curves are the SERS spectra of saliva as a reference.

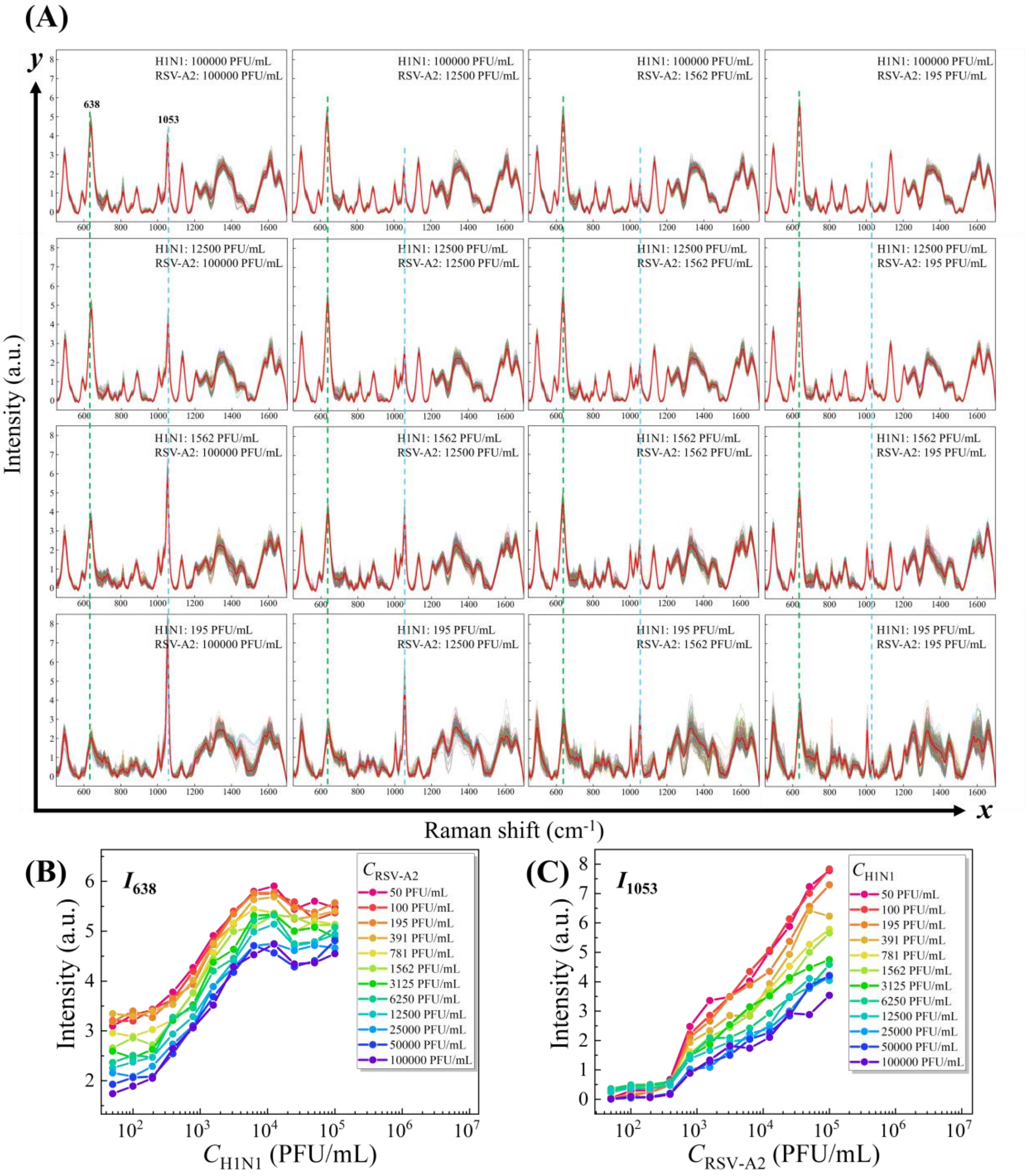

Figure S7. H1N1 & RSV-A2. (**A**) The normalized SERS spectra from some representative concentration combinations of H1N1 & RSV-A2. The concentrations gradually change from 10^5 to 195 PFU/mL, with the *x*-axis direction representing the concentration of RSV-A2, and the *y*-axis direction representing the concentration of H1N1. In each subfigure, all the individual SERS spectra from one concentration combination of the virus mixture are plotted, with the average SERS spectra shown by the bold red curve. The plots of (**B**) normalized peak intensities I_{638} at different C_{H1N1} and (**C**) the normalized peak intensities I_{1053} at different $C_{RSV\text{-}A2}$.

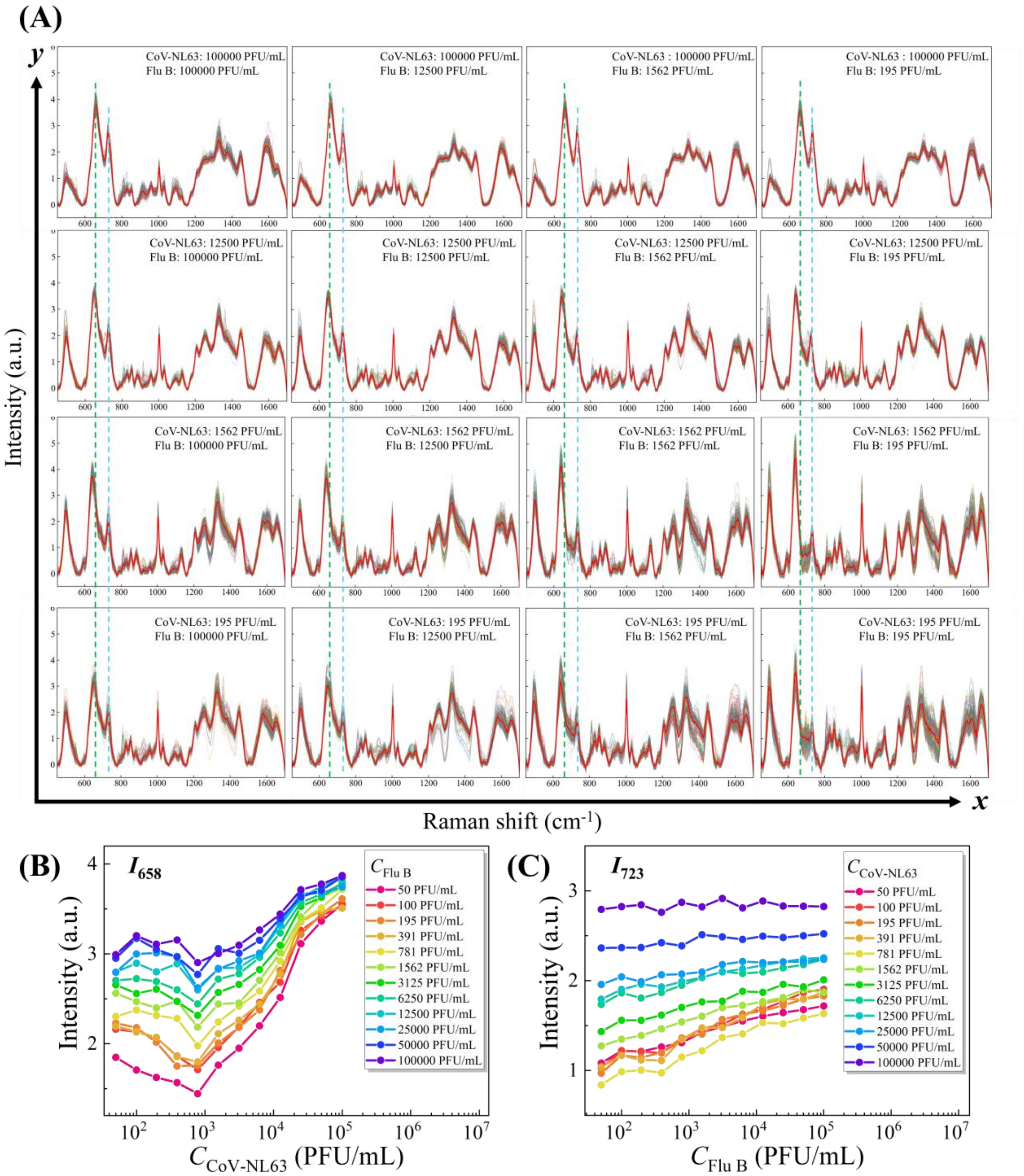

Figure S8. CoV-NL63 & Flu B. (**A**) The normalized SERS spectra from some representative concentration combinations of CoV-NL63 & Flu B. The concentrations gradually change from 10^5 to 195 PFU/mL, with the *x*-axis direction representing the concentration of Flu B, and the *y*-axis direction representing the concentration of CoV-NL63. In each subfigure, all the individual SERS spectra from one concentration combination of the virus mixture are plotted, with the average SERS spectra shown by the bold red curve. The plots of (**B**) normalized peak intensities I_{658} at different $C_{\text{CoV-NL63}}$ and (**C**) normalized peak intensities I_{723} at different $C_{\text{Flu B}}$.

Clearly, for mixtures with viruses whose SERS spectra have distinct peaks, it is expected that the normalized intensities of the distinct peaks unique to each of the two viruses change with the concentration of each virus. Taking H1N1 & RSV-A2 as an example, **Figure S7A** plots the normalized SERS spectra at representative concentrations of H1N1 & RSV-A2, gradually varying from 10^5 to 195 PFU/mL. Characteristic peaks at $\Delta\nu = 638$ cm^{-1} increase with C_{H1N1} (**Figure S7B**), and peaks at $\Delta\nu = 1053$ cm^{-1} increase with $C_{\mathrm{RSV\text{-}A2}}$ (**Figure S7C**). **Figures S9A** and **S9B** plot the 2D heat maps of normalized peak intensities I_{638} and I_{1053} versus C_{H1N1} (x-axis) and $C_{\mathrm{RSV\text{-}A2}}$ (y-axis) from SERS spectra of H1N1 & RSV-A2 mixtures, respectively. The color changes along the diagonal, with the upper-left corner showing the lowest $I_{638} = 1.74$, and the bottom-right corner showing the highest $I_{638} = 5.47$, which demonstrates I_{638} is proportional to C_{H1N1}. Conversely, an inverse color gradient is observed for I_{1053}, changing from 7.78 to 0.01, which also demonstrates I_{1053} is proportional to C_{RSV-A2}.

In order to analyze the trend quantitatively, the concept of the Pearson correlation coefficient is used. Let $Corr(I_{A1},\ I_{A2})$ be the Pearson correlation coefficient between the SERS spectra I_{A1} and I_{A2} of two analytes $A1$ and $A2$, then:

$$Corr(I_{A1}, I_{A2}) = \frac{\sum\left(I_{A1}^i - \overline{I_{A1}}\right)\left(I_{A2}^i - \overline{I_{A2}}\right)}{\sqrt{\sum\left(I_{A1}^i - \overline{I_{A1}}\right)^2 \sum\left(I_{A2}^i - \overline{I_{A2}}\right)^2}}, \tag{S1}$$

where I_{A1}^i and I_{A2}^i are the intensities of the i-th Raman shift of I_{A1} and I_{A2}, $\overline{I_{A1}} = \frac{1}{N}\sum_{i=1}^{N} I_{A1}^i$ and $\overline{I_{A2}} = \frac{1}{N}\sum_{i=1}^{N} I_{A2}^i$ are the average intensity values of I_{A1} and I_{A2}. **Figures S9C** and **S9D** plot the 2D heat maps of correlation coefficients $Corr(I_{\mathrm{H1N1\,\&\,RSV-A2}}, I_{\mathrm{H1N1}})$ and $Corr(I_{\mathrm{H1N1\,\&\,RSV-A2}}, I_{\mathrm{RSV-A2}})$ versus C_{H1N1} and C_{RSV-A2}, respectively. I_{H1N1} and I_{RSV-A2} are from the SERS spectra of their corresponding highest concentrations. In **Figure S9C,** the upper-left corner shows the lowest $Corr(I_{H1N1\,\&\,RSV-A2}, I_{H1N1}) = 0.32$, and the bottom-right corner shows the highest $Corr(I_{H1N1\,\&\,RSV-A2}, I_{H1N1}) = 0.99$. The color changes along the diagonal, which demonstrates $Corr(I_{H1N1\,\&\,RSV-A2}, I_{H1N1})$ is proportional to C_{H1N1}. An inverse trend can be observed for $Corr(I_{H1N1\,\&\,RSV-A2}, I_{RSV-A2})$ as shown in **Figure S9D**, with the values changing from 0.95 to 0.16 along the diagonal, which also indicates $Corr(I_{H1N1\,\&\,RSV-A2}, I_{RSV-A2})$ is proportional to C_{RSV-A2}. **Figure S9A** and **Figure S9C** are similar to each other, as are **Figure S9B** and **Figure**

S9D. When C_{H1N1} is high, $I_{H1N1\,\&\,RSV-A2}$ is similar with I_{H1N1}. However, their contributions (weights) to the SERS spectra are not 1:1. This discrepancy is related to virus concentrations and their adsorption capabilities.

For virus mixtures of CoV-NL63 & Flu B, both CoV-NL63 and Flu B show similar SERS peaks, **Figure S8** plots representative SERS spectra from various concentration combinations, with the concentrations gradually changing from 10^5 to 50 PFU/mL. However, there is almost no clear trend of peak intensity increase or decrease (**Figures S9E- S9F**) or change in the correlation coefficient (**Figures S9G- S9H**) along the diagonal with changes in virus concentration, due to the overlapping peaks of these two viruses. The normalized I_{658} increases with $C_{\text{CoV-NL63}}$ first and then decreases (**Figure S8B**), while the normalized I_{723} increases with $C_{\text{Flu B}}$ (**Figure S8C**). **Figure 2 S9E** and **Figure S9G** are similar to each other, as are **Figure S9F** and **Figure S9H**.

For 3VMs, specifically the CoV-NL63 & H1N1 & RSV-B1, **Figures S9I- S9K** plot 3D heat maps of the normalized peak intensities of I_{658}, I_{638}, and I_{1053} versus $C_{\text{CoV-NL63}}$ (x-axis), C_{H1N1} (y-axis), and $C_{\text{RSV-B1}}$ (z-axis) from the SERS spectra of the mixture, respectively. In **Figure S9I**, regardless of C_{H1N1} and $C_{\text{RSV-B1}}$, I_{658} is large at high $C_{\text{CoV-NL63}}$ and small at low $C_{\text{CoV-NL63}}$. At each layer of $C_{\text{RSV-B1}}$, when $C_{\text{H1N1}} = 10^5$ PFU/mL and $C_{\text{CoV-NL63}}$ is at its lowest, I_{658} is smallest. Similarly, when C_{H1N1} is fixed, I_{658} is smallest when $C_{\text{RSV-B1}} = 10^5$ PFU/mL. When $C_{\text{RSV-B1}} = C_{\text{H1N1}} = 10^5$ PFU/mL and $C_{\text{CoV-NL63}} = 195$ PFU/mL, I_{658} is smallest. Conversely, when $C_{\text{RSV-B1}} = C_{\text{H1N1}} = 195$ PFU/mL and $C_{\text{CoV-NL63}} = 10^5$ PFU/mL, I_{658} is largest. This is because $\Delta\nu = 658$ cm^{-1} is a unique characteristic peak of CoV-NL63, which the other two viruses do not have. The changes of I_{658} accurately indicate the relative change of $C_{\text{CoV-NL63}}$ in the CoV-NL63 & H1N1 & RSV-B1 mixture. Similar observations can be found in **Figure S9J** and **Figure S9K** since $\Delta\nu = 638$ and $\Delta\nu = 1053$ cm^{-1} are unique for H1N1 and RSV-B1, respectively. **Figures S9L-N** plot the 3D map of correlation coefficient $Corr(I_{\text{CoV-NL63\&H1N1\&RSV-B1}}, I_{\text{Vi}})$ versus $C_{\text{CoV-NL63}}$ (x-axis), C_{H1N1} (y-axis), and $C_{\text{RSV-B1}}$ (z-axis) between the SERS spectra from mixtures and SERS spectra from highest concentrations of CoV-NL63, H1N1, and RSV-B1, respectively. The color gradient follows the diagonal of the rectangular cuboid heatmap, especially for the correlation coefficient $Corr(I_{\text{CoV-NL63\&H1N1\&RSV-B1}}, I_{\text{RSV-B1}})$ in **Figure S9N**. There is a larger portion of red color on the top layer where $C_{RSV-B1} = 10^5$ PFU/mL, and a larger portion of blue color (smaller correlation) on the bottom layer where $C_{RSV-B1} = 195$ PFU/mL. On each layer, the color gradient also follows the diagonal. These color changes demonstrate that $Corr\ (I_{\text{CoV-NL63 \& H1N1 \& RSV-B1}}, I_{\text{RSV-B1}})$ is proportional to

C_{RSV-B1}. For I_{638} and I_{658}, the color gradients are not exactly along the diagonal of the rectangular cuboid, but they show a similar trend, indicating that $Corr(I_{\text{CoV-NL63\&H1N1\&RSV-B1}}, I_{\text{CoV-NL63}})$ is proportional to $C_{CoV-NL63}$ (**Figure S9L**), and $Corr(I_{\text{CoV-NL63\&H1N1\&RSV-B1}}, I_{\text{H1N1}})$ is proportional to C_{H1N1} (**Figure S9M**). Clearly, **Figure S9L** resembles **Figure S9I**, as does **Figure S9M** to **Figure S9J**, and **Figure S9N** to **Figure S9K.**

Significant challenges arise in virus co-infection detections: (1) When dealing with multiple virus mixtures, obtaining accurate results can be difficult, especially if one or all virus concentrations are low. (2) The complexity of detection increases with the number of viruses in the mixture, as adding an additional virus raises the dimensionality of the concentration space. Classic analysis methods, such as calibration curves, perform well for detecting single or dual virus infections (**Figure S9** and **Figure S10**). However, these methods become ineffective when the dimensionality is three or more. For unknown specimens, virus identification is often based on symptoms, but many respiratory viruses present similar symptoms, complicating the diagnosis. (3) Another significant challenge arises due to the similarity in SERS spectra among viruses, especially when virus mixtures contain different variants, such as H1N1 & RSV-A2, H1N1 & RSV-B1, H3N2 & RSV-A2, and H3N2 & RSV-B1 (**Figures 1F** and **1I**). It is difficult to differentiate the virus variants based solely on their characteristic SERS peaks. The SERS spectra of H1N1 and H3N2 are similar, and likewise, the SERS spectra of RSV-A2 and RSV-B1 are also similar. The SERS spectra of influenza and RSV mixtures with different variants exhibit strikingly similar patterns, especially when the virus concentrations vary.

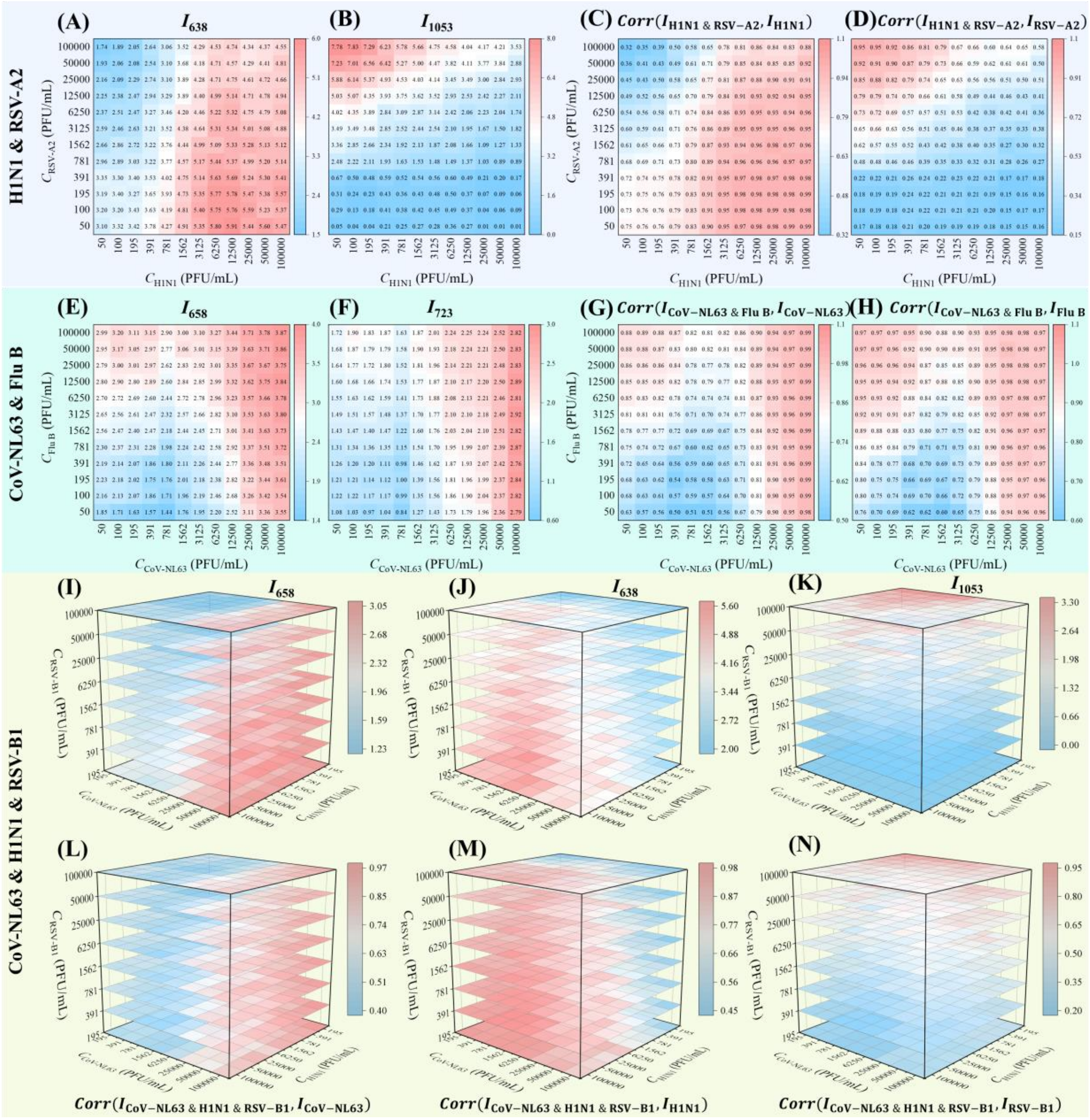

Figure S9. SERS spectra understanding of virus mixtures. Two-virus mixture of H1N1 & RSV-A2: the 2D heat maps of normalized SERS peak intensities (**A**) I_{638} and (**B**) I_{1053} versus C_{H1N1} and $C_{RSV\text{-}A2}$. The 2D heat maps of correlation coefficients versus C_{H1N1} and $C_{RSV\text{-}A2}$: (**C**) $Corr(I_{H1N1\ \&\ RSV-A2}, I_{H1N1})$ and (**D**) $Corr(I_{H1N1\ \&\ RSV-A2}, I_{RSV-A2})$. Similar plots for CoV-NL63 & Flu B: (**E**) I_{658}, (**F**) I_{723}, (**G**) $Corr(I_{CoV-NL63\ \&\ Flu\ B}, I_{CoV-NL63})$, and (**H**) $Corr(I_{CoV-NL63\ \&\ Flu\ B}, I_{Flu\ B})$. Three-virus mixture of CoV-NL63 & H1N1 & RSV-B1: the 3D heat maps normalized peak intensity (**I**) I_{658}, (**J**) I_{638}, and (**K**) I_{1053} versus $C_{CoV\text{-}NL63}$ (*x*-axis), C_{H1N1} (*y*-axis), and $C_{RSV\text{-}B1}$ (*z*-axis). The 3D maps of correlation coefficients versus $C_{CoV\text{-}NL63}$, C_{H1N1}, and $C_{RSV\text{-}B1}$: (**L**) $Corr(I_{CoV-NL63\ \&\ H1N1\ \&\ RSV-B1}, I_{CoV-NL63})$, (**M**) $Corr(I_{CoV-NL63\ \&\ H1N1\ \&\ RSV-B1}, I_{H1N1})$ and (**N**) $Corr(I_{CoV-NL63\ \&\ H1N1\ \&\ RSV-B1}, I_{RSV-B1})$.

Section S7. Traditional calibration curves.

Some representative calibration curves for virus co-infection detections are constructed, as shown in **Figure S10**. For single-virus detection, the original SERS peak intensities I_{724} and I_{638} are plotted against virus concentrations $C_{\mathrm{CoV-NL63}}$ and C_{H3N2} in **Figures S10A-B**, respectively, on a semi-log scale, respectively. SERS intensities increase monotonically with concentration, although the detailed relationships are not exactly the same. In fact, the original SERS peak intensities do not show a distinguishable change at low virus concentrations. According to the definition of the limit of detection (LOD), which is the SERS peak intensity greater than three times the standard deviation of the background SERS signal from blank saliva specimens, the LODs for CoV-NL63 and H1N1 are estimated to be 6250 PFU/mL and 781 PFU/mL, respectively. For 2VM detection, e.g., CoV-NL63 & RSV-A2, the original SERS peak intensities I_{724} and I_{1053}, are plotted against virus concentrations $C_{\mathrm{CoV-NL63}}$ and $C_{\mathrm{RSV-A2}}$ in virus mixtures, as shown in **Figures S10C**, on a semi-log scale. The LODs for CoV-NL63 & RSV-A2 are estimated to be (6250, 391) PFU/mL, and the LODs for H3N2 & RSV-A2 are estimated to be (1562, 781) PFU/mL (**Figures S10D**). For 3VM detection as shown in **Figures S10E-F**, the LODs for CoV-NL63 & H1N1 & RSV-A2 and CoV-NL63 & H3N2 & RSV-B1 are estimated to be (6250, 781, 781) PFU/mL and (6250, 781, 1562) PFU/mL, respectively.

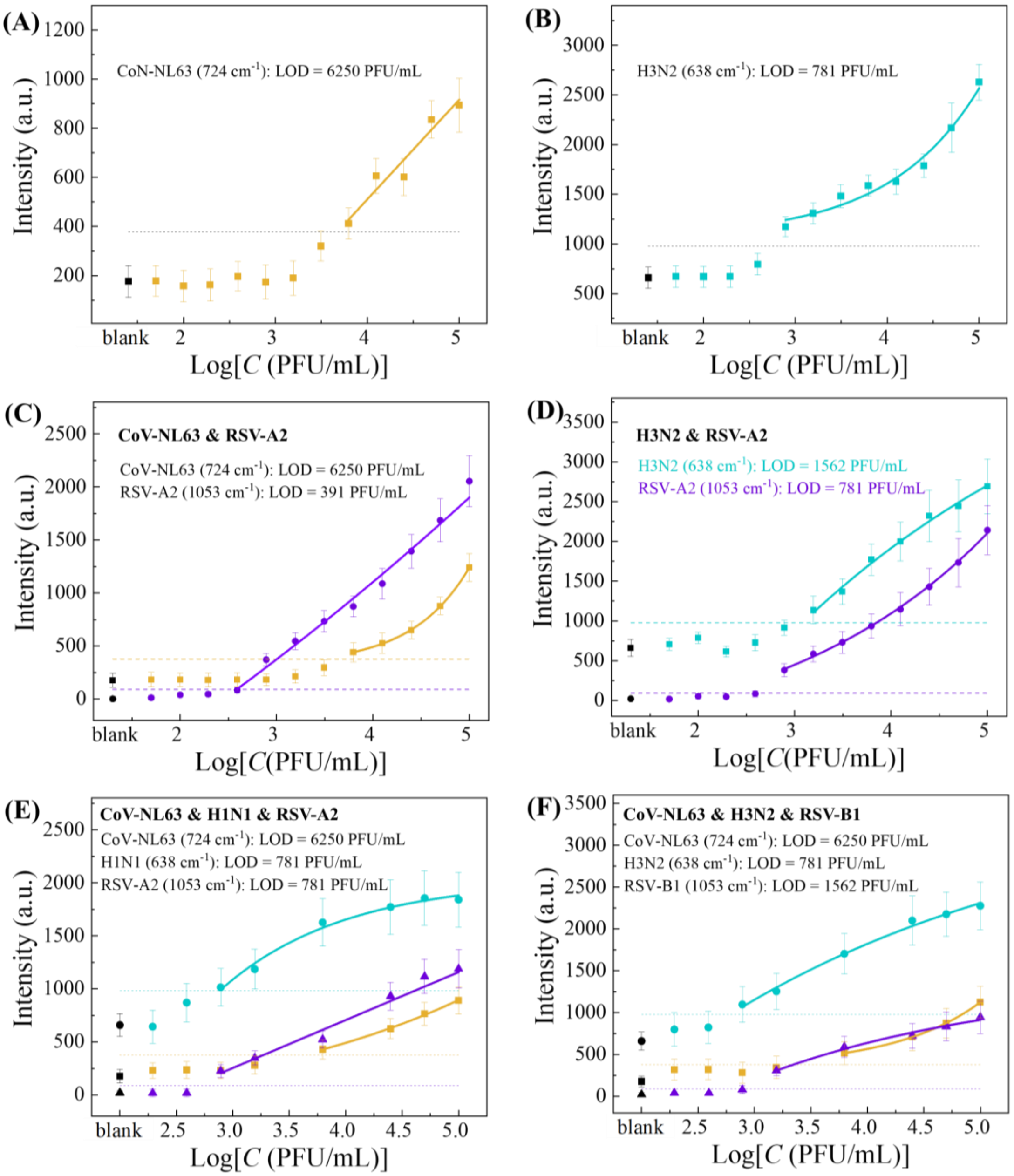

Figure S10. Traditional calibration curves. (A) The plot of the original SERS peak intensity I_{724} versus $C_{\mathrm{CoV\text{-}NL63}}$ for SV detection of CoV-NL63. (B) The plot of I_{638} versus C_{H3N2} for SV detection of H3N2. (C) The plots of I_{724} and I_{1053} versus $C_{\mathrm{CoV\text{-}NL63}}$ and $C_{\mathrm{RSV\text{-}A2}}$, respectively for the detection of CoV-NL63 & RSV-A2. (D) The plots of I_{638} and I_{1053} versus C_{H3N2} and $C_{\mathrm{RSV\text{-}A2}}$, respectively for the detection of H3N2 & RSV-A2. (E) The plots of I_{724}, I_{638}, and I_{1053} versus $C_{\mathrm{CoV\text{-}NL63}}$, C_{H3N2}, and $C_{\mathrm{RSV\text{-}A2}}$, respectively for the detection of CoV-NL63 & H1N1 & RSV-A2. (F) The plots of I_{724}, I_{638}, and I_{1053} versus $C_{\mathrm{CoV\text{-}NL63}}$, C_{H3N2}, and $C_{\mathrm{RSV\text{-}A2}}$, respectively for the detection of CoV-NL63 & H1N1 & RSV-A2. All plots are in semi-log scale. The black data points are from corresponding Raman shifts of saliva, representing the background signal.

Section S8. Performance of traditional machine learning models.

Random forest (RF)[44] is a widely used ensemble machine learning algorithm that constructs multiple decision trees during training and outputs the average prediction (for regression) or the majority vote (for classification) of these trees. Its ability to handle nonlinear relationships, model interactions between variables, and resist overfitting makes RF a strong baseline model for analytical tasks. In this study, the RF model was configured with 100 trees, which is commonly considered sufficient for both classification and regression tasks. This ensures the ensemble has enough diversity to generalize effectively without excessive computational overhead. The maximum depth of the trees was not limited, i.e., the maximum depth is infinity. These decisions were based on the complexity of the dataset, which included over 1 million SERS spectra. Limiting the depth could negatively impact the model's ability to fully capture the relationships within this high-dimensional dataset, potentially reducing performance.

In the initial training phase, the RF model was applied to the entire SERS spectral dataset, covering all concentrations from SVs, 2VMs, and 3VMs, including 3,476 kinds of specimens with a total of 1,213,550 SERS spectra. The SERS spectra were randomly divided into training, validation, and testing spectral sets in an 8:1:1 ratio using stratified sampling. However, the overall performance of RF fell short of expectations, achieving a classification accuracy of 81.5 ± 0.6% for virus mixture classification, and an MAE of 0.169 ± 0.008 for virus concentration regression. These results suggest that while RF can capture basic patterns in the data, it struggles with the high dimensionality and subtle spectral differences inherent in SERS spectra of complex virus mixtures.

Five independent trials of training the RF model are conducted, and the overall accuracy A is 81.5 ± 0.6% for virus mixture classification. The trial of the best performance for classification is summarized by a confusion matrix in **Figure S11**. Along the diagonal of the confusion matrix, the A values range from 77.4% to 98.9%. The misclassifications range from 0.01% to 21.2%. Among them, the average accuracy for SV classification is 96.15%, whereas for 2VM and 3VM classifications, the average accuracies drop to 80.89% and 80.74%, respectively. It is expected that the classification accuracy shall vary at different virus concentrations. In particular, the accuracy decreases at low concentrations. For SV classification, most viruses are classified with high accuracy (>95%). However, saliva presents a notable exception, with an accuracy of only 78.8%, and 21.2% of saliva samples misclassified as CoV-NL63. This highlights a key limitation in distinguishing between saliva and CoV-NL63 spectra. For mixtures, the classification performance shows a significant gap compared to SV. Among 2VMs, the highest accuracy is only 86.1%, with 5 out of 9 virus pairs achieving accuracies below 80%. For 3VMs, the classification accuracy further declines, with a maximum value of 82.4%. These results indicate that while the RF model performs well for simpler cases, its accuracy decreases substantially for more complex virus mixtures and at lower

concentrations. This underscores the need for more robust approaches to improve classification performance in these challenging scenarios.

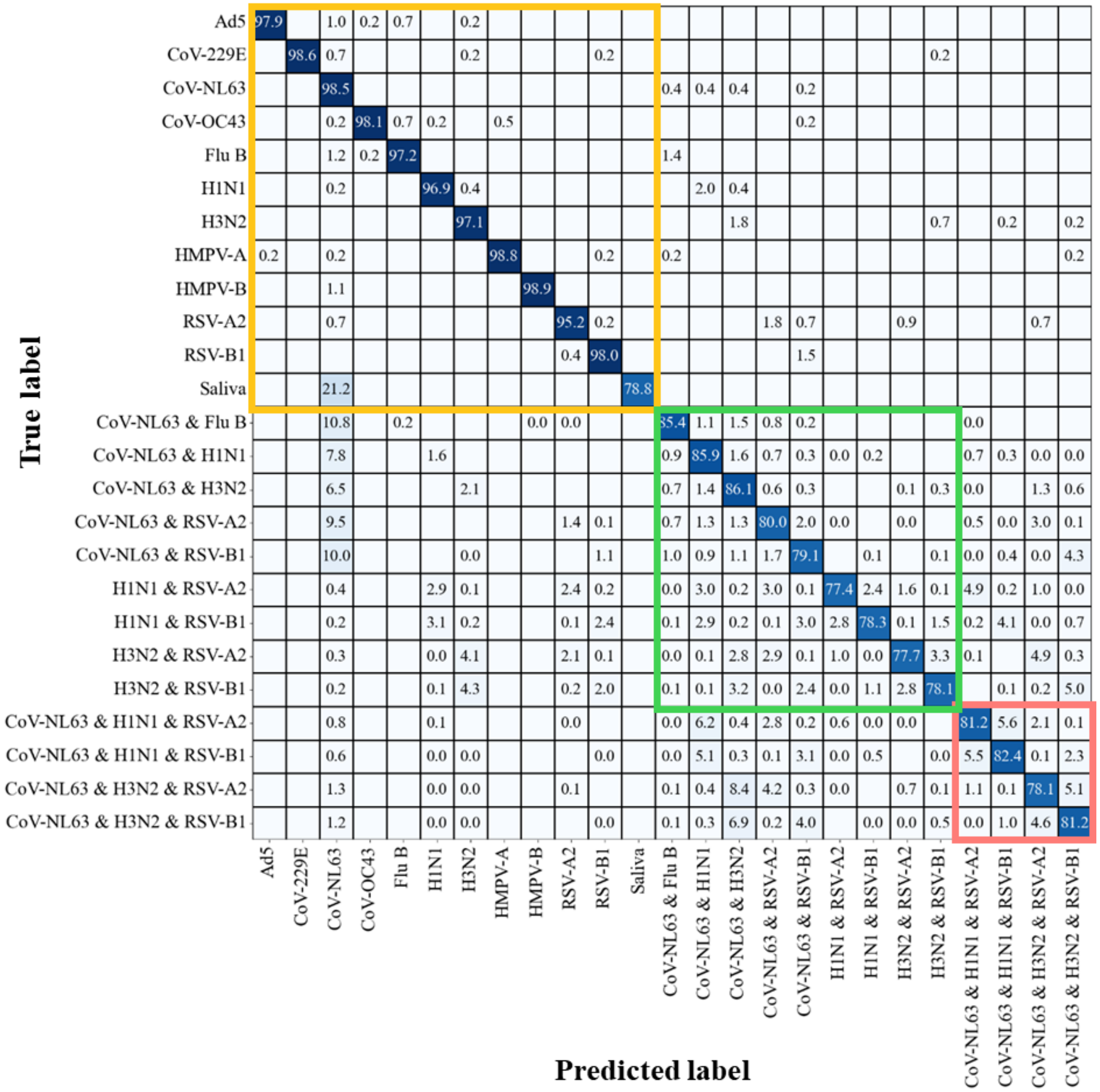

Figure S11. Confusion matrix of the RF model trained with SERS spectra of all concentrations in experiments from eleven SVs, nine 2VMs, and four 3VMs in saliva, as well as the reference saliva. The matrix entries represent the percentage of test spectra predicted as a specific class (first row) given a ground truth of class (first column). Diagonal entries show the accuracy for each class. Note that "0.0" indicates values below 0.04%, rather than an actual zero.

The regression performance of the RF model was evaluated across 5 independent trials, using spectra with concentrations above the LODs as described in **Section S9**. The MAE and coefficient of

determination (R^2) for different virus mixtures are summarized in **Table S4**, with standard deviations calculated across the trials. The RF model achieved an overall MAE of 0.169 ± 0.008 and an average R^2 of 0.874 ± 0.003 across all virus mixtures. While these results indicate reasonable overall performance, a closer examination of individual components reveals notable challenges, particularly for specific single viruses (SVs) and 3VMs.

For single virus regression, the average R^2 across all viruses is 0.854, with most viruses achieving R^2 values above 0.8. However, CoV-OC43 stands out with a much lower R^2 of 0.695, indicating significant difficulty in accurately predicting its concentrations. This is reflected in its relatively high MAE of 0.283 ± 0.006. The regression performance declines further for three-virus mixtures, with an average R^2 of 0.824. The maximum R^2 observed in 3VM regression is only 0.928, and only 3 out of 12 components have R^2 values above 0.9. This highlights the difficulty in accurately quantifying concentrations when three viruses are present, as the spectra become increasingly complex and overlapping. For example, CoV-NL63 in CoV-NL63 & H3N2 & RSV-A2 achieves an R^2 of 0.891 ± 0.001, while RSV-A2 in the same mixture has a significantly lower R^2 of 0.737 ± 0.001. These results demonstrate the difficulty of using RF to quantify the concentrations for complex virus mixtures and highlight the need for more advanced approaches to improve regression performance.

Table S4. The MAE and R^2 list of different virus mixtures across 5 independent trials of the RF model (± calculated as standard deviation), tested with spectra of $C_{virus} \geqslant$ LODs (see **Section S8**).

Virus species	Component MAE	Component R^2
Ad5	0.234 ± 0.003	0.796 ± 0.007
CoV-229E	0.283 ± 0.004	0.768 ± 0.005
CoV-NL63	0.133 ± 0.001	0.950 ± 0.001
CoV-OC43	0.283 ± 0.006	0.695 ± 0.007
Flu B	0.279 ± 0.002	0.758 ± 0.008
H1N1	0.075 ± 0.002	0.967 ± 0.001
H3N2	0.086 ± 0.001	0.969 ± 0.000
HMPV-A	0.160 ± 0.001	0.894 ± 0.002
HMPV-B	0.171 ± 0.003	0.898 ± 0.002
RSV-A2	0.271 ± 0.002	0.806 ± 0.004
RSV-B1	0.129 ± 0.001	0.947 ± 0.001
CoV-NL63 & Flu B	(0.081 ± 0.000, 0.254 ± 0.001)	(0.980 ± 0.000, 0.844 ± 0.000)
CoV-NL63 & H1N1	(0.089 ± 0.001, 0.057 ± 0.000)	(0.960 ± 0.001, 0.980 ± 0.001)
CoV-NL63 & H3N2	(0.106 ± 0.000, 0.073 ± 0.000)	(0.922 ± 0.001, 0.940 ± 0.002)
CoV-NL63 & RSVA2	(0.090 ± 0.000, 0.247 ± 0.000)	(0.958 ± 0.000, 0.759 ± 0.001)
CoV-NL63 & RSVB1	(0.109 ± 0.001, 0.288 ± 0.001)	(0.945 ± 0.000, 0.712 ± 0.002)
H1N1 & RSV-A2	(0.116 ± 0.000, 0.230 ± 0.001)	(0.938 ± 0.001, 0.828 ± 0.001)
H1N1 & RSV-B1	(0.111 ± 0.000, 0.225 ± 0.001)	(0.942 ± 0.001, 0.835 ± 0.002)
H3N2 & RSV-A2	(0.099 ± 0.000, 0.250 ± 0.000)	(0.953 ± 0.000, 0.796 ± 0.001)
H3N2 & RSV-B1	(0.087 ± 0.000, 0.250 ± 0.001)	(0.961 ± 0.001, 0.814 ± 0.001)

CoV-NL63 & H1N1 & RSV-A2	(0.145 ± 0.000, 0.189 ± 0.000, 0.132 ± 0.000)	(0.912 ± 0.001, 0.838 ± 0.001, 0.897 ± 0.000)
CoV-NL63 & H1N1 & RSV-B1	(0.131 ± 0.000, 0.192 ± 0.001, 0.163 ± 0.000)	(0.928 ± 0.000, 0.831 ± 0.001, 0.880 ± 0.001)
CoV-NL63 & H3N2 & RSV-A2	(0.166 ± 0.001, 0.222 ± 0.000, 0.153 ± 0.000)	(0.891 ± 0.001, 0.737 ± 0.001, 0.856 ± 0.001)
CoV-NL63 & H3N2 & RSV-B1	(0.152 ± 0.000, 0.209 ± 0.001, 0.163 ± 0.001)	(0.909 ± 0.001, 0.767 ± 0.001, 0.886 ± 0.001)

Due to the high-dimensional nature of the SERS spectra, the trained RF model required 11.7 GB of storage. This considerable size presents a significant limitation for practical applications, especially for deployment on ordinary PCs or portable devices. The size and computational requirements of RF highlight one reason why traditional machine learning methods may not be ideal for this study, particularly when compared to the efficiency and scalability of deep learning models.

Section S9. Optimization of deep learning models.

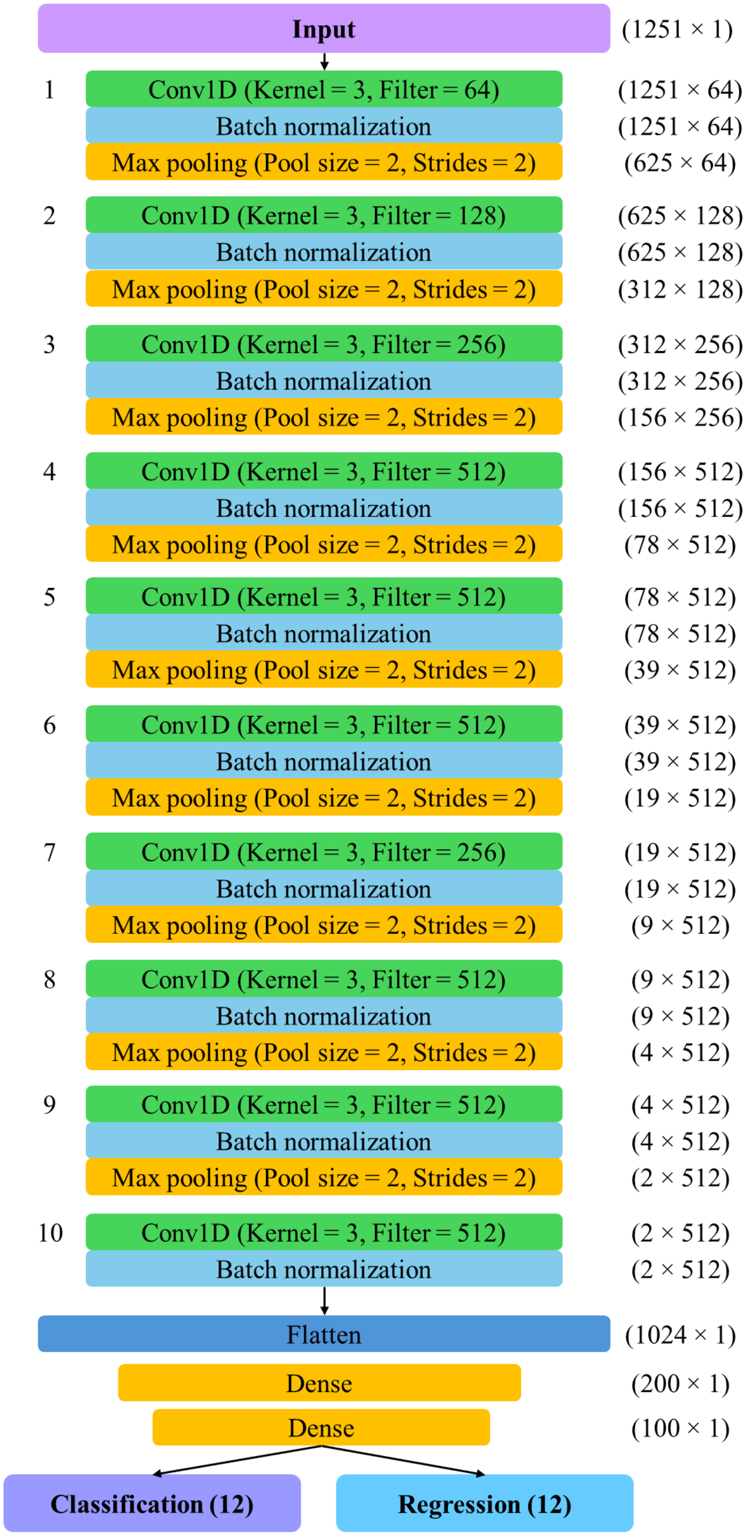

Figure S12. The detailed architecture of the MultiplexCR model with 10 sequential convolutional blocks.

The final outputs of the MultiplexCR algorithm for a given SERS spectrum are two 12-element vectors, [V_1, V_2, …, V_{12}] and [C_1, C_2, …, C_{12}]. The element V_i represents the absence or presence of an ith virus with a value $V_i = 0$ or 1, and the element C_i indicates the log10 concentration of the ith virus. The order of these eleven virus classes, V_1 to V_{11}, from left to right, is Ad5, CoV-229E, CoV-NL63, CoV-OC43, Flu B, H1N1, H3N2, HMPV-A, HMPV-B, RSV-A2, RSV-B1, with V_{12} representing saliva. For classification, the cross-entropy is computed based on the predicted virus types $[V_1, V_2, \dots, V_{12}]$ against the actual virus types; while for regression, the mean absolute error (MAE) is calculated for the virus concentration predictions. The overall loss function is a linear combination of these two. However, there can be two kinds of loss functions for the model optimization. First, the original loss function (with original MAE) is defined as

$$Loss = a \times \sum_{i=1}^{12} p_i \log(\hat{p}_i) + b \times \frac{1}{4} \sum_{i=1}^{12} \left| C_i - \hat{C}_i \right|, \quad \text{(S2)}$$

where p_i and $\hat{p}_i$ are the true and predicted probabilities of V_i, C_i and $\hat{C}_i$ are the true virus concentration and predicted virus concentrations, respectively. Second, a custom loss function (with custom MAE) is defined as

$$Loss = a \times \sum_{i=1}^{12} p_i \log(\hat{p}_i) + b \times \frac{1}{12} \sum_{i=1}^{12} \left| C_i - \hat{C}_i \right| V_i. \quad \text{(S3)}$$

The coefficients $a = 1$ and $b = 1000$ are determined via multiple test runs to optimize the classification and regression performance. This loss function ensures that the model focuses only on desired predictions, optimizing computational resources by not calculating unnecessary concentrations.

Overall, the classification and regression loss functions decrease with training epochs, as shown in **Figure S13**. When optimizing the MultiplexCR model based on **Eq. S2**, the accuracies for the datasets containing SERS spectra of all concentrations in the experiments are 95.9% for training and 88.3% for validation after 500 epochs (**Figure S13A**), with custom MAEs of 0.054 for training and 0.142 for validation (**Figure S13B**). Obviously, there is a significant gap between training and validation loss even after 500 epochs, especially for regression loss, which means that there is a systematic error during the model optimization. This is because, in **Eq. S2**, the concentration loss also counts when $V_i = 0$, i.e., the concentration prediction also counts when the virus was classified as other virus type. However, when optimizing the MultiplexCR model based on **Eq. S3**, the accuracies for the datasets containing SERS spectra of all concentrations in the

experiments improve to 91.5% for training and 88.5% for validation after 500 epochs (**Figure S13D**), with custom MAEs of 0.010 for training and 0.033 for validation (**Figure S13E**). The custom MAE for the validation becomes much smaller. A similar phenomenon can be observed for datasets containing SERS spectra of $C_{\text{virus}} \geq$ LODs, as shown in **Figure S13G-S13L**. When using **Eq. S2**, the accuracies reach 97.8% for training and 97.5% for validation after 500 epochs (**Figure S13G**), with custom MAEs of 0.039 for training and 0.061 for validation (**Figure S13H**). In contrast, when optimizing with **Eq. S3**, the accuracies increase to 99.4% for training and 98.7% for validation after 500 epochs (**Figure S13J**), with custom MAEs of 0.005 for training and 0.023 for validation (**Figure S13K**). In addition, regarding the final performance of the SERS sensor, the original MAEs can be ignored (**Figures S13C, S13F, S13I,** and **S13L**). Therefore, better accuracies and smaller custom MAEs for validation are achieved based on **Eq. S3**. This custom MAE ensures the model focuses only on relevant concentration predictions, optimizing computational resources by avoiding unnecessary concentration calculations.

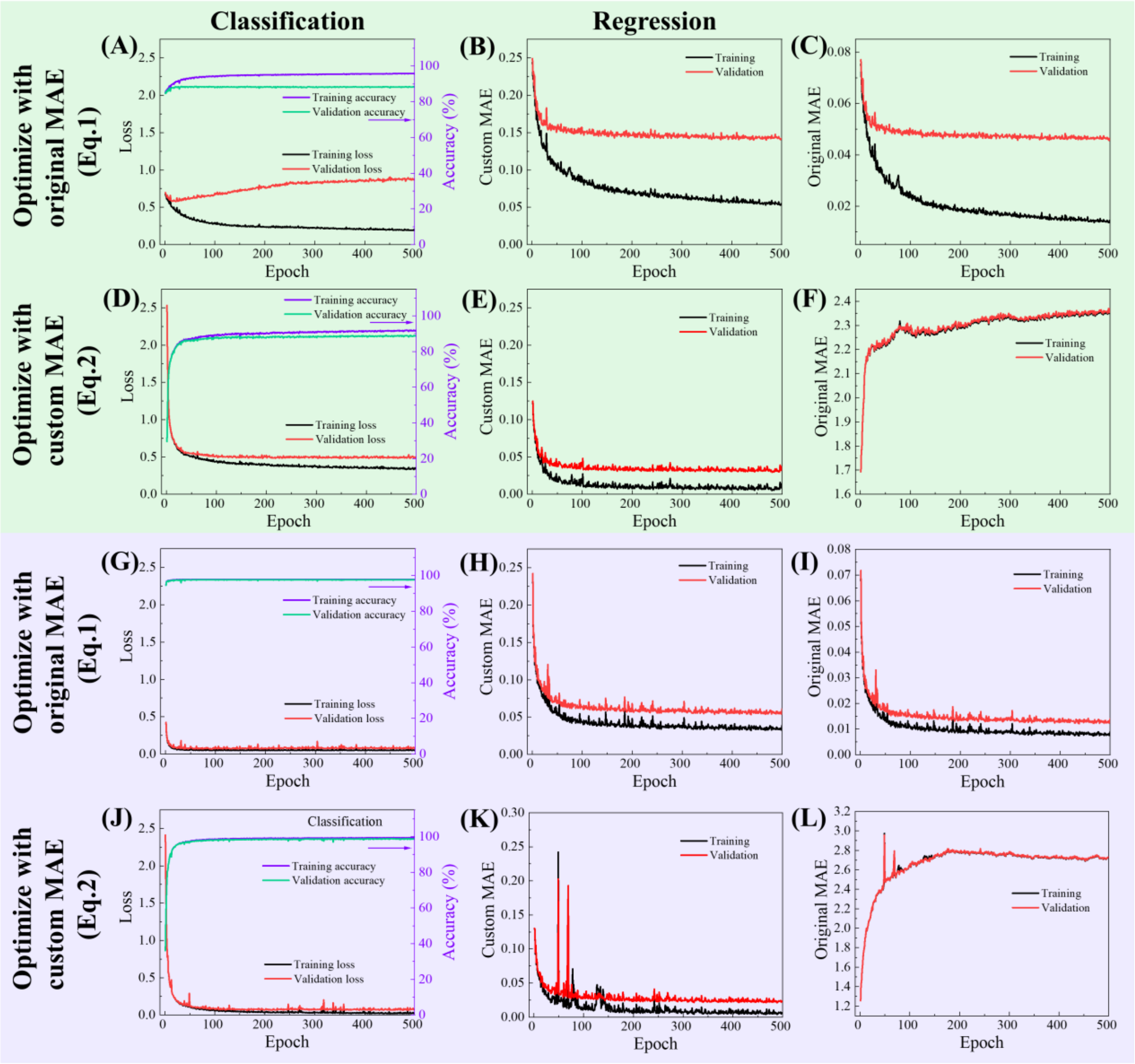

Figure S13. Performance metrics of the MultiplexCR algorithm for two different spectral datasets under different loss functions. Datasets containing SERS spectra of all concentrations in experiments: (**A**) Classification loss and accuracy, (**B**) custom MAE, and (**C**) original MAE based on **Eq. S1**. (**D**) Classification loss and accuracy, (**E**) custom MAE, and (**F**) original MAE based on **Eq. S2**. Datasets containing SERS spectra of $C_{\text{virus}} \geq$ LODs: (**G**) Classification loss and accuracy, (**H**) custom MAE, and (**I**) original MAE based on **Eq. S1**. (**J**) Classification loss and accuracy, (**K**) custom MAE, and (**L**) original MAE based on **Eq. S2**. All panels compare training and validation over training epochs.

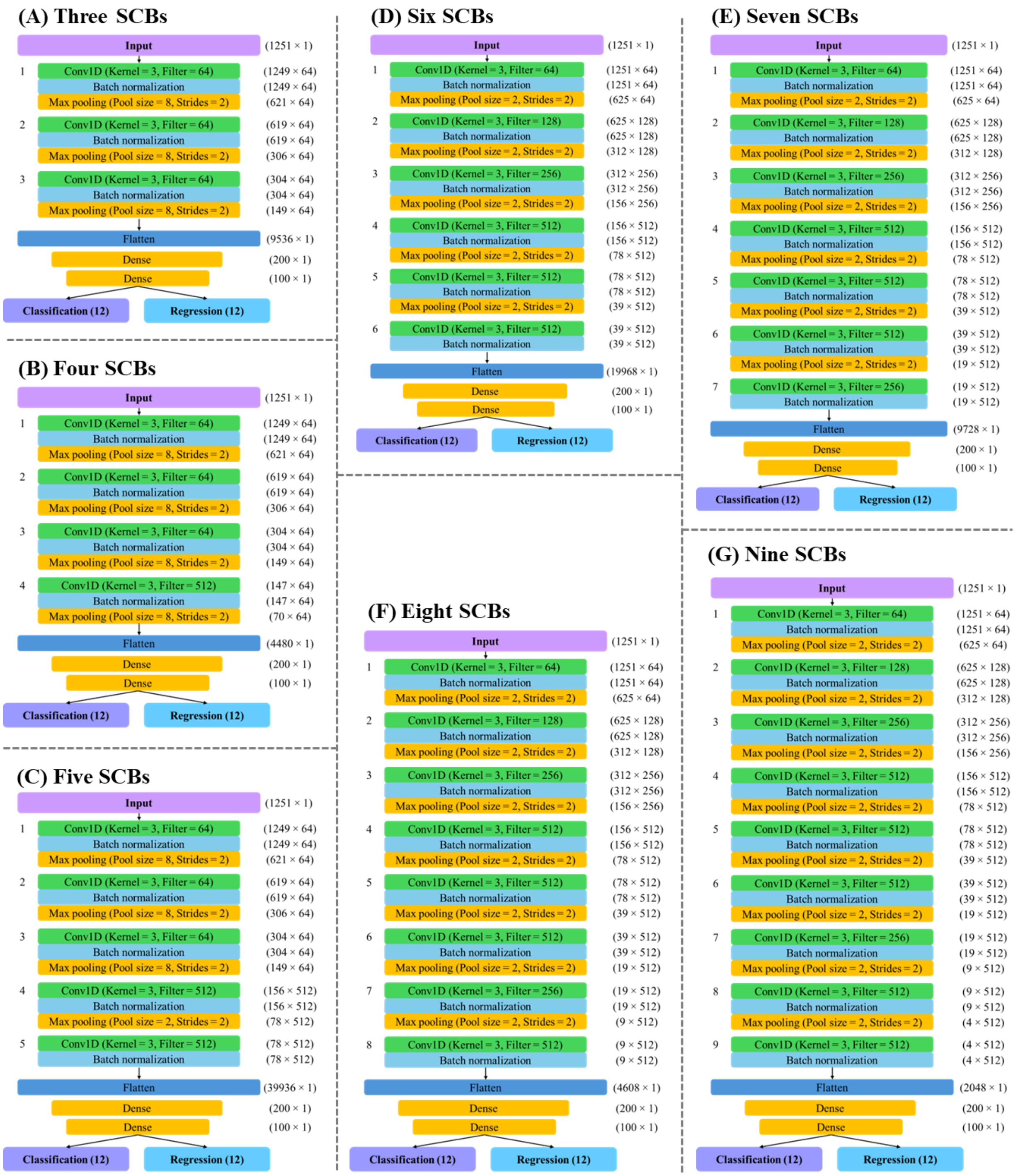

Figure S14. The detailed architectures of the MultiplexCR model with different sequential convolutional blocks (SCBs). (A) - (G) Three to nine SCBs.

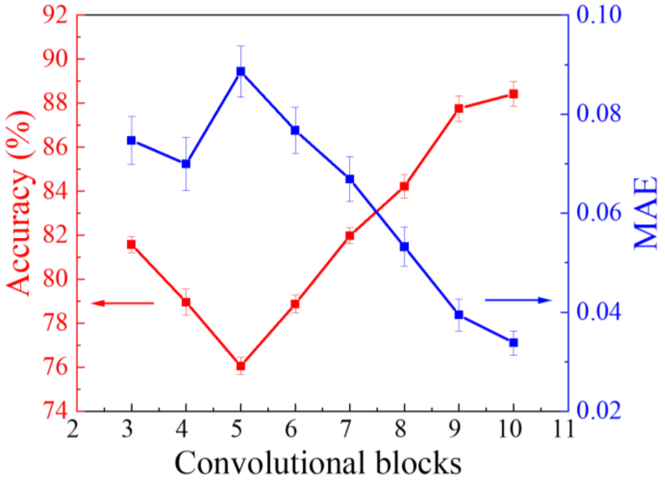

Figure S15. The variation in accuracy and MAE with respect to the number of sequential convolutional blocks in MultiplexCR models.

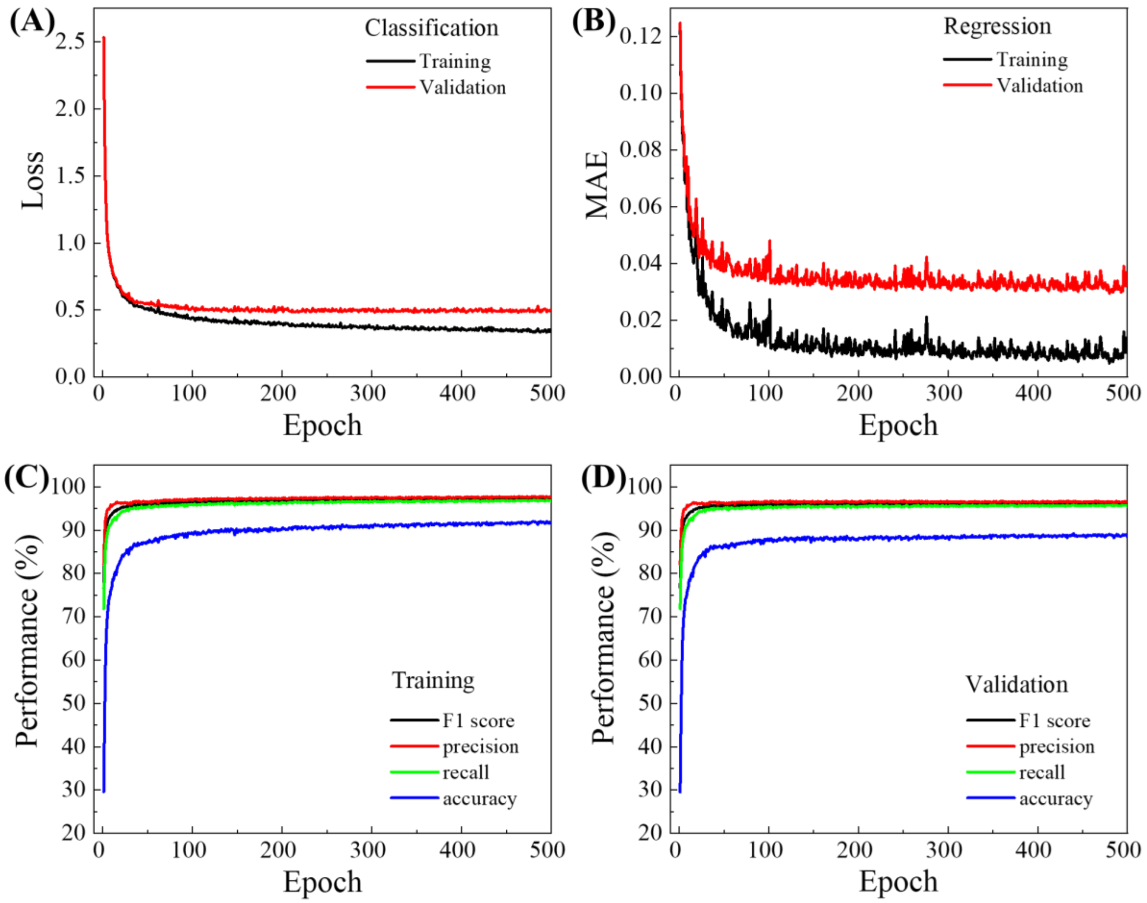

Figure S16. Training and validation loss of the MultiplexCR model with ten sequential convolutional blocks for (**A**) classification and (**B**) regression. The model's classification performances during (**C**) training and (**D**) validation, including F1 score, precision, recall, and accuracy.

Section S10. Determination of limit of detection (LOD) for virus co-infection.

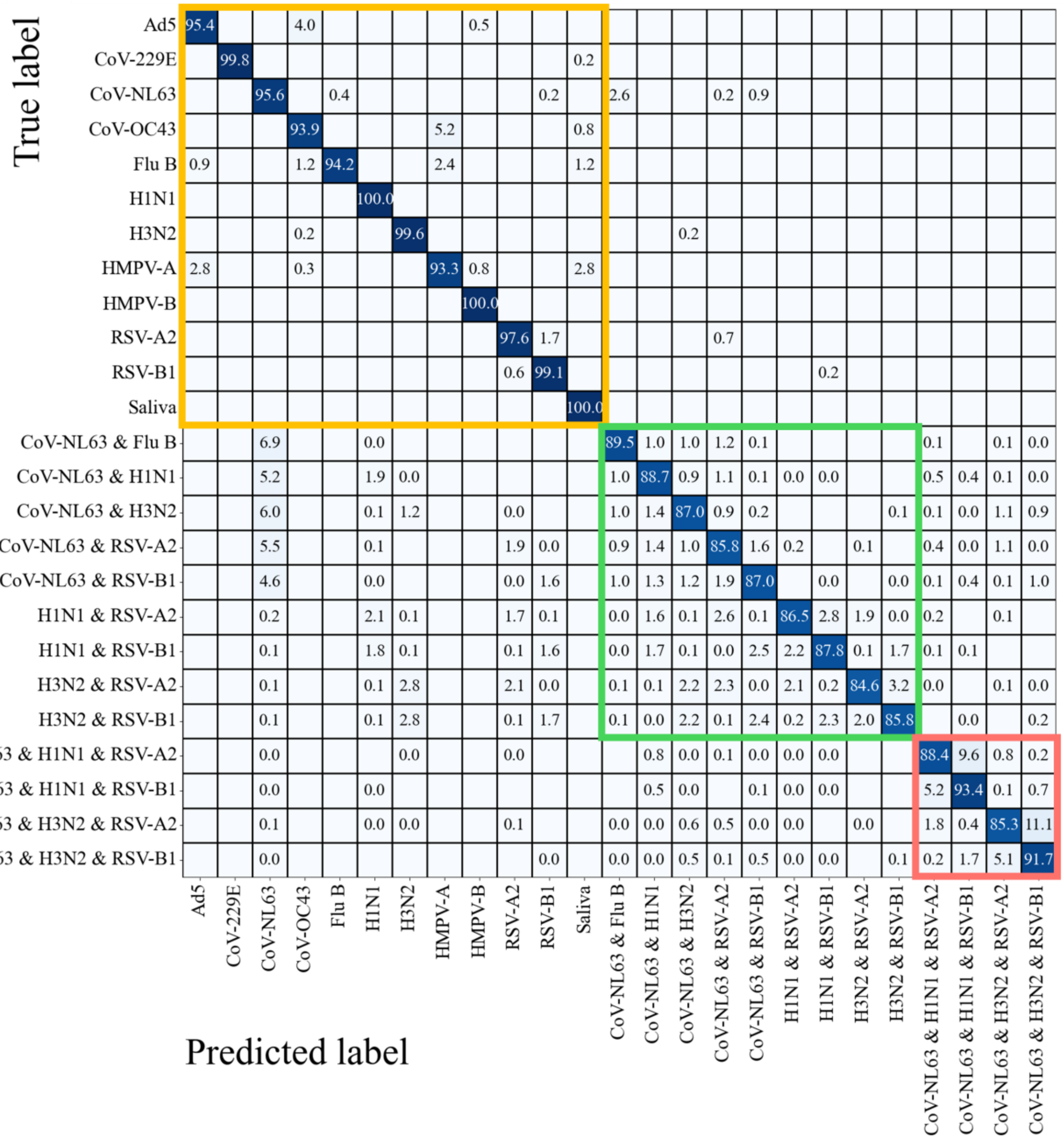

Figure S17. Confusion matrix of the MultiplexCR model trained with SERS spectra of all concentrations in experiments from eleven SVs, nine 2VMs, and four 3VMs in saliva, as well as the reference saliva. The matrix entries represent the percentage of test spectra predicted as a specific class (first row) given a ground truth of class (first column). Diagonal entries show the accuracy for each class. Note that "0.0" indicates values below 0.04%, rather than an actual zero.

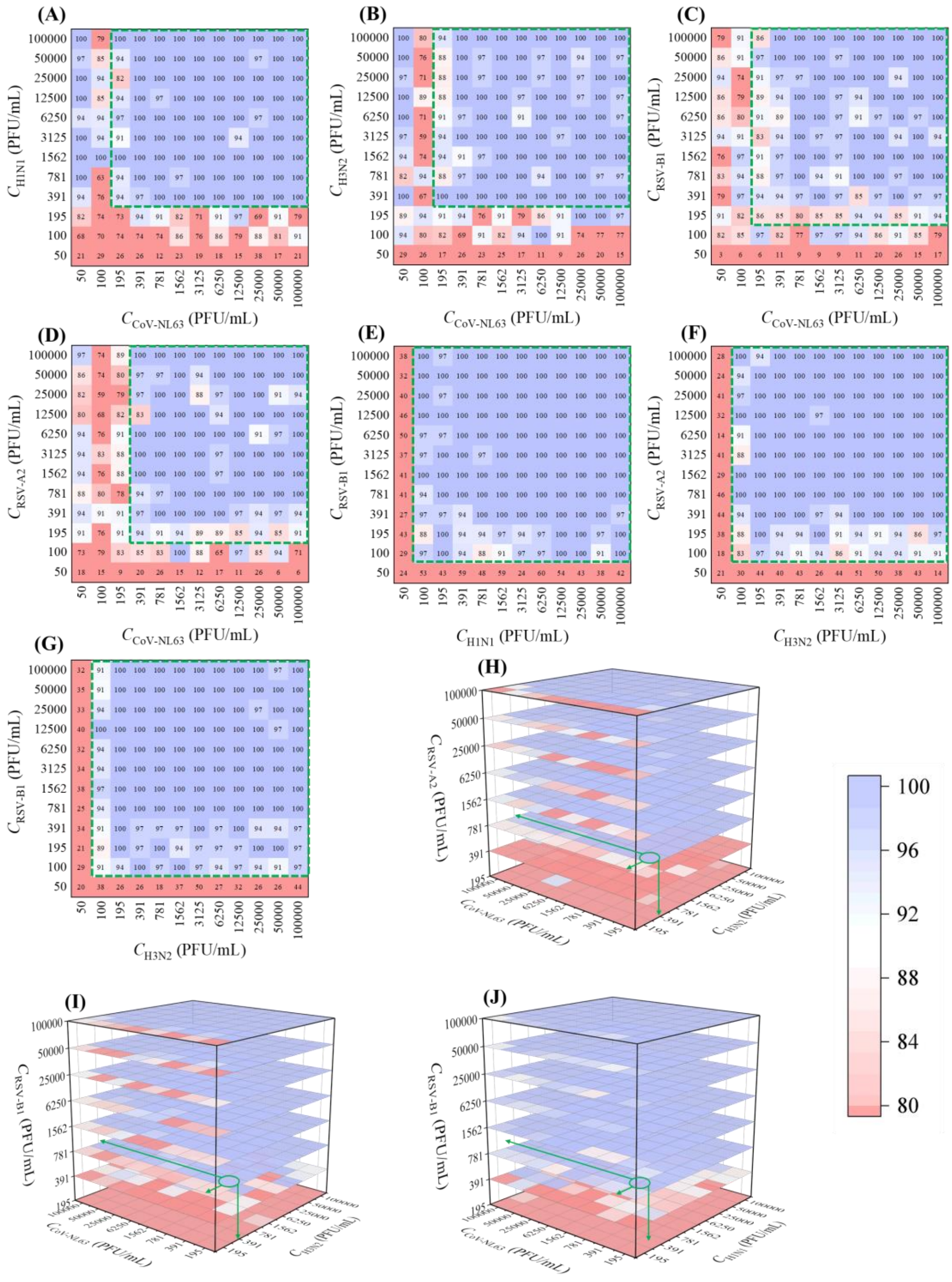

Figure S18. **The accuracy heatmaps of 2VMs and 3VMs.** (**A**) CoV-NL63 & H1N1, (**B**) CoV-NL63 & H3N2, (**C**) CoV-NL63 & RSV-B1, (**D**) CoV-NL63 & RSV-A2, (**E**) H1N1 & RSV-B1, (**F**) H3N2 & RSV-A2, (**G**) H3N2 & RSV-B1, (**H**) CoV-NL63 & H3N2 & RSV-A2, (**I**) CoV-NL63 & H3N2 & RSV-B1, and (**J**) CoV-NL63 & H1N1 & RSV-B1.

Table S5. The LOD list of different virus mixtures.

Virus species	LOD (PFU/mL)
Ad5	100
CoV-229E	50
CoV-NL63	50
CoV-OC43	100
Flu B	100
H1N1	50
H3N2	50
HMPV-A	100
HMPV-B	50
RSV-A2	50
RSV-B1	100
CoV-NL63 & Flu B	(100, 100)
CoV-NL63 & H1N1	(195, 391)
CoV-NL63 & H3N2	(195, 391)
CoV-NL63 & RSVA2	(391, 195)
CoV-NL63 & RSVB1	(195, 195)
H1N1 & RSV-A2	(100, 100)
H1N1 & RSV-B1	(100, 100)
H3N2 & RSV-A2	(100, 100)
H3N2 & RSV-B1	(100, 100)
CoV-NL63 & H1N1 & RSV-A2	(195, 195, 781)
CoV-NL63 & H1N1 & RSV-B1	(195, 195, 781)
CoV-NL63 & H3N2 & RSV-A2	(195, 391, 781)
CoV-NL63 & H3N2 & RSV-B1	(195, 391, 781)

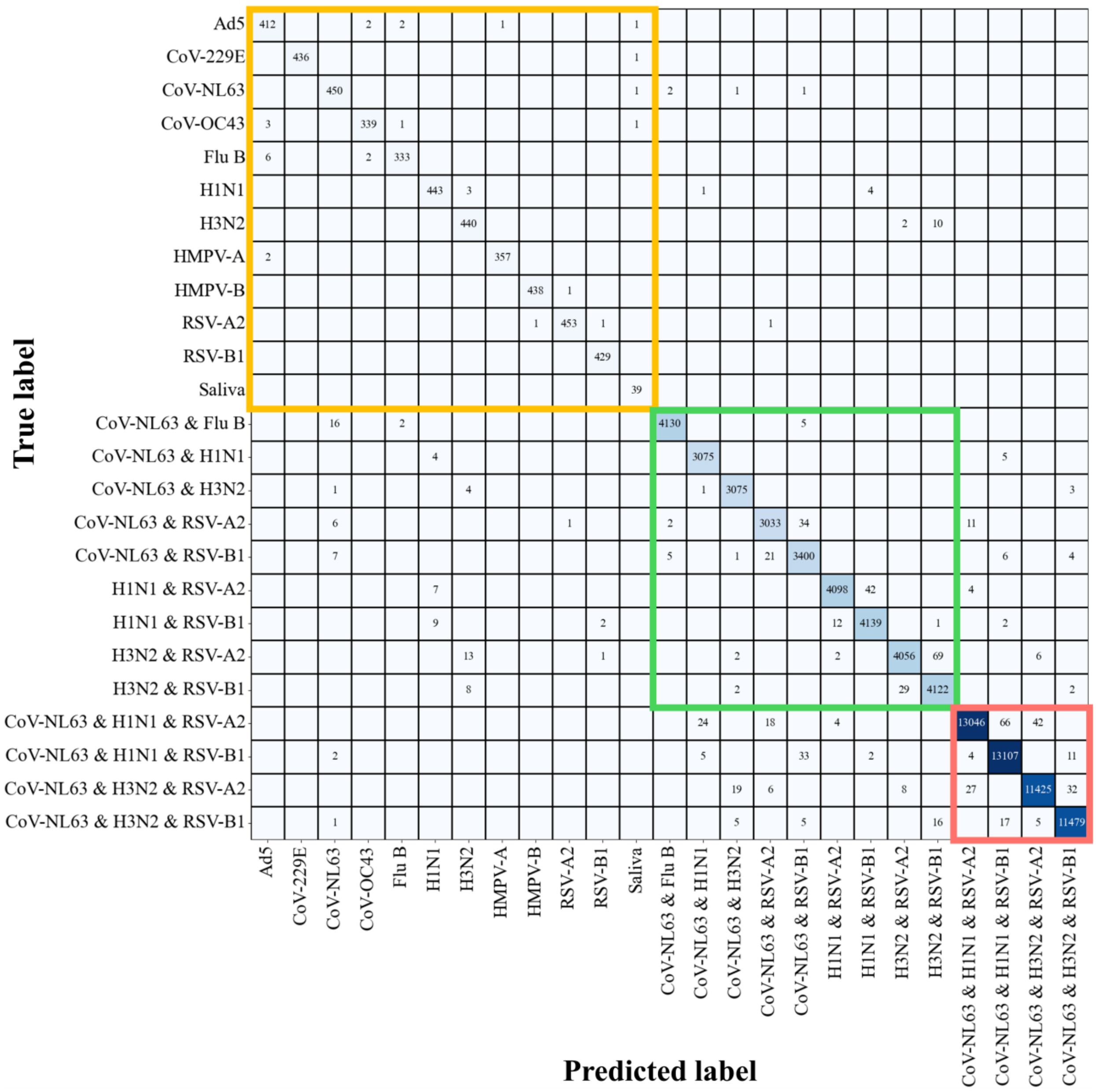

Figure S19. The overall confusion matrix displaying the number of spectra for the MultiplexCR model's detection of eleven SVs, nine 2VMs, and four 3VMs in saliva with spectra of $C_{\text{virus}} \geq$ LODs, as well as the reference saliva. The matrix entries represent the percentage of test spectra predicted as a specific class (first row) given a ground truth of class (first column). Diagonal entries show the accuracy for each class.

Section S11. Additional results for quantification of virus co-infections.

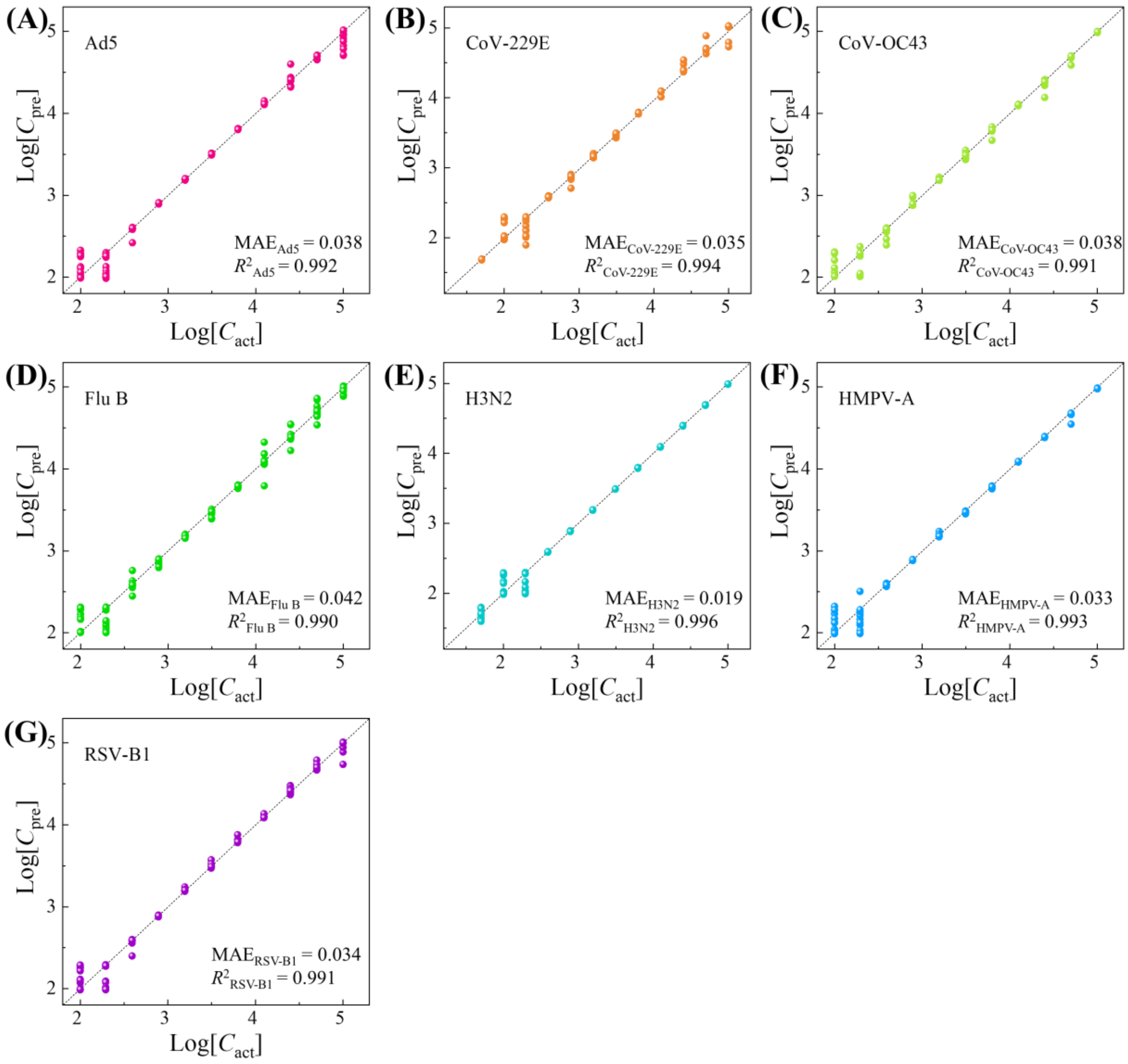

Figure S20. **Regression results of the MultiplexCR model for SV detection in saliva.** (**A**) Ad5, (**B**) CoV-229E, (**C**) CoV-OC43, (**D**) Flu B, (**E**) H3N2, (**F**) HMPV-A, and (**G**) RSV-B1. The concentration units for C_{act} and C_{pre} are in PFU/mL. The *x*-axis represents $\log_{10}C_{act}$ of testing spectra, and *y*-axis is $\log_{10}C_{pre}$. The dash line shows $\log_{10}C_{act} = \log_{10}C_{pre}$.

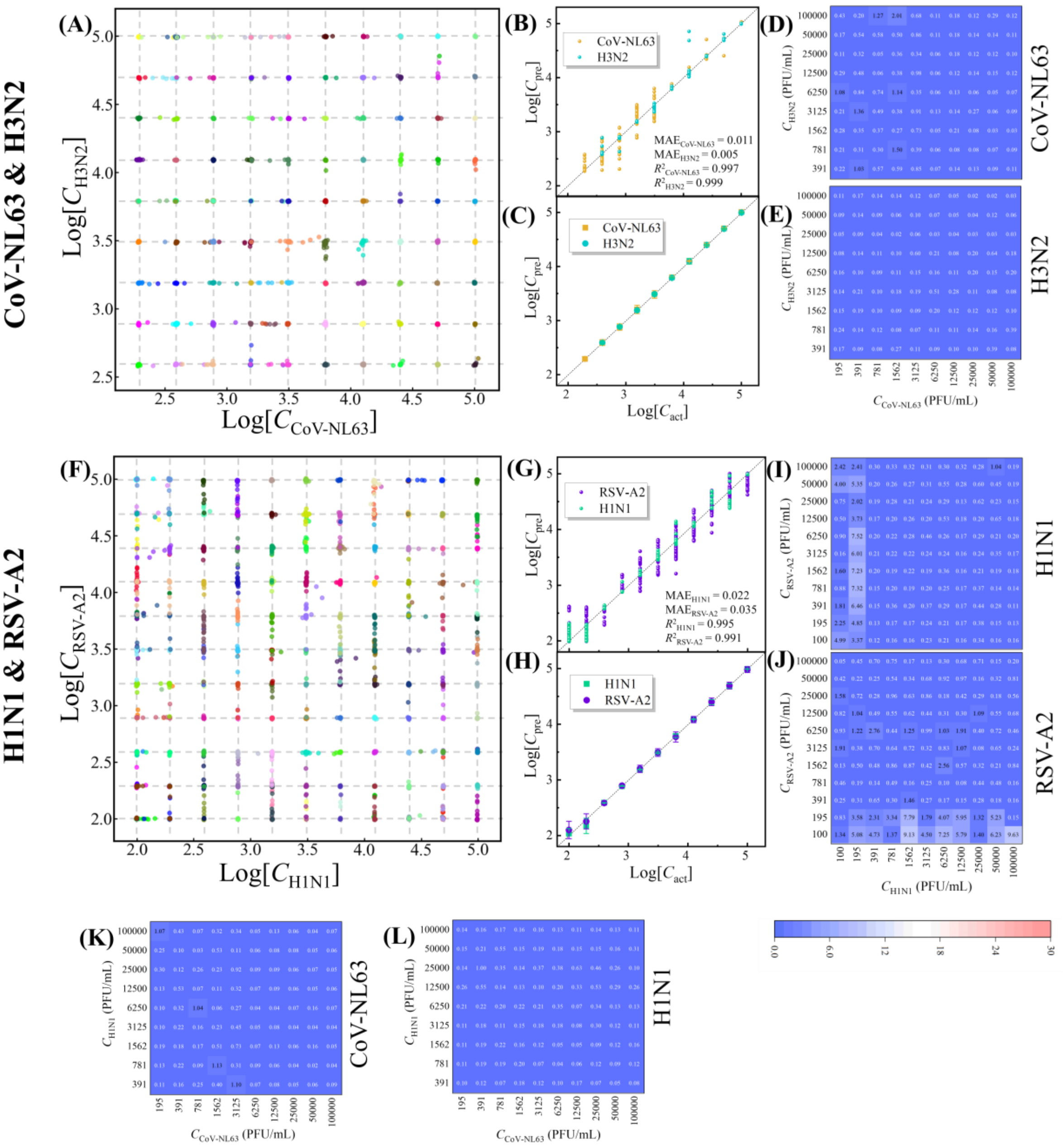

Figure S21. **Regression results from the MultiplexCR model of 2VMs in saliva. For CoV-NL63 & H3N2.** (**A**) A 2D scattered plot for concentration distributions of CoV-NL63 & H3N2, with the *x*-axis representing $\log_{10}C_{\text{CoV-NL63}}$ and *y*-axis being $\log_{10}C_{\text{H3N2}}$. (**B**) A re-plot of the regression results from (A) for CoV-NL63 (orange dots) and H3N2 (blue dots), *x*-axis is $\log_{10}C_{\text{act}}$, *y*-axis is $\log_{10}C_{\text{pre}}$. Results from different specimens with the same true virus concentrations are combined. (**C**) Variations in predicted concentrations based on (B), with the dash line representing $\log_{10}C_{\text{pre}} = \log_{10}C_{\text{act}}$. 2D heat maps of average relative error of concentration prediction under different concentration combinations: (**D**) CoV-NL63 and (**E**) H3N2. Similar plots are shown for the regression results of **H1N1 & RSV-A2** in (**F**) – (**J**). 2D heat maps of average relative error of concentration prediction under different concentration combinations of CoV-NL63 & H1N1: (**K**) CoV-NL63 and (**L**) H1N1.

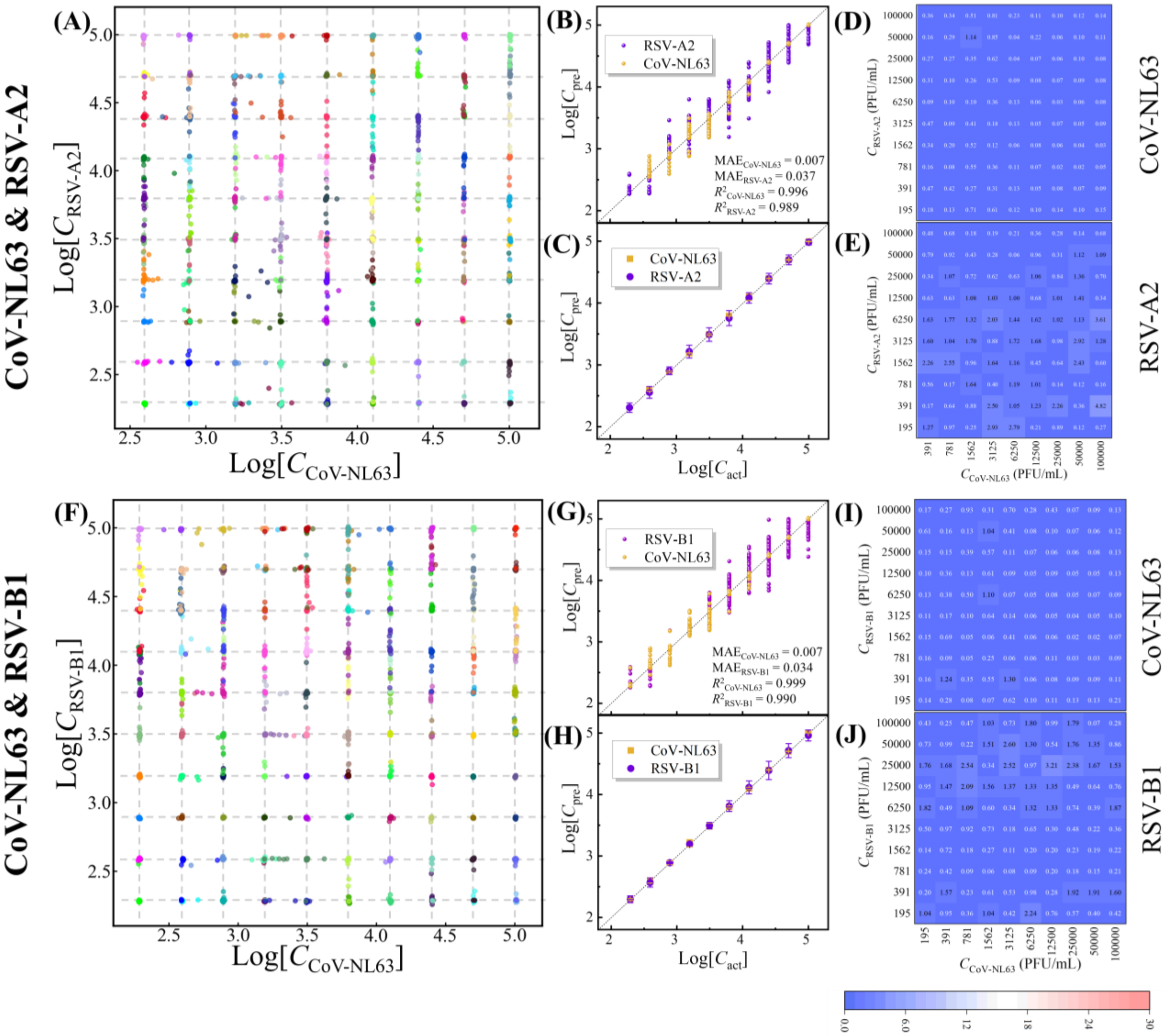

Figure S22. Regression results from the MultiplexCR model of 2VMs in saliva. For CoV-NL63 & RSV-A2. (**A**) A 2D scattered plot for concentration distributions of CoV-NL63 & Flu B, with *x*-axis representing $\log_{10}C_{CoV\text{-}NL63}$ and *y*-axis being $\log_{10}C_{RSV\text{-}A2}$. (**B**) A re-plot of the regression results from (A) for CoV-NL63 (orange dots) and RSV-A2 (purple dots), *x*-axis is $\log_{10}C_{act}$, *y*-axis is $\log_{10}C_{pre}$, results from same true virus concentrations from different specimens are combined. (**C**) Variations in predicted concentrations based on (B), with the dash line representing $\log_{10}C_{pre} = \log_{10}C_{act}$. 2D heat maps of average relative error of concentration prediction under different concentration combinations: (**D**) CoV-NL63, (**E**) RSV-A2. Similar plots are shown for the regression results of **CoV-NL63 & RSV-B1** in (**F**) – (**J**).

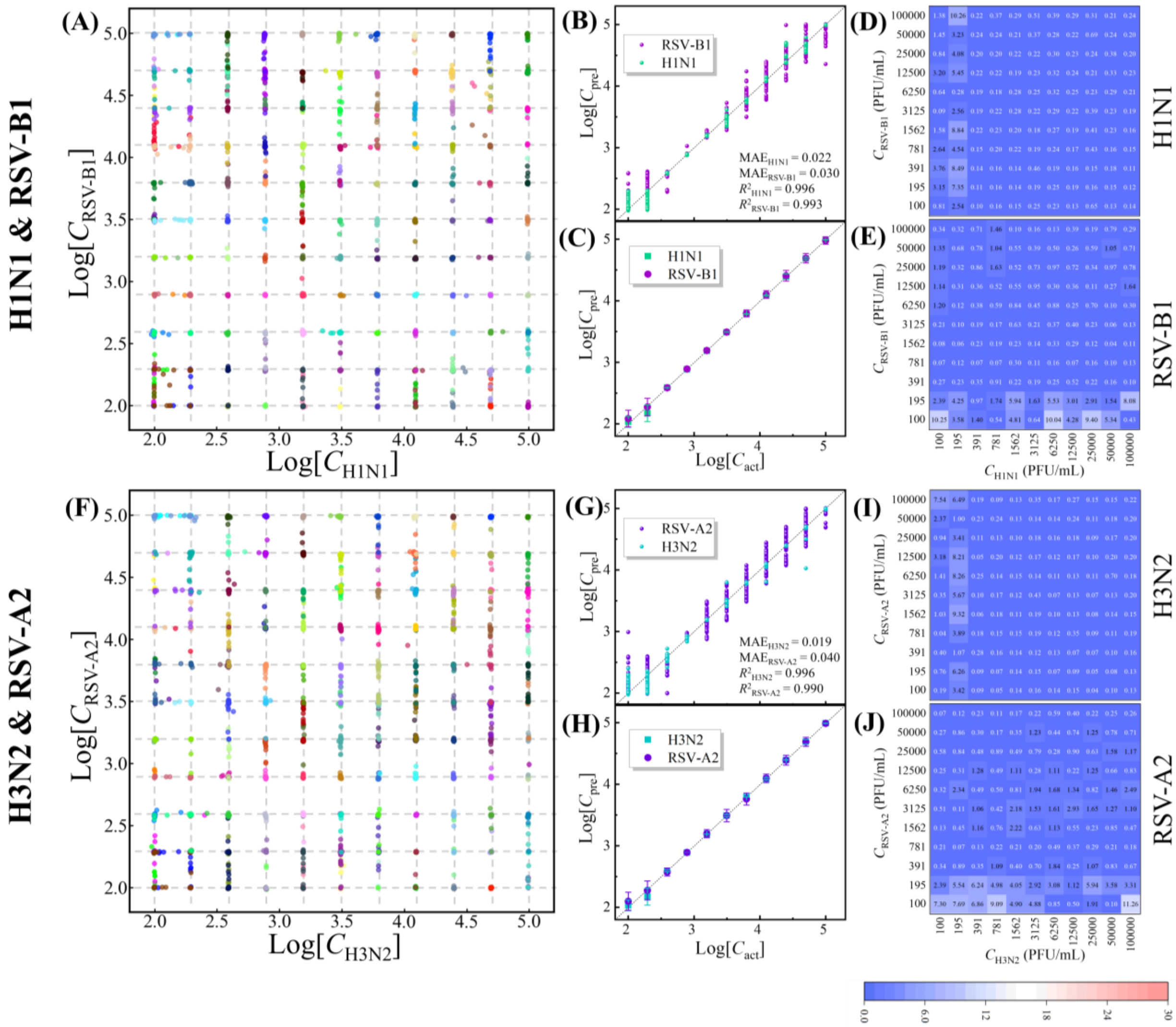

Figure S23. Regression results from the MultiplexCR model of 2VMs in saliva. For **H1N1 & RSV-B1**. (**A**) A 2D scattered plot for concentration distributions of H1N1 & RSV-B1, with the *x*-axis representing $\log_{10}C_{\text{H1N1}}$ and *y*-axis being $\log_{10}C_{\text{RSV-B1}}$. (**B**) A re-plot of the regression results from (A) for H1N1 (green dots) and RSV-B1 (light purple dots), *x*-axis is $\log_{10}C_{\text{act}}$, *y*-axis is $\log_{10}C_{\text{pre}}$, results from same true virus concentrations from different specimens are combined. (**C**) Variations in predicted concentrations based on (B), with the dash line representing $\log_{10}C_{\text{pre}} = \log_{10}C_{\text{act}}$. 2D heat maps of average relative error of concentration prediction under different concentration combinations: (**D**) H1N1 and (**E**) RSV-B1. Similar plots are shown for the regression results of **H3N2 & RSV-A2** in (**F**) – (**J**).

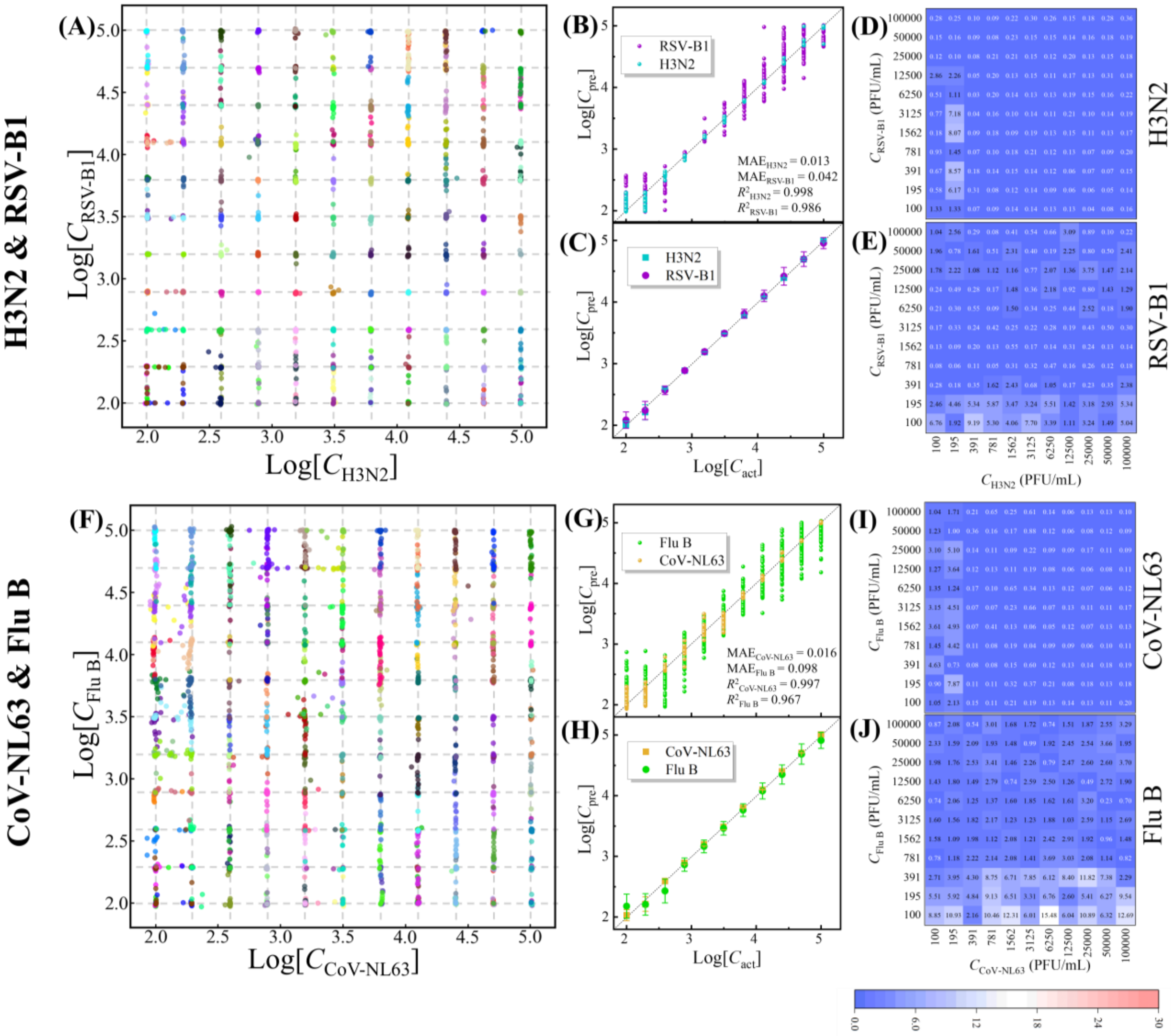

Figure S24. Regression results from the MultiplexCR model of 2VMs in saliva. For **H3N2 & RSV-B1.** (**A**) A 2D scattered plot for concentration distributions of H3N2 & RSV-B1, with the *x*-axis representing $\log_{10}C_{H3N2}$ and *y*-axis being $\log_{10}C_{RSV\text{-}B1}$. (**B**) A re-plot of the regression results from (A) for H3N2 (blue dots) and RSV-B1 (light purple dots), *x*-axis is $\log_{10}C_{act}$, *y*-axis is $\log_{10}C_{pre}$, results from same true virus concentrations from different specimens are combined. (**C**) Variations in predicted concentrations based on (B), with the dash line representing $\log_{10}C_{pre} = \log_{10}C_{act}$. 2D heat maps of average relative error of concentration prediction under different concentration combinations: (**D**) H3N2 and (**E**) RSV-B1. Similar plots are shown for the regression results of **CoV-NL63 & Flu B** in (**F**) – (**J**).

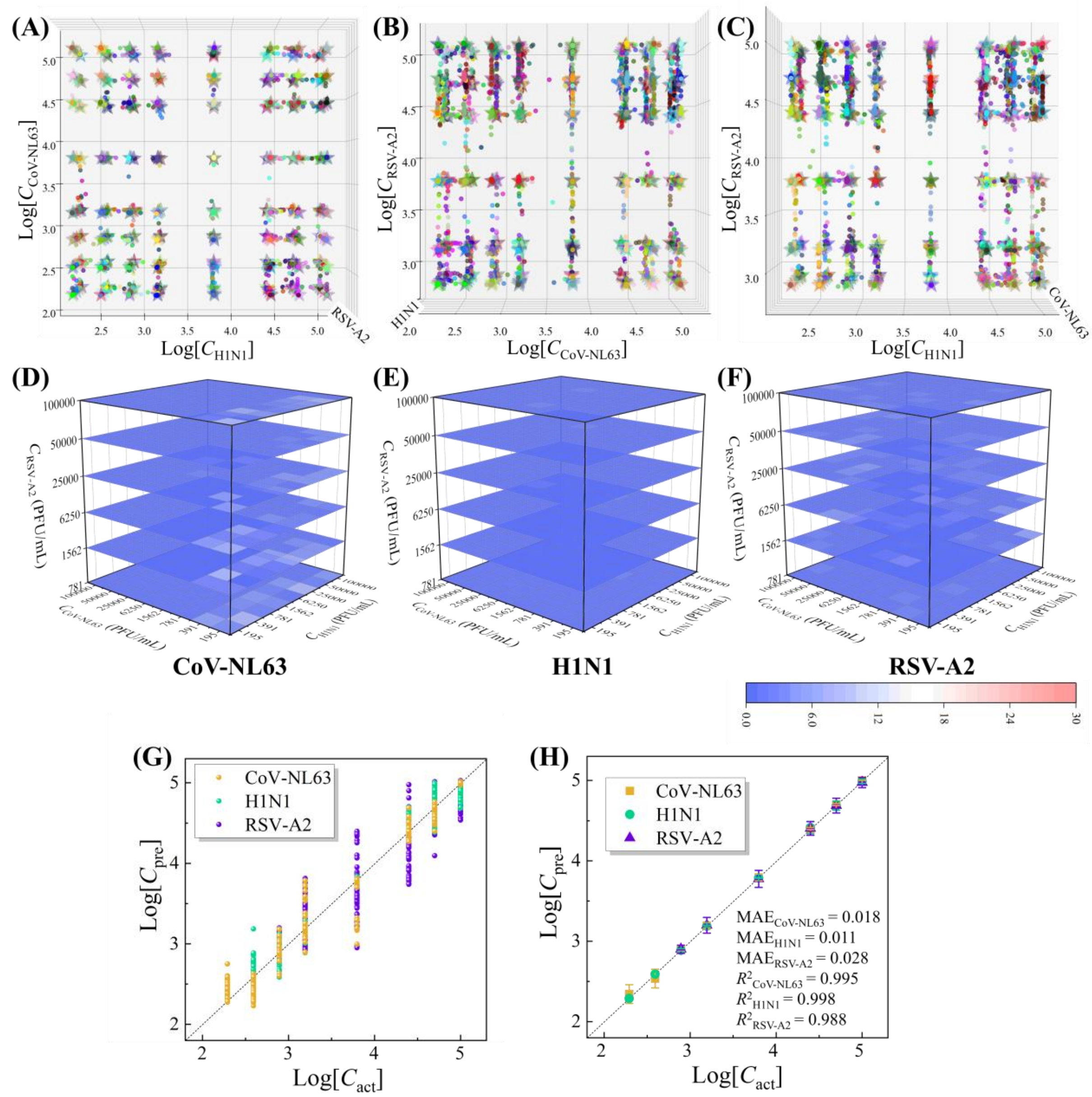

Figure S25. Regression results from the MultiplexCR model of 3VMs for CoV-NL63 & H1N1 & RSV-A2. 3D scatter plots of predicted concentrations: (**A**) the top view, (**B**) front view, (**C**) side view, with *x*-axis for $\log_{10}[C_{CoV\text{-}NL63}]$, *y*-axis for $\log_{10}[C_{H1N1}]$, and *z*-axis for $\log_{10}[C_{RSV\text{-}A2}]$. The stars with different colors represent the true virus concentration values for different concentration combinations according to the experiment design, and the corresponding predicted two concentrations for both two different viruses are plotted using dots with same colors. 3D heat maps of average relative errors of concentration predictions under different concentration combinations: (**D**) CoV-NL63, (**E**) H1N1, (**F**) RSV-A2. (**G**) A re-plot of the regression results for CoV-NL63, H1N1, and RSV-A2, *x*-axis is $\log_{10}C_{act}$, *y*-axis is $\log_{10}C_{pre}$, results from same true virus concentrations from different specimens are combined. (**H**) Variations in predicted concentrations based on (G).

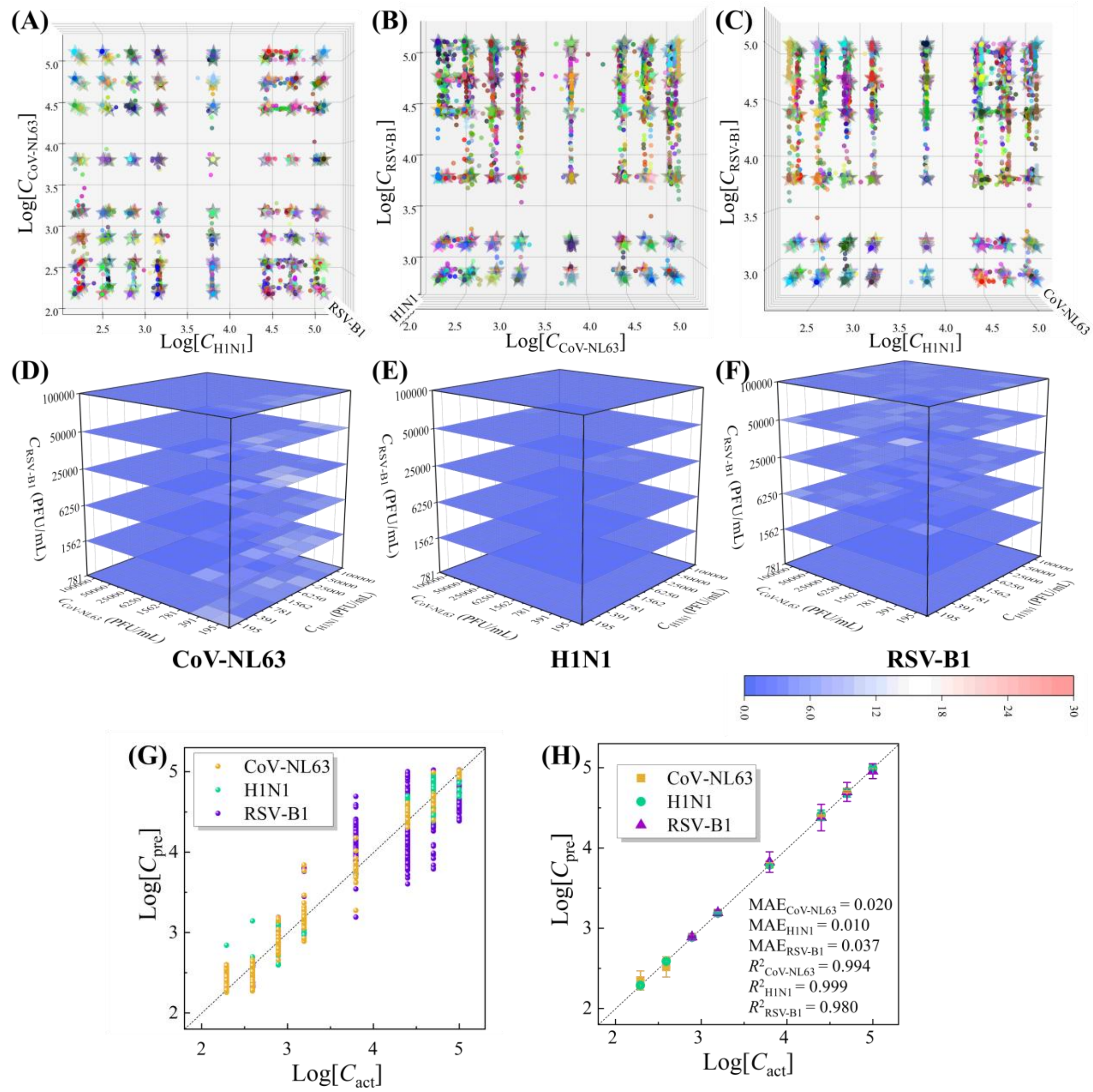

Figure S26. Regression results from the MultiplexCR model of 3VMs for CoV-NL63 & H1N1 & RSV-B1. 3D scatter plots of predicted concentrations: (**A**) the top view, (**B**) front view, (**C**) side view, with *x*-axis for $\log_{10}[C_{\text{CoV-NL63}}]$, *y*-axis for $\log_{10}[C_{\text{H1N1}}]$, and *z*-axis for $\log_{10}[C_{\text{RSV-B1}}]$. The stars with different colors represent the true virus concentration values for different concentration combinations according to the experiment design, and the corresponding predicted two concentrations for both two different viruses are plotted using dots with same colors. 3D heat maps of average relative errors of concentration predictions under different concentration combinations: (**D**) CoV-NL63, (**E**) H1N1, (**F**) RSV-B1. (**G**) A re-plot of the regression results for CoV-NL63, H1N1, and RSV-B1, *x*-axis is $\log_{10}C_{\text{act}}$, *y*-axis is $\log_{10}C_{\text{pre}}$, results from same true virus concentrations from different specimens are combined. (**H**) Variations in predicted concentrations based on (G).

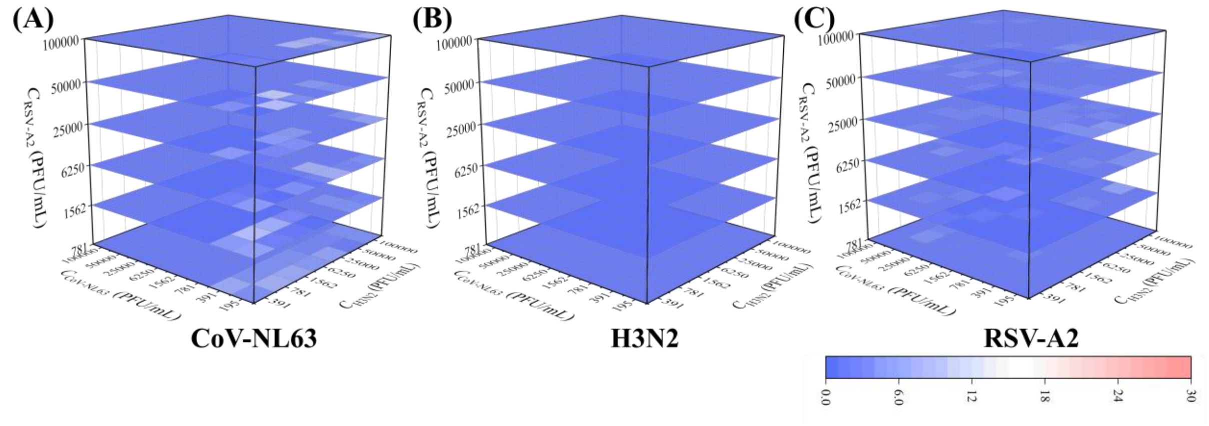

Figure S27. 3D heat maps of average relative errors of concentration predictions under different concentration combinations of CoV-NL63 & H3N2 & RSV-A2. (**A**) CoV-NL63, (**B**) H3N2, (**C**) RSV-A2.

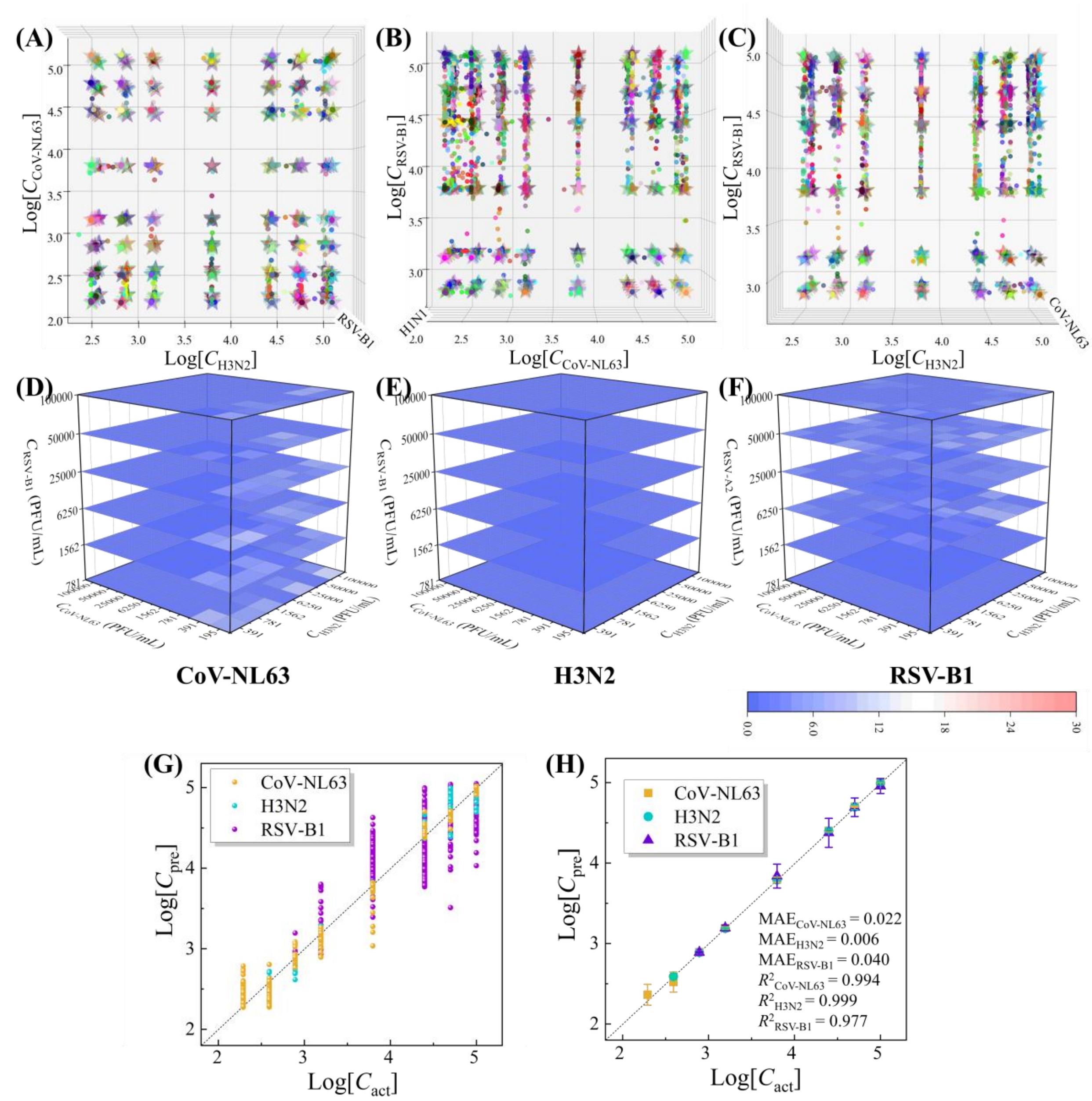

Figure S28. **Regression results from the MultiplexCR model of 3VMs for CoV-NL63 & H3N2 & RSV-B1.** 3D scatter plots of predicted concentrations: (**A**) the top view, (**B**) front view, (**C**) side view, with *x*-axis for $\log_{10}[C_{CoV-NL63}]$, *y*-axis for $\log_{10}[C_{H3N2}]$, and *z*-axis for $\log_{10}[C_{RSV-B1}]$. The stars with different colors represent the true virus concentration values for different concentration combinations according to the experiment design, and the corresponding predicted two concentrations for both two different viruses are plotted using dots with same colors. 3D heat maps of average relative error of concentration prediction under different concentration combinations: (**D**) CoV-NL63, (**E**) H3N2, (**F**) RSV-B1. (**G**) A re-plot of the regression results for CoV-NL63, H3N2, and RSV-B1, *x*-axis is $\log_{10}C_{act}$, *y*-axis is $\log_{10}C_{pre}$, results from same true virus concentrations from different specimens are combined. (**H**) Variations in predicted concentrations based on (G).

Section S12. Additional information and results for blind tests.

Table S6. Information of unknown specimens for blind tests.

Virus species	Specimen number	Concentration (PFU/mL)
Ad5	6	110, 625, 2450, 5125, 19500, 75500
CoV-229E	6	95, 825, 6200, 10500, 42500, 48000
CoV-NL63	7	85, 1260, 2850, 5500, 36000, 75800, 98000
CoV-OC43	6	180, 1250, 5525, 8500, 31000, 78000
Flu B	6	180, 1500, 5825, 19500, 39500, 72500
H1N1	5	80, 890, 3250, 23500, 95000
H3N2	5	190, 395, 6280, 10000, 75000
HMPV-A	6	120, 1180, 5500, 19800, 30000, 85000
HMPV-B	6	70, 680, 5825, 11850, 20500, 82000
RSV-A2	5	295, 5125, 23480, 45000, 89000
RSV-B1	7	125, 800, 4925, 18500, 24500, 55000, 98500
Saliva	1	
CoV-NL63 & Flu B	24	(150, 391), (150, 1562), (150, 6250), (150, 75000), (195, 75000), (580, 195), (580, 1562), (580, 75000), (781, 4688), (781, 75000), (1562, 293), (1562, 75000), (2300, 100), (2300, 6250), (2300, 100000), (6250, 4688), (50000, 293), (50000, 75000), (75000, 1562), (75000, 3125), (75000, 25000), (75000, 39062), (75000, 75000), (100000, 293)
CoV-NL63 & H1N1	22	(391, 4688), (391, 37500), (580, 391), (580, 781), (580, 1172), (580, 1562), (580, 4688), (580, 12500), (580, 25000), (580, 50000), (781, 1172), (2300, 391), (2300, 12500), (2300, 25000), (2300, 37500), (12500, 1172), (25000, 1172), (75000, 391), (75000, 3125), (75000, 4688), (75000, 6250), (75000, 100000)
CoV-NL63 & H3N2	27	(195, 9375), (391, 586), (391, 4688), (391, 39500), (580, 781), (580, 4688), (580, 25000), (580, 100000), (781, 39500), (1562, 9375), (2300, 6250), (2300, 12500), (3125, 586), (3125, 9375), (6250, 586), (6250, 4688), (12500, 9375), (12500, 39500), (25000, 4688), (25000, 9375), (50000, 586), (50000, 9375), (75000, 781), (75000, 1562), (75000, 3125), (75000, 50000), (100000, 39500)
CoV-NL63 & RSV-A2	26	(391, 72000), (580, 195), (580, 9375), (580, 50000), (580, 72000), (580, 100000), (781, 586), (1562, 9375), (2300, 3125), (2300, 9375), (2300, 50000), (2300, 72000), (3125, 586), (6250, 586), (6250, 9375), (25000, 586), (25000, 72000), (75000, 195), (75000, 3125), (75000, 6250), (75000, 25000), (75000, 50000), (75000, 72000), (75000, 100000), (100000, 9375), (100000, 72000)
CoV-NL63 & RSV-B1	34	(150, 195), (150, 12500), (150, 18750), (150, 25000), (150, 80000), (580, 781), (580, 1172), (580, 6250), (1562, 300), (1562, 1172), (1562, 18750), (1562, 80000), (2300, 300), (2300, 781), (2300, 1562), (2300, 3125), (2300, 6250), (2300, 12500), (2300, 50000), (2300, 80000), (2300, 100000), (3125, 300), (6250, 18750), (12500, 1172), (25000, 18750), (50000, 300), (75000, 1562), (75000, 6250), (75000, 18750), (75000, 25000), (75000, 80000), (75000, 100000), (100000, 300), (100000, 1172)
H1N1 & RSV-A2	37	(100, 9375), (100, 72000), (195, 586), (391, 9375), (781, 586), (781, 72000), (1172, 100), (1172, 391), (1172, 3125), (1172, 6250), (1172, 25000), (1172, 50000), (2350, 100), (2350, 391), (2350, 1562), (2350, 12500), (2350, 25000),

		(2350, 72000), (3125, 586), (4800, 195), (4800, 586), (4800, 781), (4800, 3125), (4800, 6250), (4800, 12500), (4800, 25000), (4800, 50000), (4800, 72000), (6250, 72000), (12500, 586), (25000, 586), (25000, 72000), (37500, 195), (37500, 391), (37500, 6250), (50000, 586), (100000, 9375)
H1N1 & RSV-B1	36	(195, 300), (195, 1172), (195, 80000), (391, 80000), (781, 1172), (1172, 18750), (1172, 50000), (1562, 300), (1562, 18750), (1562, 80000), (2350, 1562), (2350, 50000), (3125, 300), (3125, 1172), (4800, 100), (4800, 195), (4800, 300), (4800, 1562), (4800, 6250), (4800, 18750), (4800, 100000), (6250, 300), (6250, 1172), (6250, 18750), (12500, 80000), (25000, 300), (37500, 100), (37500, 195), (37500, 300), (37500, 391), (37500, 781), (37500, 50000), (37500, 100000), (50000, 1172), (100000, 300), (100000, 1172)
H3N2 & RSV-A2	38	(195, 586), (195, 72000), (391, 9375), (586, 100), (586, 195), (586, 12500), (586, 50000), (586, 100000), (781, 9375), (781, 72000), (1562, 586), (3125, 9375), (3125, 72000), (4688, 195), (4688, 1562), (4688, 9375), (4688, 25000), (4688, 50000), (4688, 100000), (6250, 9375), (6250, 72000), (9375, 100), (9375, 195), (9375, 1562), (9375, 3125), (9375, 12500), (9375, 25000), (9375, 50000), (9375, 100000), (12500, 72000), (25000, 586), (25000, 9375), (39500, 1562), (39500, 3125), (39500, 9375), (39500, 12500), (39500, 50000), (50000, 586)
H3N2 & RSV-B1	33	(100, 1172), (195, 18750), (391, 300), (391, 1172), (586, 391), (586, 1562), (586, 12500), (586, 18750), (781, 1172), (1562, 18750), (3125, 1172), (4688, 391), (4688, 781), (4688, 1172), (4688, 1562), (4688, 3125), (4688, 6250), (4688, 18750), (4688, 25000), (4688, 100000), (6250, 1172), (9375, 195), (9375, 391), (9375, 1172), (9375, 50000), (9375, 100000), (12500, 300), (25000, 300), (39500, 100), (39500, 3125), (39500, 25000), (50000, 80000), (100000, 300)
CoV-NL63 & H1N1 & RSV-A2	93	(195, 391, 9375), (195, 1172, 781), (195, 1172, 1562), (195, 1172, 25000), (195, 4800, 781), (195, 4800, 25000), (195, 4800, 50000), (195, 37500, 25000), (195, 50000, 9375), (391, 1172, 100000), (391, 6250, 9375), (391, 25000, 9375), (391, 37500, 1562), (391, 100000, 9375), (580, 195, 1562), (580, 195, 6250), (580, 195, 25000), (580, 391, 781), (580, 391, 1562), (580, 781, 781), (580, 1172, 781), (580, 1172, 1562), (580, 1172, 25000), (580, 1562, 6250), (580, 1562, 9375), (580, 1562, 25000), (580, 4800, 6250), (580, 6250, 50000), (580, 25000, 1562), (580, 25000, 6250), (580, 25000, 25000), (580, 25000, 50000), (580, 37500, 6250), (580, 37500, 9375), (580, 50000, 781), (580, 50000, 6250), (580, 50000, 9375), (580, 50000, 100000), (580, 100000, 9375), (580, 100000, 100000), (781, 195, 9375), (781, 391, 9375), (781, 1172, 25000), (781, 1172, 100000), (781, 1562, 9375), (781, 4800, 6250), (781, 4800, 9375), (781, 4800, 50000), (781, 4800, 100000), (781, 37500, 781), (781, 37500, 9375), (1562, 195, 9375), (1562, 1172, 1562), (1562, 4800, 781), (1562, 4800, 1562), (1562, 4800, 6250), (1562, 4800, 25000), (1562, 25000, 9375), (1562, 37500, 781), (1562, 37500, 1562), (1562, 37500, 50000), (1562, 37500, 100000), (1562, 100000, 9375), (6250, 781, 9375), (6250, 1172, 6250), (6250, 1172, 9375), (6250, 1172, 25000), (6250, 4800, 50000), (6250, 37500, 781), (6250, 37500, 50000), (25000, 1172, 1562), (25000, 6250, 9375), (25000, 37500, 1562), (25000, 37500, 9375), (25000, 37500, 50000), (25000, 37500, 100000), (25000, 50000, 9375), (50000, 391, 9375), (50000, 1172, 25000), (50000, 1172, 50000), (50000, 4800, 1562), (50000, 4800, 50000), (50000, 6250, 9375), (50000, 25000, 9375), (50000, 37500, 1562), (50000, 100000, 9375), (100000, 781, 9375), (100000, 4800, 6250), (100000, 4800, 25000),

		(100000, 37500, 781), (100000, 37500, 25000), (100000, 37500, 50000), (100000, 100000, 9375)
CoV-NL63 & H1N1 & RSV-B1	115	(195, 195, 1172), (195, 391, 1172), (195, 781, 1172), (195, 1172, 781), (195, 1172, 6250), (195, 1172, 100000), (195, 1562, 1172), (195, 4800, 1172), (195, 4800, 1562), (195, 6250, 1172), (195, 25000, 1172), (195, 37500, 1562), (195, 37500, 6250), (195, 37500, 25000), (195, 37500, 100000), (391, 1172, 781), (391, 1172, 1172), (391, 1172, 6250), (391, 1562, 1172), (391, 4800, 781), (391, 4800, 1562), (391, 4800, 6250), (391, 4800, 50000), (391, 4800, 100000), (391, 37500, 781), (391, 37500, 1562), (391, 37500, 6250), (391, 50000, 1172), (580, 195, 781), (580, 195, 50000), (580, 195, 100000), (580, 391, 781), (580, 391, 6250), (580, 391, 50000), (580, 781, 1172), (580, 781, 50000), (580, 781, 100000), (580, 1172, 1172), (580, 1172, 6250), (580, 1172, 25000), (580, 1562, 781), (580, 1562, 1562), (580, 1562, 6250), (580, 1562, 25000), (580, 1562, 50000), (580, 4800, 781), (580, 6250, 50000), (580, 6250, 100000), (580, 25000, 1172), (580, 37500, 1562), (580, 37500, 25000), (580, 37500, 100000), (580, 50000, 1172), (580, 50000, 6250), (580, 50000, 100000), (580, 100000, 1562), (580, 100000, 50000), (580, 100000, 100000), (781, 781, 1172), (781, 1172, 781), (781, 1172, 100000), (781, 1562, 1172), (781, 4800, 25000), (781, 4800, 50000), (781, 25000, 1172), (781, 37500, 6250), (781, 37500, 50000), (1562, 195, 1172), (1562, 1172, 1562), (1562, 1172, 6250), (1562, 4800, 50000), (1562, 4800, 100000), (1562, 37500, 1172), (1562, 37500, 25000), (1562, 37500, 100000), (1562, 50000, 1172), (1562, 100000, 1172), (6250, 781, 1172), (6250, 1172, 100000), (6250, 1562, 1172), (6250, 4800, 781), (6250, 4800, 6250), (6250, 4800, 50000), (6250, 4800, 100000), (6250, 6250, 1172), (6250, 25000, 1172), (6250, 37500, 1172), (6250, 37500, 25000), (6250, 37500, 100000), (25000, 1172, 1562), (25000, 1562, 1172), (25000, 4800, 1172), (25000, 4800, 25000), (25000, 37500, 6250), (25000, 37500, 100000), (25000, 100000, 1172), (50000, 195, 1172), (50000, 1172, 781), (50000, 1172, 1562), (50000, 4800, 25000), (50000, 4800, 50000), (50000, 37500, 781), (50000, 37500, 6250), (50000, 37500, 50000), (50000, 100000, 1172), (100000, 391, 1172), (100000, 1172, 781), (100000, 1172, 50000), (100000, 1172, 100000), (100000, 1562, 1172), (100000, 4800, 6250), (100000, 4800, 100000), (100000, 25000, 1172), (100000, 37500, 781), (100000, 37500, 1172)
CoV-NL63 & H3N2 & RSV-A2	84	(195, 586, 781), (195, 586, 1562), (195, 586, 9375), (195, 586, 25000), (195, 586, 50000), (195, 586, 100000), (195, 781, 9375), (195, 1562, 9375), (195, 9375, 6250), (195, 9375, 9375), (195, 9375, 25000), (195, 9375, 100000), (195, 25000, 9375), (391, 391, 9375), (391, 586, 6250), (391, 586, 9375), (391, 586, 25000), (391, 586, 50000), (391, 781, 9375), (391, 1562, 9375), (391, 6250, 9375), (391, 9375, 781), (391, 9375, 1562), (391, 9375, 50000), (580, 391, 9375), (580, 391, 25000), (580, 586, 9375), (580, 586, 100000), (580, 781, 6250), (580, 781, 9375), (580, 1562, 781), (580, 1562, 1562), (580, 1562, 25000), (580, 6250, 781), (580, 6250, 9375), (580, 6250, 25000), (580, 6250, 100000), (580, 9375, 781), (580, 9375, 1562), (580, 9375, 6250), (580, 9375, 25000), (580, 9375, 100000), (580, 25000, 1562), (580, 25000, 50000), (580, 50000, 6250), (580, 100000, 1562), (580, 100000, 100000), (781, 586, 781), (781, 586, 6250), (781, 586, 9375), (781, 586, 25000), (781, 586, 100000), (781, 1562, 9375), (781, 6250, 9375), (781, 9375, 6250), (781, 9375, 50000), (1562, 391, 9375), (1562, 586, 25000), (1562, 6250, 9375), (1562, 9375, 6250), (1562, 9375, 25000), (6250, 586, 25000), (6250, 586, 100000), (6250, 9375, 781), (6250, 9375, 25000), (25000, 391, 9375), (25000, 586, 781), (25000, 586, 9375), (25000, 586, 50000), (25000, 781, 9375), (25000, 1562, 9375), (25000,

		9375, 1562), (25000, 9375, 9375), (50000, 391, 9375), (50000, 586, 781), (50000, 586, 25000), (50000, 586, 50000), (50000, 9375, 781), (50000, 9375, 6250), (50000, 9375, 25000), (50000, 9375, 50000), (100000, 586, 781), (100000, 586, 25000), (100000, 586, 50000)
CoV-NL63 & H3N2 & RSV-B1	99	(195, 586, 781), (195, 781, 1172), (195, 1562, 1172), (195, 9375, 781), (195, 9375, 1172), (195, 9375, 50000), (195, 25000, 1172), (195, 50000, 1172), (195, 100000, 1172), (391, 586, 781), (391, 586, 1172), (391, 586, 100000), (391, 9375, 50000), (580, 391, 781), (580, 391, 1172), (580, 391, 1562), (580, 391, 6250), (580, 391, 50000), (580, 391, 100000), (580, 586, 1562), (580, 586, 6250), (580, 781, 1562), (580, 781, 6250), (580, 781, 25000), (580, 1562, 50000), (580, 1562, 100000), (580, 6250, 781), (580, 6250, 1562), (580, 9375, 781), (580, 9375, 25000), (580, 9375, 50000), (580, 25000, 781), (580, 25000, 100000), (580, 50000, 781), (580, 50000, 1562), (580, 50000, 6250), (580, 50000, 50000), (580, 100000, 50000), (580, 100000, 100000), (781, 391, 1172), (781, 586, 781), (781, 586, 6250), (781, 586, 25000), (781, 586, 50000), (781, 586, 100000), (781, 6250, 1172), (781, 9375, 1172), (781, 9375, 6250), (781, 9375, 25000), (781, 9375, 50000), (781, 100000, 1172), (1562, 586, 25000), (1562, 586, 100000), (1562, 781, 1172), (1562, 1562, 1172), (1562, 9375, 1172), (1562, 9375, 50000), (1562, 50000, 1172), (6250, 391, 1172), (6250, 586, 1562), (6250, 586, 6250), (6250, 6250, 1172), (6250, 9375, 781), (6250, 9375, 25000), (6250, 9375, 50000), (6250, 9375, 100000), (6250, 25000, 1172), (6250, 50000, 1172), (25000, 391, 1172), (25000, 586, 6250), (25000, 586, 25000), (25000, 586, 50000), (25000, 9375, 1172), (25000, 9375, 1562), (25000, 9375, 6250), (25000, 9375, 25000), (25000, 9375, 50000), (25000, 25000, 1172), (50000, 586, 781), (50000, 586, 25000), (50000, 586, 50000), (50000, 1562, 1172), (50000, 6250, 1172), (50000, 9375, 781), (50000, 9375, 1172), (50000, 9375, 1562), (50000, 9375, 25000), (50000, 9375, 100000), (100000, 586, 1172), (100000, 586, 1562), (100000, 586, 6250), (100000, 586, 25000), (100000, 586, 100000), (100000, 781, 1172), (100000, 6250, 1172), (100000, 9375, 781), (100000, 9375, 6250), (100000, 9375, 25000), (100000, 100000, 1172)

Table S7. Comparison of performances on the training, validation, and test spectral sets (with SERS spectra of $C_{virus} \geq$ LODs) from 5 independent trials of the MultiplexCR model.

	**Models**	**Accuracy (%)**	**F1 score (%)**	**Precision (%)**	**Recall (%)**	**MAE**
**Train**	**1**	99.54	99.89	99.95	99.83	0.0054
	**2**	99.87	99.97	99.97	99.97	0.0044
	**3**	99.39	99.85	99.92	99.78	0.0050
	**4**	99.79	99.94	99.93	99.94	0.0055
	**5**	99.85	99.96	99.95	99.97	0.0066
	**Average**	**99.7 ± 0.2**	**99.92 ± 0.05**	**99.94 ± 0.02**	**99.89 ± 0.09**	**0.0054 + 0.0008**
**Validation**	**1**	98.94	99.69	99.76	99.61	0.0214
	**2**	98.71	99.64	99.73	99.55	0.0227
	**3**	98.59	99.60	99.70	99.50	0.0278
	**4**	98.68	99.63	99.73	99.53	0.0222
	**5**	98.72	99.64	99.73	99.56	0.0257
	**Average**	**98.7 ± 0.2**	**99.64 ± 0.04**	**99.73 ± 0.03**	**99.55 ± 0.04**	**0.024 + 0.003**
**Test**	**1**	98.77	99.67	99.62	99.72	0.0250
	**2**	98.95	99.69	99.71	99.67	0.0239
	**3**	98.54	99.60	99.68	99.52	0.0306
	**4**	98.35	99.52	99.60	99.43	0.0268
	**5**	98.33	99.55	99.52	99.59	0.0325
	**Average**	**98.6 ± 0.3**	**99.61 ± 0.08**	**99.63 ± 0.08**	**99.6 ± 0.2**	**0.028 + 0.004**
**Blind test**	**1**	95.57	98.89	99.20	98.58	0.0509
	**2**	95.93	99.05	99.37	98.74	0.0431
	**3**	96.56	99.15	99.43	98.87	0.0358
	**4**	94.88	98.73	99.10	98.36	0.0464
	**5**	95.81	99.04	99.50	98.58	0.0411
	**Average**	**95.8 ± 0.6**	**99.0 ± 0.2**	**99.3 ± 0.2**	**98.6 ± 0.2**	**0.043 + 0.006**

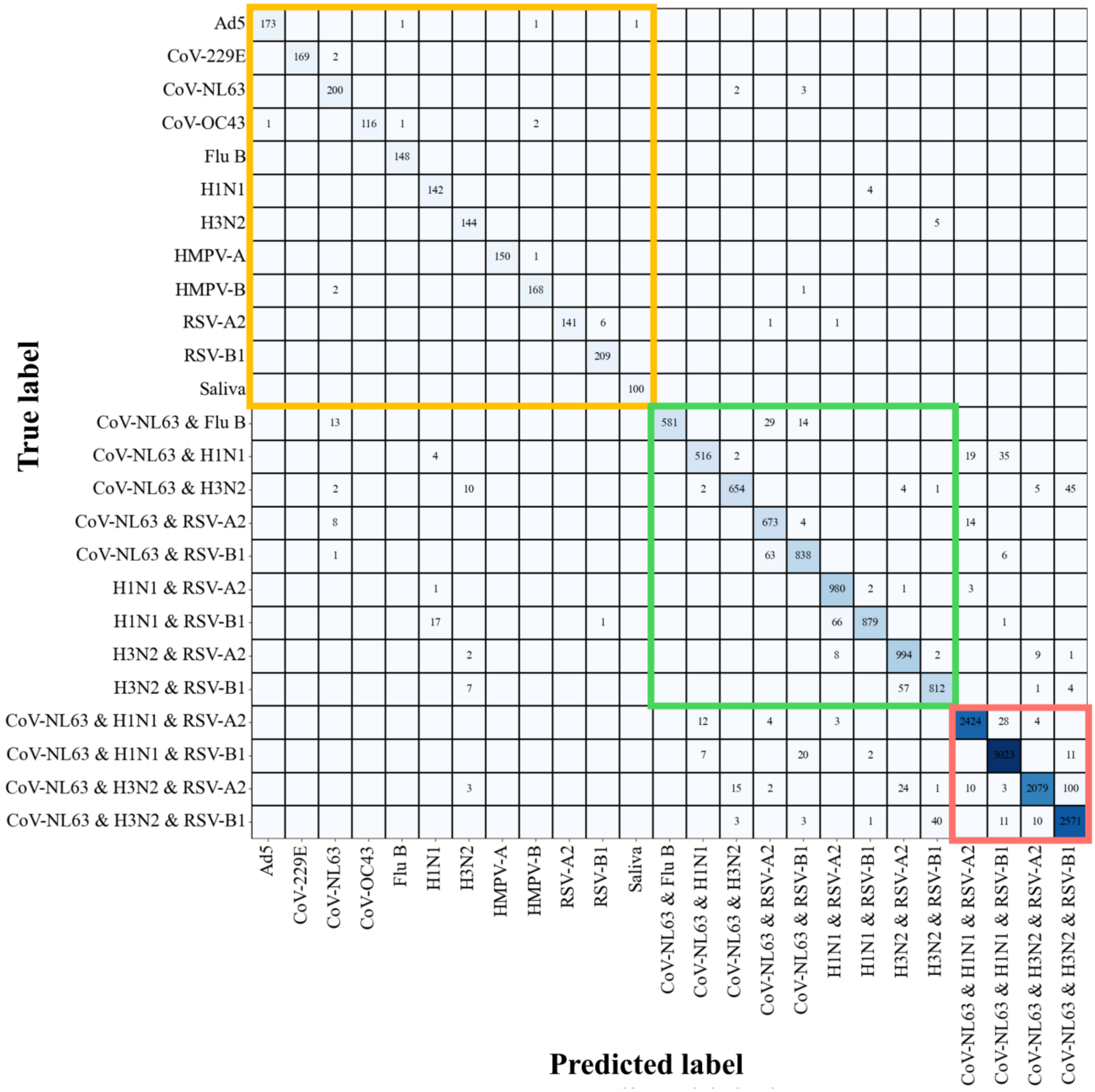

Figure S29. Blind test: The overall confusion matrix displaying the number of spectra for the MultiplexCR model's detection of eleven SVs, nine 2VMs, and four 3VMs in saliva with spectra of $C_{\text{virus}} \geq$ LODs, as well as reference saliva. Matrix entries represent the percentage of test spectra predicted as a given class (first row) based on the ground truth class (first column). Diagonal entries reflect the accuracy of each class.

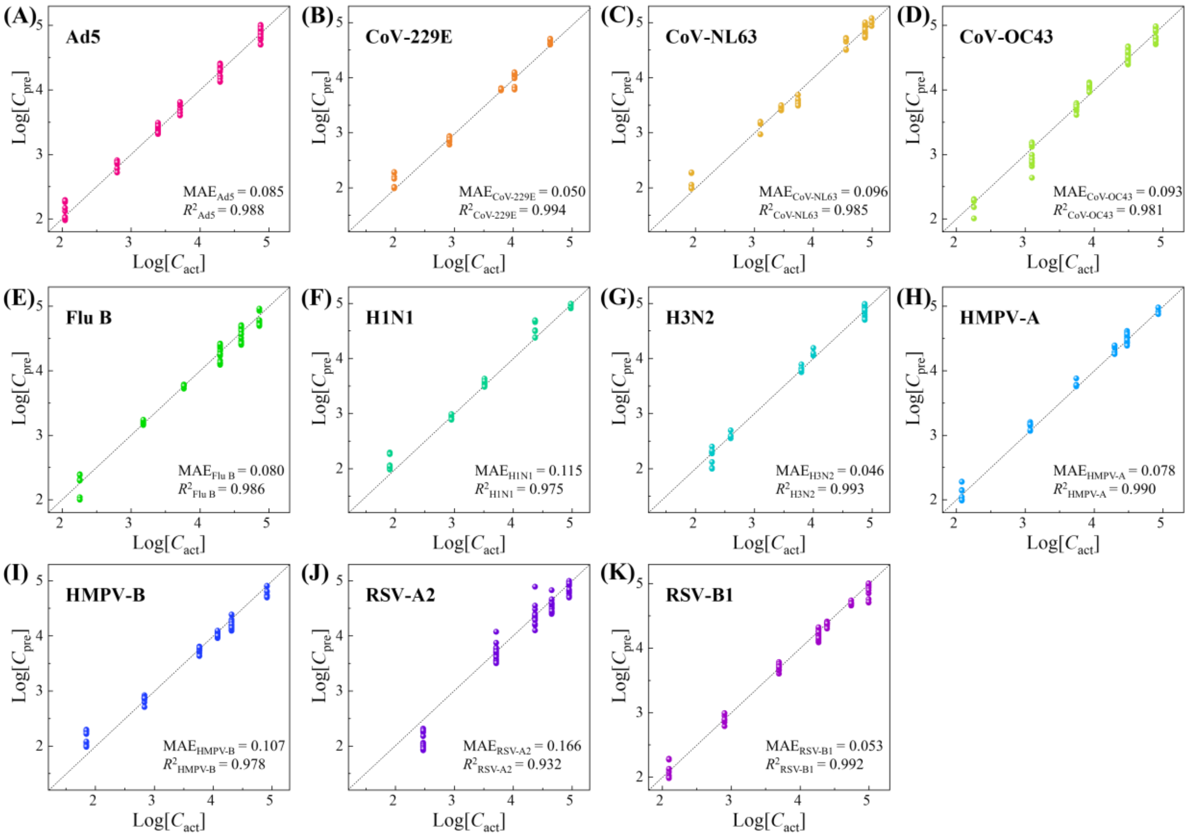

Figure S30. Regression results of the MultiplexCR model for SV detections in blind tests. (**A**) Ad5, (**B**) CoV-229E, (**C**) CoV-NL63, (**D**) CoV-OC43, (**E**) Flu B, (**F**) H1N1, (**G**) H3N2, (**H**) HMPV-A, (**I**) HMPV-B, (**J**) RSV-A2, and (**K**) RSV-B1. The *x*-axis represents $\log_{10}C_{act}$ of testing spectra, and *y*-axis is $\log_{10}C_{pre}$. The dash line shows $\log_{10}C_{act} = \log_{10}C_{pre}$.

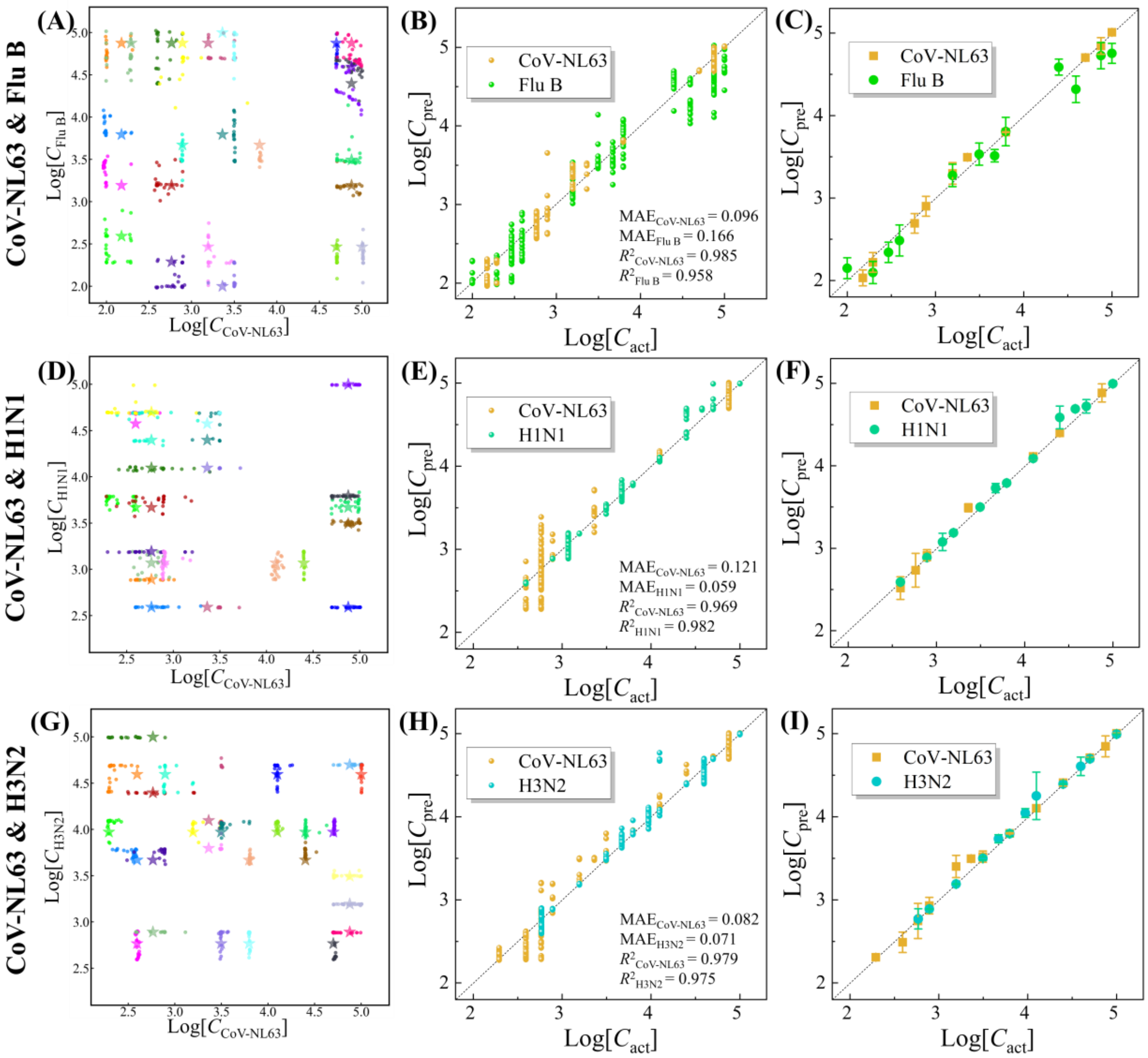

Figure S31. Regression results from the MultiplexCR model for 2VMs in blind tests. CoV-NL63 & Flu B. (**A**) A 2D scatter plot showing concentration distributions of CoV-NL63 & Flu B, with the x-axis representing $\log_{10}C_{\text{CoV-NL63}}$ and the y-axis being $\log_{10}C_{\text{Flu B}}$. Stars of different colors indicate the true virus concentration values for different concentration combinations according to the experiment design, and the corresponding predicted concentrations for both viruses are represented by dots of the same colors. (**B**) A re-plot of the regression results from (A) for CoV-NL63 (orange dots) and Flu B (green dots). Results from different specimens with the same true virus concentrations are combined. (**C**) Variations in predicted concentrations based on (B), with the dash line representing $\log_{10}C_{\text{pre}} = \log_{10}C_{\text{act}}$. Similar plots are shown for the regression results of **CoV-NL63 & H1N1** in (**D**) – (**F**), and **CoV-NL63 & H3N2** in (**G**) – (**I**).

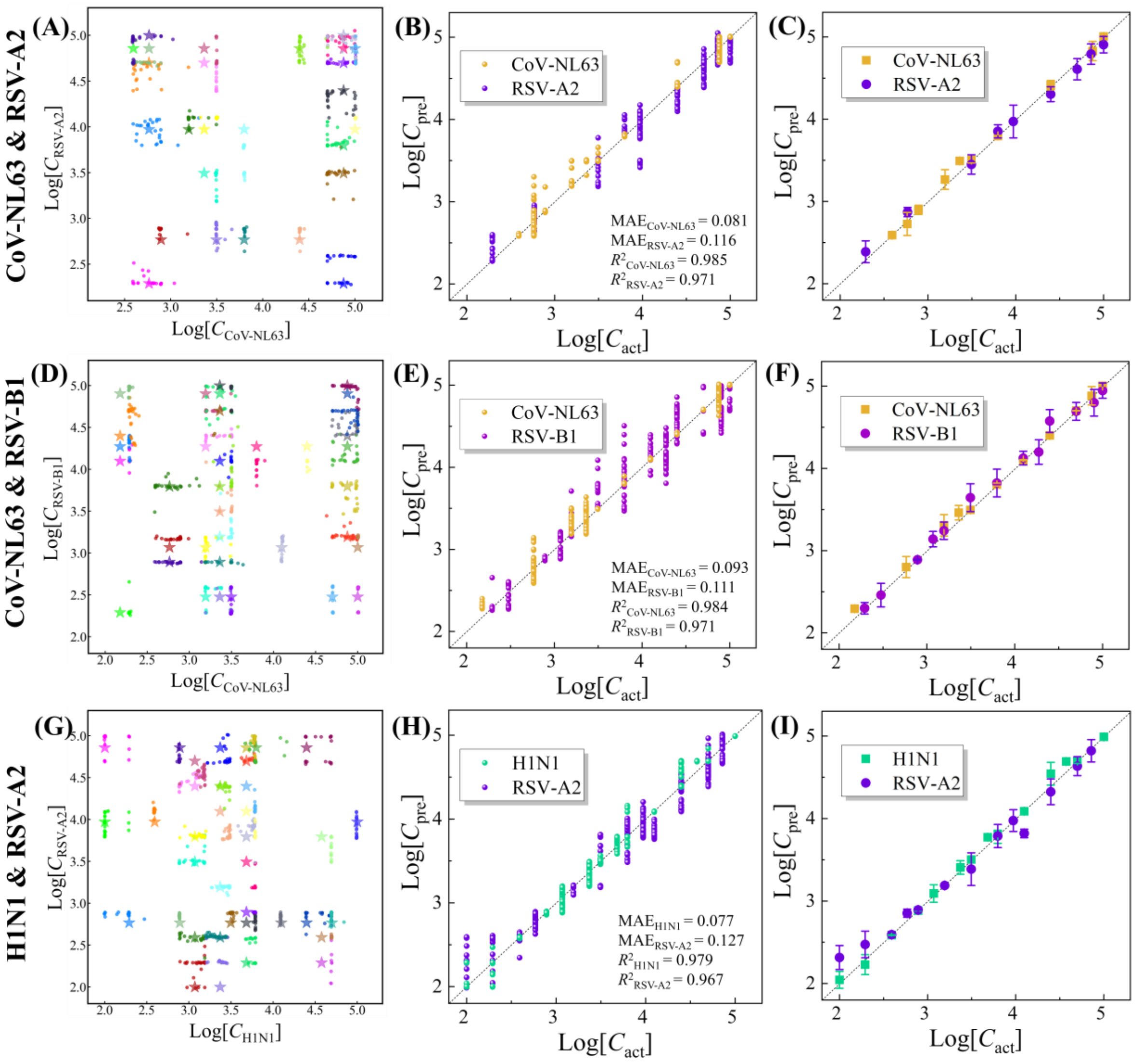

Figure S32. Regression results from the MultiplexCR model for 2VMs in blind tests. CoV-NL63 & RSV-A2. (**A**) A 2D scatter plot showing concentration distributions of CoV-NL63 & RSV-A2, with the *x*-axis representing $\log_{10}C_{CoV\text{-}NL63}$ and *y*-axis being $\log_{10}C_{RSV\text{-}A2}$. Stars of different colors indicate the true virus concentration values for different concentration combinations according to the experiment design, and the corresponding predicted concentrations for both viruses are represented by dots of the same colors. (**B**) A re-plot of the regression results from (A) for CoV-NL63 (orange dots) and RSV-A2 (purple dots). Results from different specimens with the same true virus concentrations are combined. (**C**) Variations in predicted concentrations based on (B), with the dash line representing $\log_{10}C_{pre} = \log_{10}C_{act}$. Similar plots are shown for the regression results of **CoV-NL63 & RSV-B1** in (**D**) – (**F**), and **H1N1 & RSV-A2** in (**G**) – (**I**).

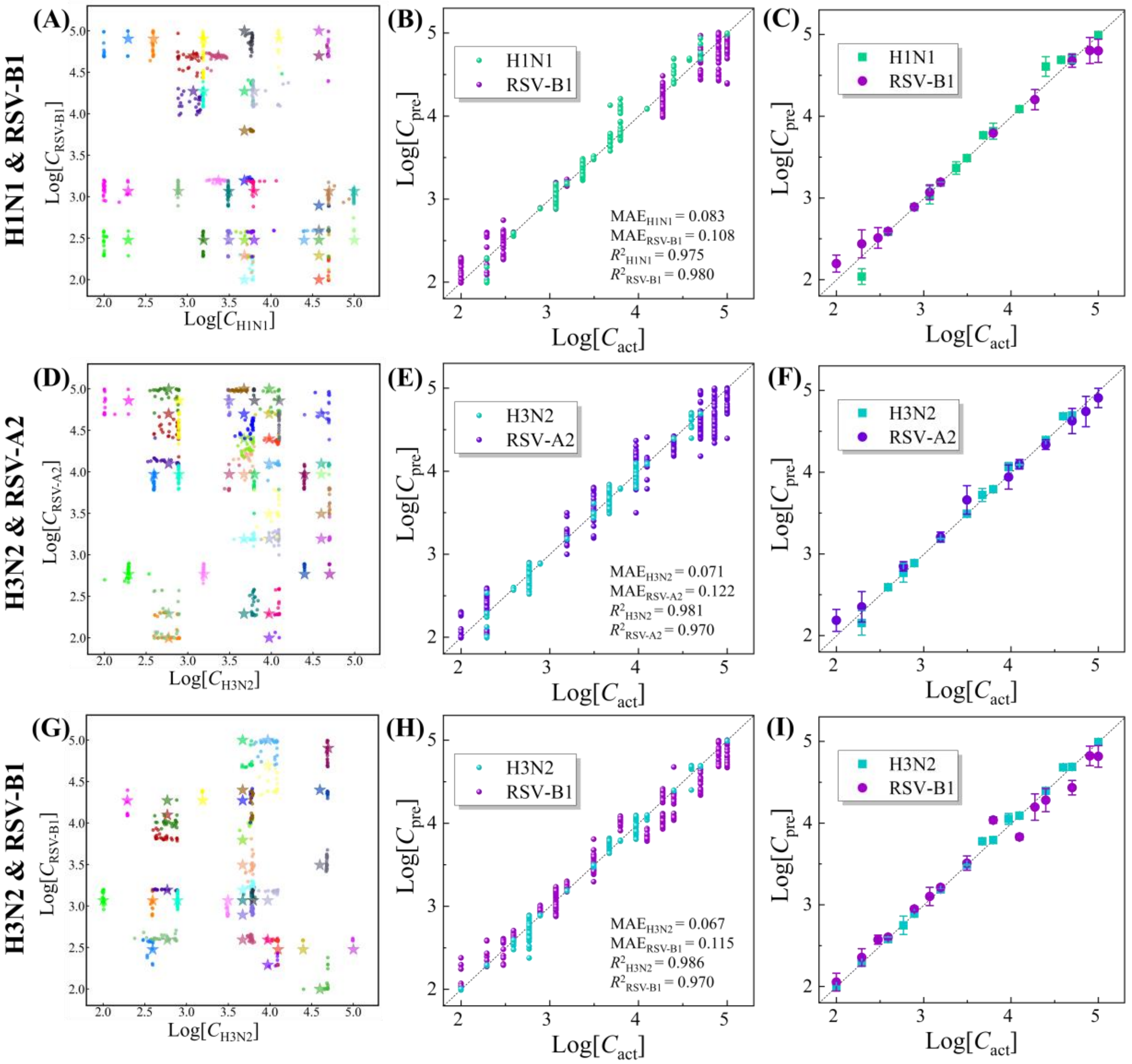

Figure S33. **Regression results from the MultiplexCR model for 2VMs in blind tests. H1N1 & RSV-B1.** (**A**) A 2D scatter plot showing concentration distributions of H1N1 & RSV-B1, with the *x*-axis representing $\log_{10}C_{\mathrm{H1N1}}$ and *y*-axis being $\log_{10}C_{\mathrm{RSV\text{-}B1}}$. Stars of different colors indicate the true virus concentration values for different concentration combinations according to the experiment design, and the corresponding predicted concentrations for both viruses are represented by dots of the same colors. (**B**) A re-plot of the regression results from (A) for H1N1 (blue-green dots) and RSV-B1 (light-purple dots), *x*-axis is actual concentration $\log_{10}C_{\mathrm{act}}$, *y*-axis is the predicted concentration $\log_{10}C_{\mathrm{pre}}$. Results from different specimens with the same true virus concentrations are combined. (**C**) Variations in predicted concentrations based on (B), with the dash line representing $\log_{10}C_{\mathrm{pre}} = \log_{10}C_{\mathrm{act}}$. Similar plots are shown for the regression results of **H3N2 & RSV-A2** in (**D**) – (**F**), and **H3N2 & RSV-B1** in (**G**) – (**I**).

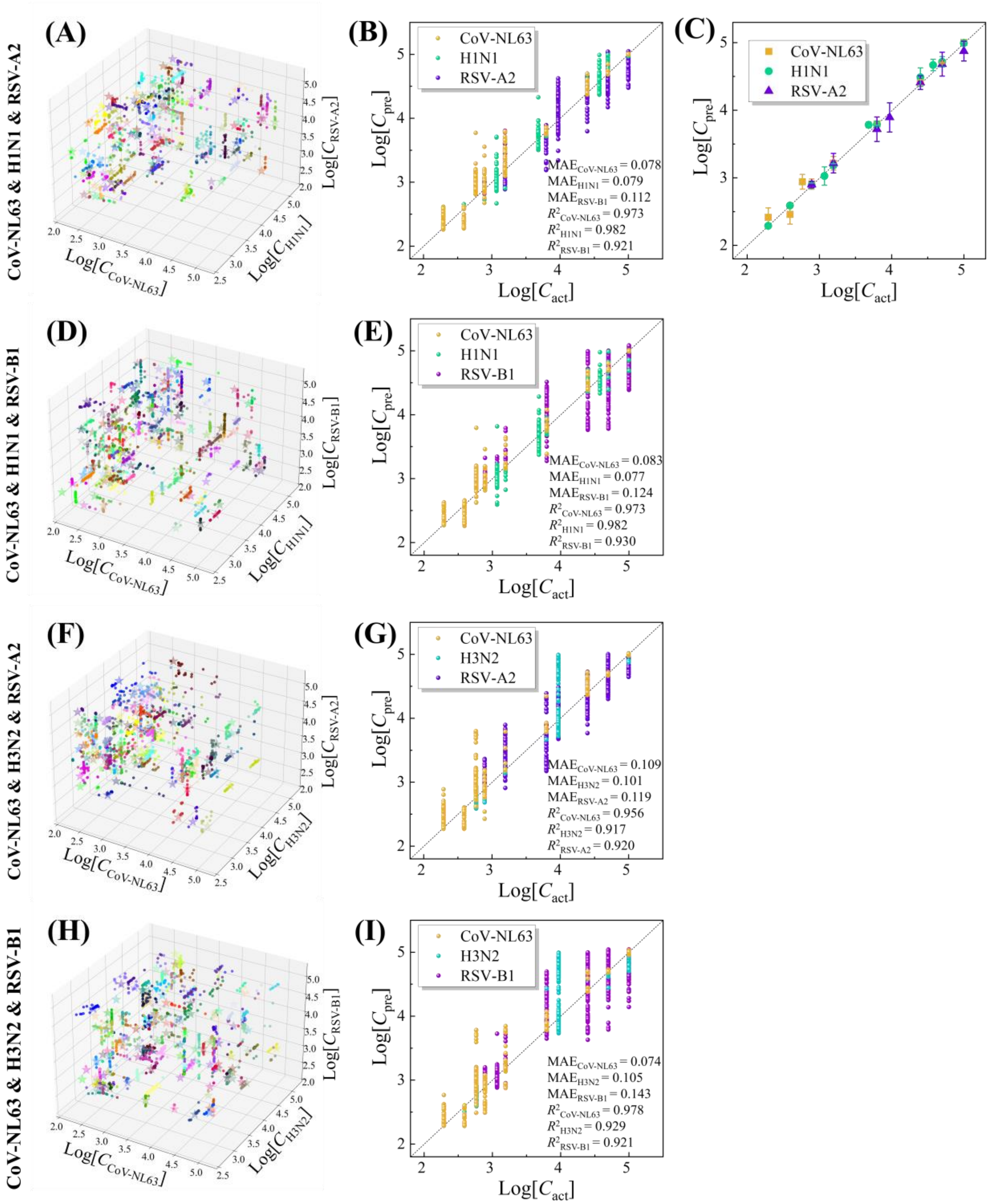

Figure S34. Regression results from the MultiplexCR model for 3VMs in blind tests: CoV-NL63 & H1N1 & RSV-A2. (**A**) A 3D scatter plot of predicted concentrations with *x*-axis for $\log_{10}C_{act}$ of CoV-NL63, *y*-axis for $\log_{10}C_{act}$ of H3N2, and *z*-axis for $\log_{10}C_{pre}$ of RSV-A2. The stars with different colors represent the true virus concentration values for different concentration combinations according to the experiment design. (**B**) A re-plot of all the regression results for

CoV-NL63, H3N2, and RSV-A2, *x*-axis is actual concentration $\log_{10}C_{\mathrm{act}}$, *y*-axis is the predicted concentration $\log_{10}C_{\mathrm{pre}}$, results from same true virus concentrations from different specimens are combined. (**C**) Variations in predicted concentrations based on (B). Similar plots are shown for the regression results of CoV-NL63 & H1N1 & RSV-B1 in (**D**)-(**E**), CoV-NL63 & H3N2 & RSV-A2 in (**F**)-(**G**), and CoV-NL63 & H3N2 & RSV-B1 in (**H**)-(**I**).

Section S13. The reason for re-training the model.

For comparison, the final model, trained on datasets containing SERS spectra of $C_{\text{virus}} \geq$ LODs, achieved a significantly higher accuracy of 95.8% in blind tests, compared to 88.3% for the original model trained on datasets containing SERS spectra of all concentrations in experiments. As shown in **Table S8**, the original model tends to have higher MAEs and a wider range of R^2 values, e.g., Ad5 (**Figure S35A**) and CoV-NL63 & Flu B (**Figure S35B**). In contrast, the final model consistently delivered lower MAEs and higher R^2, as observed in the narrower red violin plots in **Figure S35C**. For the spectra collection point of view, a comprehensive experiment design with lower virus concentration settings is common. However, removing the SERS spectral dataset of virus mixtures with lower LODs and then re-training the deep learning model may be necessary, which can get better prediction accuracy and regression results. This performance improvement comes from the exclusion of low-concentration data, which often introduces noise and inaccuracies, causing the model to generalize poorly. Lower LOD data can also increase the rates of false positives and false negatives, confusing the model and leading to errors. Additionally, training on this noisy data can force the model to learn unnecessarily complex patterns, risking overfitting. By focusing on higher-quality data, the final model enjoys a more stable training process, reducing overfitting, simplifying the model architecture, and maintaining balanced learning. This approach enhances prediction accuracy and overall performance, making the model more reliable and effective for virus co-infection detection in real-world applications.

Table S8. The comparison of model performance.

	Final model		Original model	
**Virus name**	**MAE**	R^2	**MAE**	R^2
Ad5	0.085	0.989	0.229	0.904
CoV-229E	0.049	0.994	0.047	0.995
CoV-NL63	0.099	0.986	0.098	0.985
CoV-OC43	0.093	0.982	0.152	0.961
Flu B	0.080	0.986	0.158	0.952
H1N1	0.114	0.975	0.081	0.987
H3N2	0.046	0.994	0.092	0.980
HMPV-A	0.075	0.992	0.188	0.949
HMPV-B	0.117	0.978	0.222	0.928
RSV-A2	0.166	0.932	0.448	0.683
RSV-B1	0.053	0.994	0.308	0.805
CoV-NL63 & Flu B (CoV-NL63)	0.096	0.985	0.116	0.979

CoV-NL63 & H1N1 (CoV-NL63)	0.121	0.969	0.153	0.945
CoV-NL63 & H3N2 (CoV-NL63)	0.082	0.979	0.118	0.958
CoV-NL63 & RSV-A2 (CoV-NL63)	0.081	0.985	0.094	0.979
CoV-NL63 & RSV-B1 (CoV-NL63)	0.093	0.984	0.098	0.981
H1N1 & RSV-A2 (H1N1)	0.077	0.979	0.086	0.974
H1N1 & RSV-B1 (H1N1)	0.083	0.975	0.086	0.975
H3N2 & RSV-A2 (H3N2)	0.071	0.981	0.088	0.961
H3N2 & RSV-B1 (H3N2)	0.067	0.986	0.076	0.979
CoV-NL63 & Flu B (Flu B)	0.166	0.958	0.372	0.797
CoV-NL63 & H1N1 (H1N1)	0.059	0.982	0.058	0.986
CoV-NL63 & H3N2 (H1N1)	0.071	0.975	0.108	0.925
CoV-NL63 & RSV-A2 (RSV-A2)	0.116	0.971	0.222	0.910
CoV-NL63 & RSV-B1 (RSV-B1)	0.111	0.971	0.261	0.860
H1N1 & RSV-A2 (RSV-A2)	0.127	0.967	0.223	0.909
H1N1 & RSV-B1 (RSV-B1)	0.108	0.980	0.288	0.882
H3N2 & RSV-A2 (RSV-A2)	0.122	0.970	0.244	0.903
H3N2 & RSV-B1 (RSV-B1)	0.115	0.970	0.218	0.900
CoV-NL63 & H1N1 & RSV-A2 (CoV-NL63)	0.078	0.973	0.078	0.974
CoV-NL63 & H1N1 & RSV-B1 (CoV-NL63)	0.083	0.973	0.086	0.973
CoV-NL63 & H3N2 & RSV-A2 (CoV-NL63)	0.109	0.956	0.106	0.959
CoV-NL63 & H3N2 & RSV-B1 (CoV-NL63)	0.074	0.978	0.063	0.983
CoV-NL63 & H1N1 & RSV-A2 (H1N1)	0.079	0.982	0.081	0.981
CoV-NL63 & H1N1 & RSV-B1 (H1N1)	0.077	0.982	0.079	0.980
CoV-NL63 & H3N2 & RSV-A2 (H3N2)	0.101	0.917	0.098	0.952
CoV-NL63 & H3N2 & RSV-B1 (H3N2)	0.105	0.929	0.094	0.970
CoV-NL63 & H1N1 & RSV-A2 (RSV-A2)	0.112	0.921	0.265	0.679
CoV-NL63 & H1N1 & RSV-B1 (RSV-B1)	0.129	0.930	0.261	0.803
CoV-NL63 & H3N2 & RSV-A2 (RSV-A2)	0.119	0.920	0.225	0.760
CoV-NL63 & H3N2 & RSV-B1 (RSV-B1)	0.143	0.921	0.281	0.755

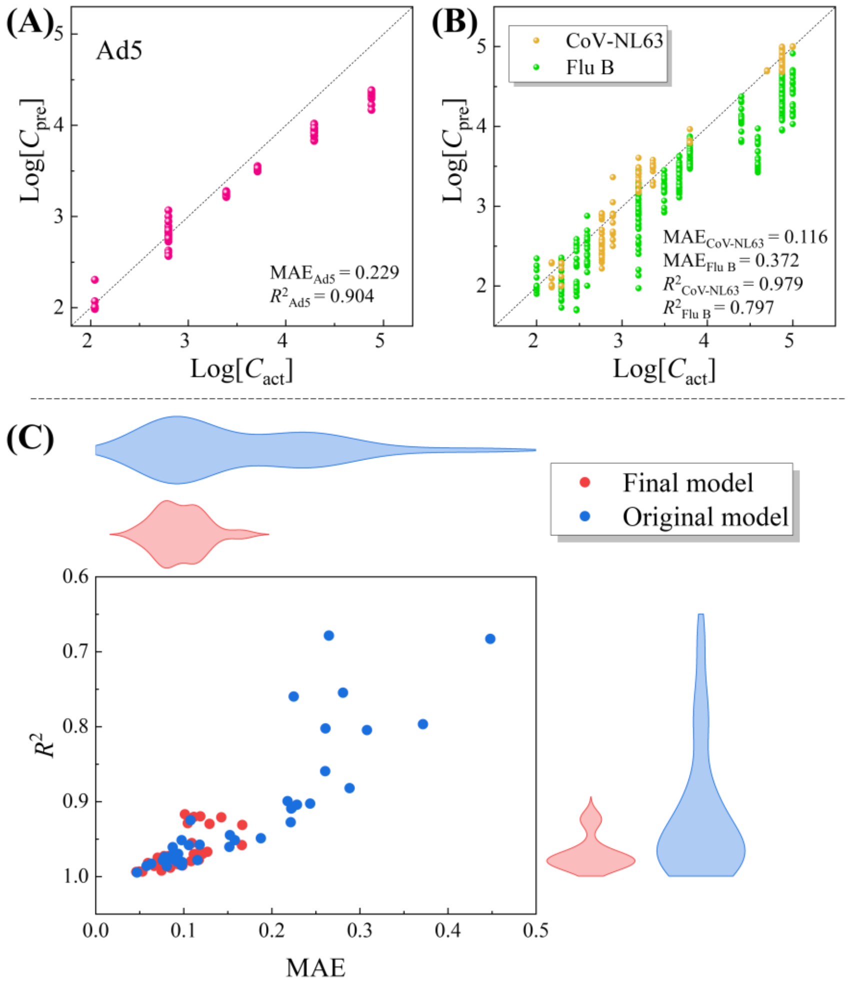

Figure S35. Some representative regression results from the original model of MulitplexCR. (**A**) Ad5 and (**B**) CoV-NL63 & Flu B. (**C**) A comparison of the performance between the final model and original model.

References

1. Pretorius, M. A.; Madhi, S. A.; Cohen, C.; Naidoo, D.; Groome, M.; Moyes, J.; Buys, A.; Walaza, S.; Dawood, H.; Chhagan, M.; Haffjee, S.; Kahn, K.; Puren, A.; Venter, M., Respiratory Viral Coinfections Identified by a 10-Plex Real-Time Reverse-Transcription Polymerase Chain Reaction Assay in Patients Hospitalized With Severe Acute Respiratory Illness—South Africa, 2009–2010. *The Journal of Infectious Diseases* **2012,** *206* (suppl_1), S159-S165.
2. Henegariu, O.; Heerema, N. A.; Dlouhy, S. R.; Vance, G. H.; Vogt, P. H., Multiplex PCR: Critical Parameters and Step-by-Step Protocol. *BioTechniques* **1997,** *23* (3), 504-511.
3. Markoulatos, P.; Siafakas, N.; Moncany, M., Multiplex polymerase chain reaction: A practical approach. *Journal of Clinical Laboratory Analysis* **2002,** *16* (1), 47-51.
4. Elnifro Elfath, M.; Ashshi Ahmed, M.; Cooper Robert, J.; Klapper Paul, E., Multiplex PCR: Optimization and Application in Diagnostic Virology. *Clinical Microbiology Reviews* **2000,** *13* (4), 559-570.
5. Slatko, B. E.; Gardner, A. F.; Ausubel, F. M., Overview of Next-Generation Sequencing Technologies. *Current Protocols in Molecular Biology* **2018,** *122* (1), e59.
6. Liu, L.; Li, Y.; Li, S.; Hu, N.; He, Y.; Pong, R.; Lin, D.; Lu, L.; Law, M., Comparison of Next-Generation Sequencing Systems. *BioMed Research International* **2012,** *2012* (1), 251364.
7. Qin, J.; Li, R.; Raes, J.; Arumugam, M.; Burgdorf, K. S.; Manichanh, C.; Nielsen, T.; Pons, N.; Levenez, F.; Yamada, T.; Mende, D. R.; Li, J.; Xu, J.; Li, S.; Li, D.; Cao, J.; Wang, B.; Liang, H.; Zheng, H.; Xie, Y.; Tap, J.; Lepage, P.; Bertalan, M.; Batto, J.-M.; Hansen, T.; Le Paslier, D.; Linneberg, A.; Nielsen, H. B.; Pelletier, E.; Renault, P.; Sicheritz-Ponten, T.; Turner, K.; Zhu, H.; Yu, C.; Li, S.; Jian, M.; Zhou, Y.; Li, Y.; Zhang, X.; Li, S.; Qin, N.; Yang, H.; Wang, J.; Brunak, S.; Doré, J.; Guarner, F.; Kristiansen, K.; Pedersen, O.; Parkhill, J.; Weissenbach, J.; Antolin, M.; Artiguenave, F.; Blottiere, H.; Borruel, N.; Bruls, T.; Casellas, F.; Chervaux, C.; Cultrone, A.; Delorme, C.; Denariaz, G.; Dervyn, R.; Forte, M.; Friss, C.; van de Guchte, M.; Guedon, E.; Haimet, F.; Jamet, A.; Juste, C.; Kaci, G.; Kleerebezem, M.; Knol, J.; Kristensen, M.; Layec, S.; Le Roux, K.; Leclerc, M.; Maguin, E.; Melo Minardi, R.; Oozeer, R.; Rescigno, M.; Sanchez, N.; Tims, S.; Torrejon, T.; Varela, E.; de Vos, W.; Winogradsky, Y.; Zoetendal, E.; Bork, P.; Ehrlich, S. D.; Wang, J.; Meta, H. I. T. C., A human gut microbial gene catalogue established by metagenomic sequencing. *Nature* **2010,** *464* (7285), 59-65.
8. Martin Denise, A.; Muth David, A.; Brown, T.; Johnson Alison, J.; Karabatsos, N.; Roehrig John, T., Standardization of Immunoglobulin M Capture Enzyme-Linked Immunosorbent Assays for Routine Diagnosis of Arboviral Infections. *Journal of Clinical Microbiology* **2000,** *38* (5), 1823-1826.
9. Gan, S. D.; Patel, K. R., Enzyme Immunoassay and Enzyme-Linked Immunosorbent Assay. *Journal of Investigative Dermatology* **2013,** *133* (9), 1-3.
10. Peeling, R. W.; Olliaro, P. L.; Boeras, D. I.; Fongwen, N., Scaling up COVID-19 rapid antigen tests: promises and challenges. *The Lancet Infectious Diseases* **2021,** *21* (9), e290-e295.
11. Scohy, A.; Anantharajah, A.; Bodéus, M.; Kabamba-Mukadi, B.; Verroken, A.; Rodriguez-Villalobos, H., Low performance of rapid antigen detection test as frontline testing for COVID-19 diagnosis. *Journal of Clinical Virology* **2020,** *129*, 104455.
12. Meyer, B.; Drosten, C.; Müller, M. A., Serological assays for emerging coronaviruses: Challenges and pitfalls. *Virus Research* **2014,** *194*, 175-183.
13. Gärtner Barbara, C.; Hess Ralf, D.; Bandt, D.; Kruse, A.; Rethwilm, A.; Roemer, K.; Mueller-Lantzsch, N., Evaluation of Four Commercially Available Epstein-Barr Virus Enzyme Immunoassays with an Immunofluorescence Assay as the Reference Method. *Clinical and Vaccine Immunology* **2003,** *10* (1), 78-82.
14. McKinnon, K. M., Flow Cytometry: An Overview. *Current Protocols in Immunology* **2018,** *120* (1), 5.1.1-5.1.11.
15. Notomi, T.; Okayama, H.; Masubuchi, H.; Yonekawa, T.; Watanabe, K.; Amino, N.; Hase, T., Loop-mediated isothermal amplification of DNA. *Nucleic Acids Research* **2000,** *28* (12), e63-e63.

16. Kaminski, M. M.; Abudayyeh, O. O.; Gootenberg, J. S.; Zhang, F.; Collins, J. J., CRISPR-based diagnostics. *Nature Biomedical Engineering* **2021,** *5* (7), 643-656.
17. Wang, D.; Coscoy, L.; Zylberberg, M.; Avila, P. C.; Boushey, H. A.; Ganem, D.; DeRisi, J. L., Microarray-based detection and genotyping of viral pathogens. *Proceedings of the National Academy of Sciences* **2002,** *99* (24), 15687-15692.
18. Heller, M. J., DNA Microarray Technology: Devices, Systems, and Applications. *Annual Review of Biomedical Engineering* **2002,** *4* (Volume 4, 2002), 129-153.
19. Leland Diane, S.; Ginocchio Christine, C., Role of Cell Culture for Virus Detection in the Age of Technology. *Clinical Microbiology Reviews* **2007,** *20* (1), 49-78.
20. Falsey Ann, R.; Formica Maria, A.; Walsh Edward, E., Diagnosis of Respiratory Syncytial Virus Infection: Comparison of Reverse Transcription-PCR to Viral Culture and Serology in Adults with Respiratory Illness. *Journal of Clinical Microbiology* **2002,** *40* (3), 817-820.
21. Liu, Y. J.; Chu, H. Y.; Zhao, Y. P., Silver Nanorod Array Substrates Fabricated by Oblique Angle Deposition: Morphological, Optical, and SERS Characterizations. *The Journal of Physical Chemistry C* **2010,** *114* (18), 8176-8183.
22. Song, C.; Chen, J.; Abell, J. L.; Cui, Y.; Zhao, Y., Ag–SiO2 Core–Shell Nanorod Arrays: Morphological, Optical, SERS, and Wetting Properties. *Langmuir* **2012,** *28* (2), 1488-1495.
23. Liu, Y. J.; Zhao, Y. P., Simple model for surface-enhanced Raman scattering from tilted silver nanorod array substrates. *Physical Review B* **2008,** *78* (7), 075436.
24. Liu, Y.-J.; Zhang, Z.-Y.; Zhao, Q.; Dluhy, R.; Zhao, Y.-P., Surface enhanced Raman scattering from an Ag nanorod array substrate: the site dependent enhancement and layer absorbance effect. *The Journal of Physical Chemistry C* **2009,** *113* (22), 9664-9669.
25. Abell, J. L.; Driskell, J. D.; Dluhy, R. A.; Tripp, R. A.; Zhao, Y. P., Fabrication and characterization of a multiwell array SERS chip with biological applications. *Biosensors and Bioelectronics* **2009,** *24* (12), 3663-3670.
26. Boyoglu-Barnum, S.; Todd, S. O.; Meng, J.; Barnum, T. R.; Chirkova, T.; Haynes, L. M.; Jadhao, S. J.; Tripp, R. A.; Oomens, A. G.; Moore, M. L.; Anderson, L. J., Mutating the CX3C Motif in the G Protein Should Make a Live Respiratory Syncytial Virus Vaccine Safer and More Effective. *Journal of Virology* **2017,** *91* (10), e02059-16.
27. Murray, J.; Hogan, R. J.; Martin, D. E.; Blahunka, K.; Sancilio, F. D.; Balyan, R.; Lovern, M.; Still, R.; Tripp, R. A., Probenecid inhibits SARS-CoV-2 replication in vivo and in vitro. *Scientific Reports* **2021,** *11* (1), 18085.
28. Tripp Ralph, A.; Moore, D.; Jones, L.; Sullender, W.; Winter, J.; Anderson Larry, J., Respiratory Syncytial Virus G and/or SH Protein Alters Th1 Cytokines, Natural Killer Cells, and Neutrophils Responding to Pulmonary Infection in BALB/c Mice. *Journal of Virology* **1999,** *73* (9), 7099-7107.
29. Liu, Y. J.; Chu, H. Y.; Zhao, Y. P., Silver Nanorod Array Substrates Fabricated by Oblique Angle Deposition: Morphological, Optical, and SERS Characterizations. *Journal of Physical Chemistry C* **2010,** *114* (18), 8176-8183.
30. Driskell, J. D.; Shanmukh, S.; Liu, Y.; Chaney, S. B.; Tang, X. J.; Zhao, Y. P.; Dluhy, R. A., The Use of Aligned Silver Nanorod Arrays Prepared by Oblique Angle Deposition as Surface Enhanced Raman Scattering Substrates. *The Journal of Physical Chemistry C* **2008,** *112* (4), 895-901.
31. Zhao, Y.; Kumar, A.; Yang, Y., Unveiling practical considerations for reliable and standardized SERS measurements: lessons from a comprehensive review of oblique angle deposition-fabricated silver nanorod array substrates. *Chemical Society Reviews* **2024,** *53* (2), 1004-1057.
32. Yang, Y.; Xu, B.; Murray, J.; Haverstick, J.; Chen, X.; Tripp, R. A.; Zhao, Y., Rapid and quantitative detection of respiratory viruses using surface-enhanced Raman spectroscopy and machine learning. *Biosensors and Bioelectronics* **2022,** *217*, 114721.
33. Liu, Y. J.; Zhang, Z. Y.; Zhao, Q.; Dluhy, R. A.; Zhao, Y. P., Surface Enhanced Raman Scattering from an Ag Nanorod Array Substrate: The Site Dependent Enhancement and Layer Absorbance Effect. *Journal of Physical Chemistry C* **2009,** *113* (22), 9664-9669.

34. Abell, J. L.; Driskell, J. D.; Zhao, Y., Controllable and reversible hot spot formation on silver nanorod arrays. *Chemical Communications* **2014,** *50* (1), 106-108.
35. Fan, J. G.; Dyer, D.; Zhang, G.; Zhao, Y. P., Nanocarpet Effect: Pattern Formation during the Wetting of Vertically Aligned Nanorod Arrays. *Nano Letters* **2004,** *4* (11), 2133-2138.
36. Roman, B.; Bico, J., Elasto-capillarity: deforming an elastic structure with a liquid droplet. *Journal of Physics: Condensed Matter* **2010,** *22* (49), 493101.
37. Chandra, D.; Yang, S., Capillary-Force-Induced Clustering of Micropillar Arrays: Is It Caused by Isolated Capillary Bridges or by the Lateral Capillary Meniscus Interaction Force? *Langmuir* **2009,** *25* (18), 10430-10434.
38. Zhao, Y. P.; Fan, J. G., Clusters of bundled nanorods in nanocarpet effect. *Applied Physics Letters* **2006,** *88* (10), 103123.
39. Yang, Y.; Xu, B.; Haverstick, J.; Ibtehaz, N.; Muszyński, A.; Chen, X.; Chowdhury, M. E. H.; Zughaier, S. M.; Zhao, Y., Differentiation and classification of bacterial endotoxins based on surface enhanced Raman scattering and advanced machine learning. *Nanoscale* **2022,** *14* (24), 8806-8817.
40. Zhang, Z.-M.; Chen, S.; Liang, Y.-Z., Baseline correction using adaptive iteratively reweighted penalized least squares. *Analyst* **2010,** *135* (5), 1138-1146.
41. Shen, Y.; Liang, L.; Zhang, S.; Huang, D.; Deng, R.; Zhang, J.; Qu, H.; Xu, S.; Liang, C.; Xu, W., Organelle-Targeting Gold Nanorods for Macromolecular Profiling of Subcellular Organelles and Enhanced Cancer Cell Killing. *ACS Applied Materials & Interfaces* **2018,** *10* (9), 7910-7918.
42. Shanmukh, S.; Jones, L.; Zhao, Y.-P.; Driskell, J. D.; Tripp, R. A.; Dluhy, R. A., Identification and classification of respiratory syncytial virus (RSV) strains by surface-enhanced Raman spectroscopy and multivariate statistical techniques. *Analytical and Bioanalytical Chemistry* **2008,** *390* (6), 1551-1555.
43. Rygula, A.; Majzner, K.; Marzec, K. M.; Kaczor, A.; Pilarczyk, M.; Baranska, M., Raman spectroscopy of proteins: a review. *Journal of Raman Spectroscopy* **2013,** *44* (8), 1061-1076.
44. Breiman, L., Random Forests. *Machine Learning* **2001,** *45* (1), 5-32.
